# Carbon Fixation by Marine Ultrasmall Prokaryotes

**DOI:** 10.1093/gbe/evz050

**Published:** 2019-03-23

**Authors:** Romain Lannes, Karen Olsson-Francis, Philippe Lopez, Eric Bapteste

**Affiliations:** 1 Sorbonne Université, Institut de Systématique, Evolution, Biodiversité (ISYEB), Sorbonne Université, CNRS, Museum National d’Histoire Naturelle, EPHE, Université des Antilles, Paris, France; 2 School of Environment, Earth and Ecosystems, The Open University, Milton Keynes, United Kingdom; 3 Institut de Systématique, Evolution, Biodiversité (ISYEB), Sorbonne Université, CNRS, Museum National d’Histoire Naturelle, EPHE, Université des Antilles, Paris, France

**Keywords:** metagenomics, marine ultrasmall organisms, metabolism, carbon fixation

## Abstract

Autotrophic carbon fixation is a crucial process for sustaining life on Earth. To date, six pathways, the Calvin–Benson–Bassham cycle, the reductive tricarboxylic acid cycle, the 3-hydroxypropionate bi-cycle, the Wood–Ljungdahl pathway, the dicarboxylate/4-hydroxybutyrate cycle, and the 4-hydroxybutyrate cycle, have been described. Nano-organisms such as members of the Candidate Phyla Radiation (CPR) bacterial superphylum and the Diapherotrites, Parvarchaeota, Aenigmarchaeota, Nanoarchaeota, Nanohalorchaeota (DPANN) archaeal superphylum could deeply impact carbon cycling and carbon fixation in ways that are still to be determined. CPR and DPANN are ubiquitous in the environment but understudied; their gene contents are not exhaustively described; and their metabolisms are not yet fully understood. Here, the completeness of each of the above pathways was quantified and tested for the presence of all key enzymes in nano-organisms from across the World Ocean. The novel marine ultrasmall prokaryotes were demonstrated to collectively harbor the genes required for carbon fixation, in particular the “energetically efficient” dicarboxylate/4-hydroxybutyrate pathway and the 4-hydroxybutyrate pathway. This contrasted with the known carbon metabolic pathways associated with CPR members in aquifers, where they are described as degraders (Castelle CJ, et al. 2015. Genomic expansion of domain archaea highlights roles for organisms from new phyla in anaerobic carbon cycling. Curr Biol. 25(6):690–701; Castelle CJ, et al. 2018. Biosynthetic capacity, metabolic variety and unusual biology in the CPR and DPANN radiations. Nat Rev Microbiol. 16(10):629–645; Anantharaman K, et al. 2016. Thousands of microbial genomes shed light on interconnected biogeochemical processes in an aquifer system. Nat Commun. 7:13219.). Our findings suggest that nano-organisms have a broader contribution to carbon fixation and cycling than currently assumed. Furthermore, CPR and DPANN superphyla are possibly not the only nanosized prokaryotes; therefore, the discovery of new autotrophic marine nano-organisms by future single cell genomics is anticipated.

## Introduction

Autotrophic carbon fixation is a crucial process for sustaining life on Earth as it fixes inorganic carbon, including the sequestration of atmospheric carbon dioxide (De La Rocha and Passow 2014), into organic carbon (Hügler and Sievert 2011). It is responsible for the annually net fixation of 7 × 10^16^ g carbon, which corresponds to the conservation of 2.8 × 10^18^ kJ of energy (Berg 2011). To date, there are six known pathways for autotrophic carbon fixation. This includes the Calvin–Benson–Bassham (CBB) cycle, which is quantitatively the most important mechanism of autotrophic CO_2_ fixation in nature and is primarily achieved by photosynthetic organisms (Hügler and Sievert 2011). For many years, it was thought to be the only pathway for autotrophic CO_2_ fixation, but more recently five additional pathways have been described. These include the reductive tricarboxylic acid cycle (rTCA), the 3-hydroxypropionate bi-cycle (HBC), the reductive acetyl-CoA pathway, which is also known as the Wood–Ljungdahl pathway (WL), the dicarboxylate/4-hydroxybutyrate cycle (DH), and the 4-hydroxybutyrate cycle (Hügler and Sievert 2011). Concurrently, an increasing number of models have been developed that highlight the role of micro-organisms in carbon fixation (Wieder et al. 2015; Dyksma et al. 2016; Guidi et al. 2016; Larhlimi et al. 2016; La Cono et al. 2018). For example, *Prochlorococcus*, a small and extremely abundant photosynthetic cyanobacterium, was proposed to be a key contributor to autotrophic carbon fixation in the ocean (Partensky et al. 1999). Similarly, SAR11, one of the tiniest known photoheterotrophic organisms (cell volume of roughly 0.01 µm^3^), seems to play an important ecological role as the most abundant marine planktonic organism (Rappé et al. 2002; Giovannoni 2017).

Importantly, studies of environmental microbes show that microbial diversity is still largely underexplored (Brown et al. 2015; Castelle et al. 2015; Parks et al. 2017). Recently, the number of described prokaryotic lineages doubled with the discovery of novel superphyla including some ultrasmall members: the Candidate Phyla Radiation (CPR; bacteria) and the Diapherotrites, Parvarchaeota, Aenigmarchaeota, Nanoarchaeota, Nanohalorchaeota (DPANN; archaea) (Rinke et al. 2013; Brown et al. 2015; Luef et al. 2015; Hug et al. 2016). The physiology of these ultrasmall prokaryotes (hereafter called nano-organisms) is unusual, not only because of their reduced cell volume (these cells can pass through 0.22-µm filters, a size usually expected to exclude most micro-organisms) (Andrew et al. 1999; Luef et al. 2015) but also because of their reduced genome size and biosynthetic capability. Most of the CPR lack parts of central metabolic pathways, including nucleotide and amino acid biosyntheses (Brown et al. 2015; Castelle et al. 2015). Nano-organisms also have an incomplete tricarboxylic acid cycle and lack NADH dehydrogenase and electron transport chains (Brown et al. 2016).

Consequently, the potential role of these nano-organisms in the geochemical cycle of carbon and hydrogen (Anantharaman et al. 2016) has begun to be investigated. For example, Anantharaman et al. detected the presence of key enzymes involved in the carbon, nitrogen, sulfur, and hydrogen cycles in local metagenomic data from aquifers located in Rifle (USA, Colorado), which were assigned to the CPR superphylum. Likewise, in the same aquifers, Rubisco type II/III genes were found. These genes seemed to be active in the CPR and DPANN superphyla (Wrighton et al. 2016) suggesting the presence of the nucleotide salvaging pathway and potentially the CBB pathway. Yet, the phylogenetic and functional diversities of nano-organisms are possibly not fully appreciated and in particular their role in carbon fixation remains to be characterized. In this broad-scale study, the possible role of some known and novel candidate nano-organisms in ocean carbon fixation was investigated, specifically from sites that were sampled as part of the TARA OCEAN expedition (Sunagawa et al. 2015). First, an in silico approach was used to retrieve putative sequences of nano-organisms from the TARA OCEAN metagenome data sets and analyze their phylogenetic diversity. Second, prokaryotic carbon fixation pathways that were described in KEGG were used to identify homologs in marine nano-organisms. Finally, the completeness and geographical distributions of homologs from these autotrophic carbon fixation pathways in 65 of the TARA sampling sites were analyzed.

## Materials and Methods

### Selection of Sequences

**Fig. 1.**
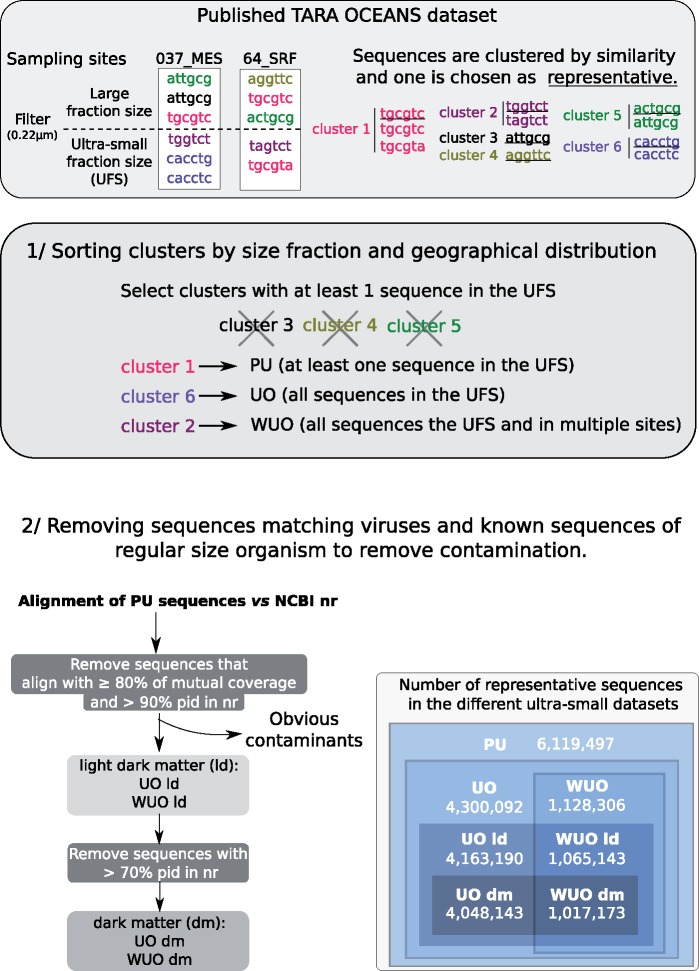
—Ultrasmall data set filtration. The raw TARA Oceans data set includes more than 140 million sequences that have been clustered to 44 million sequences (Sunagawa et al. 2015). Each cluster has a representative sequence that may represent many sequences. If a cluster contains at least one sequence from the ultrasmall size fraction then this cluster was selected as part of the PU data set. If a PU cluster only contained sequences from the ultrasmall size fraction, this cluster was also assigned to the UO data set. If a UO cluster included sequences from at least two different sampling sites, this cluster was also assigned to a WUO data set. Then, PU sequences were aligned against NCBI nr in order to remove potential virus sequences. Furthermore, these alignments were used to remove potential contamination by known large micro-organisms from the UO and WUO data sets. The light dark matter and dark matter UO and WUO data sets, respectively, were defined by removing sequences with at least 80% of mutual cover and 90% %ID (light dark matter) or at least 80% of mutual cover and 70% %ID (dark matter) to sequences in the NCBI nr database. Numbers of sequences in each data set are shown in the box.

The sequences used in this study were obtained from the TARA OCEAN metagenomic database (ftp://ftp.sra.ebi.ac.uk/vol1/ERA412/ERA412970/tab/OM-RGC_seq.release.tsv.gz), which is publicly available. The database consists of sequencing data from various sampling sites, depths, and fraction sizes, including an ultrasmall size fraction (<0.22 µm). About a hundred million sequences of predicted proteins have already been clustered by similarity using CD-HIT (Li and Godzik 2006; Fu et al. 2012). These clusters were sorted for two reasons: first, to decontaminate the ultrasmall size-fraction data set from TARA OCEANS. Second, to characterize the microbial dark matter in the ultrasmall size fraction (by identifying genes from candidate ultrasmall prokaryotes, increasingly different from known reference taxa). To do so, each sequence within each cluster of similarity was assigned to a size fraction of origin. Clusters without sequences from the ultrasmall size fraction were discarded from the rest of our analyses. The 6,677,440 remaining clusters included at least a representative sequence from the ultrasmall size fraction (<0.22 µm). As such clusters were not necessarily strictly associated with the ultrasmall size fraction, they were therefore called the “Potentially Ultrasmall” (PU) data set. Problematically, sequences from the PU data set were not necessarily sequenced from bona fide ultrasmall prokaryotes and may have resulted from contamination of the ultrasmall size fraction, for example, from the presence of free DNA from regular-sized prokaryotes or viruses. Therefore, a further level of stringency was used, to define UO data set (for “Ultrasmall Only”), nested in the PU data set. The UO data set included all sequences from the PU data set that were exclusively found in samples from the ultrasmall size fraction. Among the 4,586,489 clusters from UO, 1,258,638 clusters contained sequences found at more than one site. We called this latter category of widespread clusters WUO (Widespread Ultrasmall Only) (fig. 1).

The clusters from PU, UO, and WUO were further curated by detecting viral proteins through similarity searches against the NCBI nr database (March 2017) using DIAMOND (Buchfink et al. 2015). This removed 286,388 and 130,330 potential viral proteins from the UO and WUO clusters, respectively. An additional search was performed against the sequences from the TARA ocean metavirome (project PRJEB6606, European Nucleotide Archive [https://www.ebi.ac.uk/ena]) to identify potential environmental contaminants. This resulted in the removal of 142 sequences. Notably, autotrophic carbon fixation genes returned no matches with ≥80% sequence identity and a mutual alignment coverage of ≥80%, indicating that there was no positive evidence that these carbon fixation genes were carried by marine viruses.

Most annotated prokaryotes known to date, with the exception of nanosized members of the CPR and DPANN superphyla, were not expected to pass through a 0.22-µm filter. Therefore, the finding of proteins in the UO/WUO data sets that were highly similar to known regular-sized prokaryotes was likely due to contamination. For each sequence in our data set, the percentage of identity (%ID) to its best hit in nr was considered using DIAMOND. This was carried out in order to quantify how similar the environmental sequence was to a reference sequence. The step of taxonomic annotation allowed us to classify the environmental sequences from the UO and WUO clusters into two levels of increasing divergence from a reference, looking for potential organismal dark matter in the ultrasmall size fraction. Thus, the UO and WUO data sets were split into two nested categories: “dark matter” and “light dark matter.” Sequences whose best hit against nr showed a mutual coverage >80% and %ID <90% were assigned to “light dark matter” (4,300,092 sequences for UO and 1,065,606 sequences for WUO); whereas sequences that showed %ID <70% %ID were assigned to “dark matter” (4,048,143 sequences for UO and 1,017,137 sequences for WUO). Furthermore, sequences taxonomically assigned to DPANN, unclassified bacteria, unclassified archaea, CPR, candidate or “root: unassigned” were assigned to both “light dark matter” and “dark matter,” because these taxa likely correspond to bona fide ultrasmall prokaryotes. The “dark matter” clusters provided an additional perspective, but “light dark matter” clusters were a priori not more (or less) contaminated than “dark matter” clusters.

### Mining the KEGG Database

Using the KEGG database (Ogata et al. 1999), a list of KEGG Orthology terms was defined, which corresponded to metabolic pathways associated with autotrophic carbon fixation (M00173, M00374, M00375, M00376, M00377, M00579, and M00620), as well as ribosomal complexes in eukaryotes (M00177), archaea (M00179), and bacteria (M00178). All corresponding proteins (179,853 proteins for carbon fixation, and 211,781 proteins for ribosomal complexes) were retrieved using the Uniprot mapping tool (http://www.uniprot.org/mapping/) or the KEGG API service (March 2017).

### Homology Detection and Taxonomic Annotation

The homologs of KEGG proteins that were present in the PU, UO, and WUO data sets were identified using NCBI BLAST (version 2.6.0) (Camacho et al. 2009). The following criteria were used to assess homology: %ID > 25%, *E*-value <1e-5, and mutual alignment coverage >70% (Alvarez-Ponce et al. 2013; Haggerty et al. 2014). Using these thresholds, 20,368 sequences from the TARA ultrasmall data set were detected as homologs of proteins from autotrophic carbon fixation pathways. Additionally, using the same methodology 37,054 sequences from the PU data set were detected as homologs to proteins from ribosomal complexes.

### Pathways Completeness

A KEGG pathway describes a set of reactions (modules), which require a set of enzymes (supplementary table 2, Supplementary Material online). For each sampling site, if homologs of the required enzymes existed in a data set, the module was considered present (namely, in UO, UO “light dark matter,” UO “dark matter,” WUO, WUO “light dark matter,” and WUO “dark matter”) suggesting that the ultrasmall prokaryotes could complete that step of the pathway. Optional enzymes were not considered but were reported if found. Finally, the percentage of modules present at a given site was taken as a proxy of the completeness of the pathway. Key enzymes (according to Berg [2011]) and key modules (i.e., modules that contain at least one key enzyme) were highlighted.

### Correlation between Pathway Completeness and Sampling Effort

Correlations between a pathway’s completeness and various assessments of the sequencing effort were computed with a Spearman correlation test, using the python3 function spearmanr from the SciPy library. Assessments of the sequencing effort for a given site were provided as the number of reads, high quality reads, predicted genes, and average read coverage per protein.

### Taxonomic Enrichment or Depletion of Filtered Data Sets

For each data set, the number of proteins assigned to a taxonomic group was compared with the PU data set. A pairwise Fisher exact test (using fisher_exact function from Scipy.stats Python3 library) was used to compare each taxonomic group with the remaining groups to identify significant enrichment or depletion compared with the PU data set. Because nine taxonomic groups were tested with six data sets, the corresponding Bonferroni correction was applied to the *P* values for a 5% type I error.

### Phylogenetic Analyses

Reference sequences from KEGG and their environmental homologs were aligned with DIAMOND (Buchfink et al. 2015). A sequence similarity network was built from these alignments in order to define gene families (Corel et al. 2016), with >=80% mutual coverage and >=30% %ID as thresholds for edges. Gene families were defined as connected components in this sequence similarity network. All key enzymes of the autotrophic carbon fixation pathways, as well as ribosomal proteins from connected components with more than 100 sequences, were selected for diversity and phylogenetic analyses. Homologs from all published CPR and DPANN genomes were added (2,481,154 sequences as of December 2018) using DIAMOND (>80% coverage, >30% %ID). The resulting gene families were aligned using MAFFT (Katoh et al. 2002) and the alignments were trimmed using trimAl (Capella-Gutiérrez et al. 2009) with default parameters. Maximum likelihood trees were reconstructed using IQ-Tree (Trifinopoulos et al. 2016) under the LG + G model, and 1,000 ultrafast bootstraps replicates were performed (Minh et al. 2013).

## Results

**Fig. 2.**
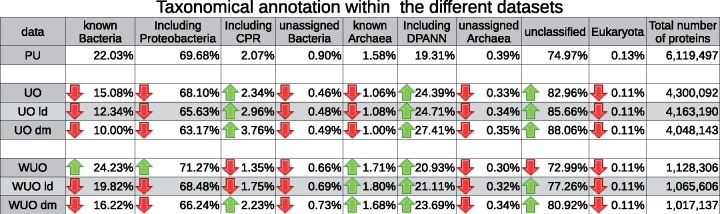
—Effect of filtration on data sets phylogenetic composition. Each row represents a data set after filtration. Known Bacteria, Archaea, and Eukaryota represent sequences that show a best hit in a BLAST search against the NCBI nr database that is referenced as Bacteria, Archaea, and Eukaryote, respectively. Unclassified sequences were environmental sequences that had no hits in the NCBI nr database or were annotated as “root; unclassified sequences”. Unassigned Bacteria and Archaea sequences were closely related to sequences in the NCBI nr database that were only annotated at the domain level. “Including Proteobacteria” and “Including CPR” represented the percentage of Known Bacteria for which best hits in NCBI nr were annotated as Proteobacteria or as CPR, respectively. Including DPANN represented the percentage of Known Archaea for which best hits in NCBI nr database are annotated as a DPANN. For each data set, the effect of filtration on phyla proportion was investigated. Green and red arrows indicated phyla proportions that were significantly enriched or depleted, respectively, for a given phylum in a given data set compared with the proportion of that phylum in PU. Abbreviations: PU, Potentially Ultrasmall; UO, Ultrasmall Only; WUO, Widespread Ultrasmall Only; ld, light dark matter; and dm: dark matter.

Twenty thousand three hundred sixty-eight environmental homologs sequences were identified for six autotrophic carbon fixation pathways, at a threshold of sequence identity >25%, of mutual coverage >70%, and *E*-value <1e-5 (supplementary table 2, Supplementary Material online, and Materials and Methods). Some active micro-organisms can pass through a 0.22-µm filter (Hasegawa et al. 2003), particularly as “starvation forms” (Haller and Ro 1999). A screening step was added to identify potential contamination, that is, to remove sequences from organisms larger than nano-organisms and viruses. As a result, nested data sets of environmental sequences were produced, which were exclusively found in the ultrasmall fraction and defined with increasingly stringent conditions of geographic and taxonomic distributions (fig. 1). With respect to the original data sets associated with the ultrasmall size fraction of the TARA OCEANS project, the “cleaned” data sets developed in this study were significantly enriched in taxonomically unclassified sequences, and in CPR and DPANN sequences. They were also depleted in unassigned archaea and bacteria, and in known regular-sized bacterial phyla and in viruses (fig. 2, *P* values supplementary table 1, Supplementary Material online). The proportion of known archaeal lineages was unaffected by this screening process.

**Fig. 3.**
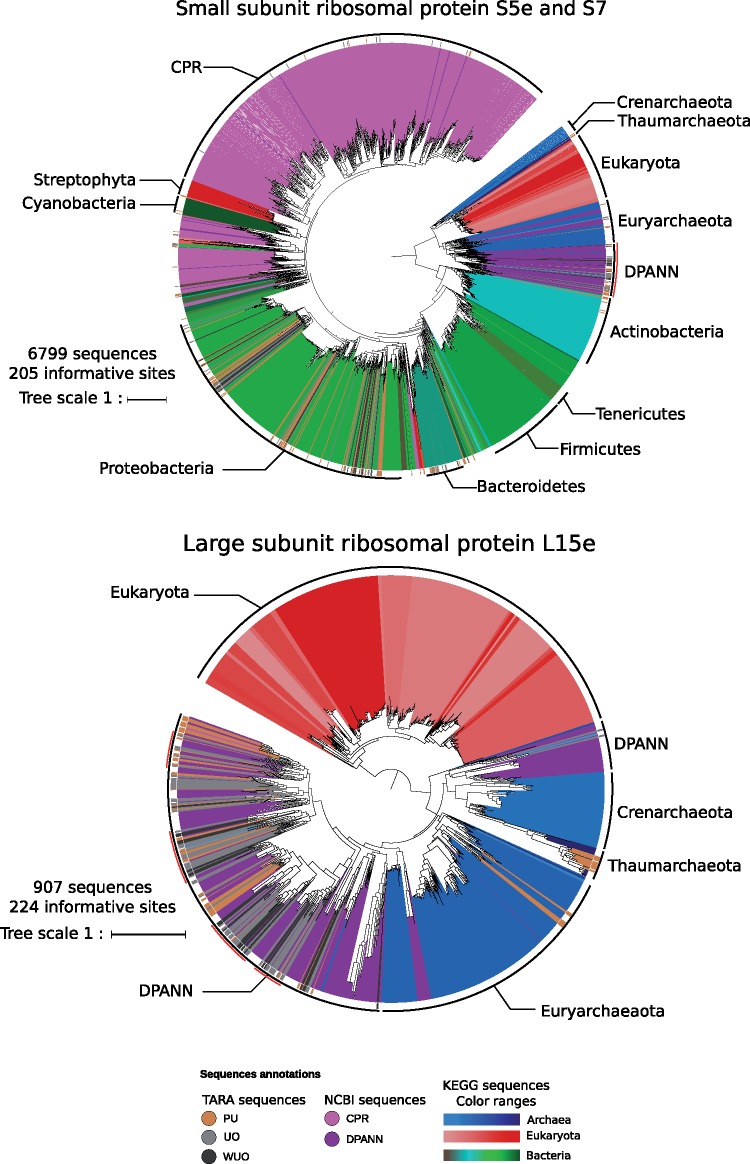
—Phylogenetic trees of ribosomal proteins S5e and S7 (top) and L15e (bottom). Sequences were aligned using MAFFT in auto mode and trimmed with TrimAl. Trees were constructed using IQ-TREE with LG + G4 models and ultrafast bootstrap approximation (Minh et al. 2013). The trees were rooted between Archaea and Bacteria and branches with bootstrap values <50% were collapsed. The number of informative sites and branch length scale bars (substitutions per site) are shown. Environmental sequences are highlighted by a colored bar in the outer ring. The sequences were from three sources: 1) environmental sequences from the TARA Oceans data sets; 2) CPR and DPANN sequences from assemblies available in NCBI; and 3) other reference sequence from KEGG. Sequences are colored by taxonomic annotation. Archaeal sequences found in 037, 038, and 039 MES sampling sites are highlighted by a red arc. Abbreviations: PU, Potentially Ultrasmall; UO, Ultrasmall Only; and WUO: Widespread Ultrasmall.

**Fig. 4.**
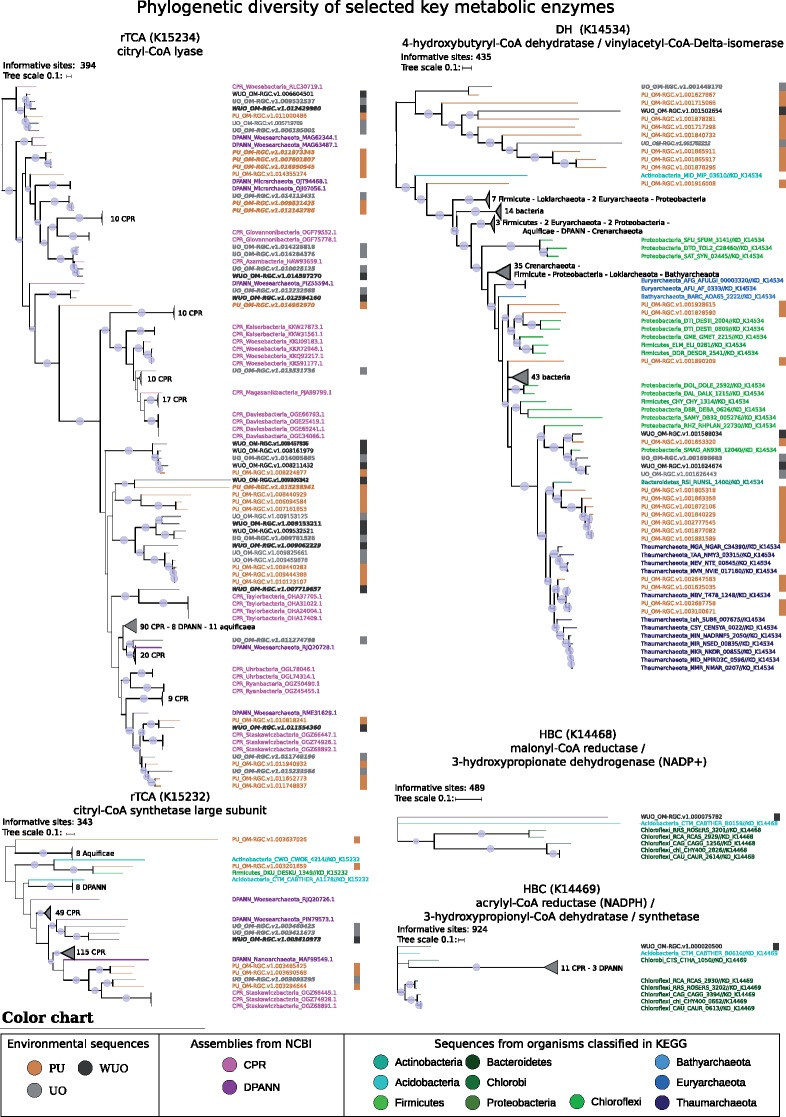
—Phylogenetic trees of selected key enzymes in three carbon fixation pathways. Trees were reconstructed using maximum likelihood on trimmed alignments. The number of informative sites and branch length scale bars (substitutions per site) are shown for each tree. Sequences used are from KEGG, NCBI (CPR and DPANN) and from the TARA Oceans data set. Trees were midpoint rooted. Bootstraps were computed using 1,000 iterations of ultrafast bootstrap approximation. Branches with bootstrap <50% were collapsed, a light blue dot highlights branches with bootstrap values >80%. Environmental sequences are highlighted by a colored bar on the right of each tree. Sequence names: environmental sequences were formatted as (PU, UO, WUO)TARA identifier. KEGG sequences were formatted as phylum_KEGG_identifier. NCBI sequences were formatted as (CPR/DPANN)_phylum_proteinID. For readability, some clades were collapsed and are represented by a dark triangle with the description of the clade’s sequences. Abbreviations: rTCA, reductive tricarboxylic acid cycle; DHC: dicarboxylate–hydroxybutyrate cycle; HBC, 3-hydroxypropionate bi-cycle; PU, Potentially Ultrasmall; UO, Ultrasmall Only; and WUO, Widespread Ultrasmall Only.

Our filtered data sets were phylogenetically rich in diversity of presumed ultrasmall prokaryotes. This was assessed by careful analysis of the placement of the ultrasmall prokaryotes in the maximum likelihood phylogenies of ribosomal proteins (fig. 3 and supplementary fig. 1, Supplementary Material online). In these trees, oceanic ultrasmall prokaryotes did not appear to be monophyletic. Rather, they were related to various known prokaryotic lineages, such as CPR and DPANN, but also less expectedly to Bacteroidetes and Proteobacteria. This suggested that either some contamination is retained in the filtered data sets or there are genuine ultrasmall members of these clades that are yet to be described. Moreover, some of the environmental sequences that qualified as “light dark matter” and as “dark matter” clustered in these phylogenies, hinting at undescribed ultrasmall lineages within known major prokaryotic groups. Phylogenies of key enzymes involved in carbon fixation showed similar results: sequences from the ultrasmall size fraction branched within different major prokaryotic groups, pointing to new groups within CPR, DPANN and other prokaryotic clades (fig. 4 and supplementary fig. 2, Supplementary Material online). This latter result suggests that unknown ultrasmall prokaryotes could take part in aspects of carbon fixation. For example, a widespread environmental lineage related to *Chloroflexi* and *Acidobacteria* was found to host homologs to both the malonyl-CoA reductase/3-hydroxypropionate dehydrogenase (NADP+) enzyme, fumarate hydratase, class II and the acrylyl-CoA reductase (NADPH)/3-hydroxypropionyl-CoA dehydratase/synthetase, suggesting a potential contribution to the HBC pathway (fig. 4 and supplementary fig. 2, Supplementary Material online).

**Fig. 5.**
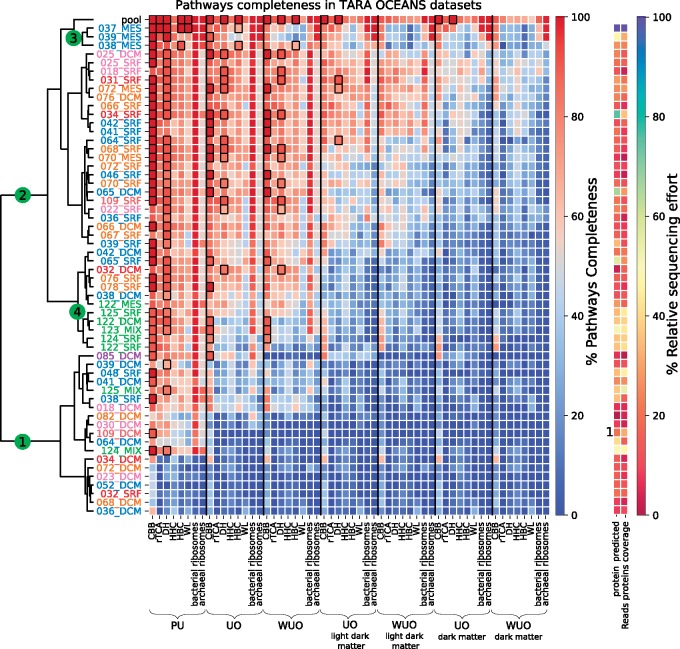
—Heatmap of completeness of six carbon fixation pathways and archaeal and bacterial ribosomal complexes. The heatmap color scale shows the completeness of pathways or ribosomal complexes, with rows as sampling sites and columns as proteins sets. Black squares highlight sites with pathway completeness >60% and comprising all key enzymes. Rows were clustered using scipy.cluster.hierarchy.linkage (“ward” method). The corresponding dendrogram is shown to the left of the heatmap. Row names indicate sampling sites in the format TARA sampling site id (three digits) _ depth. Depths are: SRF (Surface), DCM (Deep Chlorophyll Maximum), MES (Mesopelagic), and MIX (mixed). Row label colors represent oceanic regions: brown for North Pacific Ocean, green for South Pacific Ocean, purple for Southern Ocean, orange for South Atlantic Ocean, dark blue for Indian Ocean, red for Red Sea, and pink for Mediterranean Sea. The “pool” row represents results for all sampling sites pooled together. Ribosomal complexes from bacteria and archaea contain 55/67 proteins, respectively, and share 31 proteins. Sequencing effort is computed as the proportion of the number of proteins found at a given site and the average number of reads per protein, relatively to the values found at 037_MES sampling site, which showed the maximum values for both indicators.

To obtain a more comprehensive view of their ecological role, the geographic distribution of the environmental sequences from the ultrasmall prokaryotes and their potential to include complete autotrophic carbon fixation pathways was investigated. A heatmap (fig. 5) was produced, which represented the completeness of each of the six carbon fixation pathways analyzed in this study, as well as the completeness of bacterial and archaeal ribosomal complexes. Bacterial and archaeal ribosomal complexes are composed of a comparable number of proteins to the carbon fixation pathways and were therefore used as positive controls to validate this method for detecting full-sized pathways. It was expected that ribosomal complexes would appear to be complete in a site if a sufficient sequencing effort had occurred. For each site, the number of reads and proteins predicted to be part of a ribosomal complex or an autotrophic carbon fixation pathway, and the average read coverage of these proteins, was reported. However, the information relative to the sampling effort associated with each site could warrant naïve assumptions regarding the ultrasmall prokaryotes and carbon fixation pathways, especially when undersampling could be a plausible explanation (supplementary table 3, Supplementary Material online). Conversely, the detection of homologous enzymes from carbon fixation pathways in generally undersampled sites suggested that ultrasmall prokaryotes were involved in these ecologically important pathways. The columns of the heatmap (fig. 5) represent the data sets sorted by increasing stringency; the rows represent samplings sites and were hierarchically clustered according to the completeness of their pathways, in order to search for possible geographical trends.

Interestingly, the heatmap revealed two major clusters of sampling sites. The lower cluster (dot 1 in fig. 5) corresponded to sites in which no, or very few, homologs of the carbon fixation proteins were detected (with the exception of the CBB pathway, which appeared to be partly present at all levels of stringency). Sites sampled at the deep chlorophyll maximum depth were overrepresented in this part of the heatmap; however, the incompleteness was not due to the amount of sequencing data compared with other sites, both in terms of proportion of proteins and average read coverage per protein. In addition, there were no homologs of ribosomal proteins at these sites, which suggests that the samples associated with the lower part of the heatmap were largely viral rather than microbial (as expected for an ultrasmall size fraction). By contrast, the higher cluster (dot 2 in fig. 5) of the heatmap was enriched in sites from the surface depths. The finer-grained clustering of sampling sites within this part of the heatmap points to some local geographical patterns. First, samples 037, 038, and 039 from mesopelagic depths clustered together (dot 3 in fig. 5) corresponding to sites in the Indian Ocean, which had similar distributions of carbon fixation pathways and ribosomal complexes. These three sites presented a rich proportion of archaeal complexes, even in data sets with very stringent thresholds. This hinted at the presence of still undescribed ultrasmall archaea in the Indian Ocean, which were indeed detected in 29 individual phylogenies of ribosomal proteins (fig. 2 and supplementary information, Supplementary Material online). These potentially new ultrasmall archaea were generally polyphyletic, and some were often related to an archaeon GW2011 AR20, assigned to the DPANN superphyla (Castelle et al. 2015). A similar cluster was also detected from sites from the South Pacific Ocean (dot 4 in fig. 5).

In terms of pathways completeness, the CBB and DH pathways were the most commonly complete carbon fixation pathways, even with the requirement that homologous enzymes should be found at multiple sites. This suggests that ultrasmall prokaryotes are primary involved in these two pathways. The presence of the DH pathway is particularly noteworthy because several enzymes of the pathway are sensitive to oxygen and this rare pathway is strictly anaerobic (Berg, Kockelkorn, et al. 2010). This is consistent with the DH pathway being found in anaerobic crenarchaeal orders *Thermoproteales* and *Desulfuroccocales* (Berg, Ramos-Vera, et al. 2010), and possibly present in “marine group I archaea” *Thaumarchaeota* (Könneke et al. 2014).

Moreover, the four remaining pathways were also found with more than 50% completeness in multiple sampling sites, especially in the top portion of the heatmap. This observation was particularly interesting as the complete HBC pathway uses dissolved bicarbonate HCO_3_^−^ as a starting substrate. However, complete or even rudimentary HBC can co-assimilate trace amounts of organic compounds such as fermentation products (acetate, propionate and succinate) and numerous other compounds that are metabolized through acetyl-CoA and propionyl CoA (Zarzycki et al. 2009). Such characteristics make HBC well suited for a parasitic or symbiotic lifestyle, because a nanoparasite with HBC could in principle fix (in)organic carbon and share organic carbon with its host. Whereas bacteria from the CPR superphylum have been described as likely symbionts or parasites, no CPR members harboring a HBC pathway have been described thus far (Kantor et al. 2013; Gong et al. 2014; Brown et al. 2015; Nelson and Stegen 2015). This suggests that nano-organisms may use either the complete or partial HBC pathway, even if this pathway was not observed in newly assembled genomes from TARA OCEANS (Tully et al. 2018).

Finally, when sequences from all sites were pooled together to produce an overall picture of the metabolic potential of ultrasmall prokaryotes, sequences associated with ultrasmall size fraction encoded a large fraction of the autotrophic carbon fixation pathways. The completeness of both carbon fixation pathways and ribosomal complexes decreases as data sets become more stringent, likely because of the reduction in the overall size of the data set. However, six sites (in majority from the surface or SRF) still included more than 50% of the enzymes involved in the HHC pathway within the set of sequences associated with the ultrasmall microbial “dark matter.” By contrast, little evidence of a complete WL pathway in the ultrasmall “light dark matter” and in the ultrasmall “dark matter” was found, although the WL pathway is thought to be the ancestral and the most energetically efficient autotrophic carbon fixation pathway.

In sampling sites with high pathway completeness (≥60%), further investigations were carried out to identify key enzymes, that is, enzymes that were specific to a metabolic pathway and thought to have appeared once during evolution (Berg 2011). The presence of all key enzymes of a metabolic pathway, together with a high completeness, strongly suggested the occurrence of that metabolic pathway in the environment. In the PU data set, the key enzymes for the CBB, rTCA, DH, HBC, and WL pathways were identified in some sites (Berg, Ramos-Vera, et al. 2010; Berg 2011). The distribution of CBB and DH pathways appeared widespread, whereas rTCA and WL were only found in the Indian Ocean cluster (dot 3 in fig. 5). Of note, the rTCA pathway is the second least expensive cycle after WL, using two ATPs, making it suitable for fermenting organisms to utilize. Several rTCA enzymes are sensitive to oxygen, restricting rTCA activity to anaerobic or low oxygen environments. In the UO data sets, the key enzymes for the CBB, DH, and HBC pathways were detected, but the HBC pathway was restricted to two sampling sites (dot 3 in fig. 5). In the WUO data sets, key enzymes for the CBB pathway were found in 12 sites and the DH pathway was found in 10 sites, whereas the HBC was only found in 1 (038 at mesopelagic depth). However, the presence of the HBC pathway in nano-organisms deserves further investigation. Recent articles (Shih et al. 2017) suggest different key enzymes for HBC than those used here (Berg 2011), and homologs of some of these alternative enzymes have been found in our most stringent data set WUO “dark matter” (K08691 28 sequences, K09709 19 sequences, and K14449 7 sequences), albeit with a rather low number of occurrences.

In the UO “light dark matter” data set, the DH pathway was still found in three sites but the CBB pathway was only complete in the pool data set; whereas in the UO “dark matter” data set, the CBB and DH pathways were both only complete in the pool data set.

## Discussion

Ultrasmall prokaryotes have only recently been discovered, but what is known to date about their physiology highlights their uniqueness. Members of the CPR and DPANN superphyla from aquifers have recently been described as able to perform reactions related to carbon fixation, although they are usually described as degraders rather than carbon fixing (Castelle et al. 2015, 2018; Anantharaman et al. 2016). Although aquifers represent a fraction of the aquatic environments on Earth, oceans represent a different and larger type of aquatic environment; therefore, the conclusions obtained from studying aquifers may not be applicable to oceans. In particular, we postulate that a broader diversity of microbes, including ultrasmall ones, would thrive in the oceans (although the ultrasmall size fraction has not been extensively studied). This reasoning is in agreement with our hypothesis that new, unidentified lineages of ultrasmall prokaryotes may play a role in autotrophic carbon fixation in the oceans. Using the broad TARA oceans data set, the aim of this work was to determine if members of the CPR and DPANN superphyla, and potentially additional ultrasmall prokaryotes, could contribute to (and eventually complete) pathways of carbon fixation in the oceans.

The diversity of nano-organisms is probably still under appreciated because few studies (Brown et al. 2015; Castelle et al. 2015; Luef et al. 2015; Anantharaman et al. 2016; Paul et al. 2017) have focused on the ultrasmall size fraction of publicly available metagenomes. In our study, for example, analyses of ribosomal markers suggested the existence of at least one large clade of tiny archaea, restricted to two sites that were geographically close in the Indian Ocean (TARA sampling sites 037 MES, 038 MES, and 039 MES). The phylogenetic analyses also hinted at a diversity of novel minute bacteria. Unraveling these additional actors suggests that the ecological and evolutionary roles of microbial diversity within the ocean remain to be fully described. In particular, nano-organisms could deeply impact carbon cycling and carbon fixation; while also contributing to trophic chains and the dynamics of microbial communities (Morris et al. 2012; Biller et al. 2015; Ponomarova and Patil 2015; Zelezniak et al. 2015) in ways that are still to be modeled. Abundant, ubiquitous taxa, such as *Prochlorococcus* and *SAR11* (Partensky et al. 1999; Giovannoni 2017), have already been proposed to affect geochemical cycles and biotic communities at a very large (planetary) scale. Populations of less abundant nano-organisms may also have an influence, at a scale which remains to be determined. Rate measurements will be needed (possibly in simple ecosystems) to test this hypothesis.

In this study, we were able to detect genes involved in the six known autotrophic carbon fixation pathways among those unassigned taxa, exclusive to the ultrasmall size fraction of the TARA OCEAN project. In spite of the limited sequencing depth at each site, these pathways were more than 50% complete at some sites. Moreover, in our stringent data sets (WUO) the anaerobic and energetically efficient DH pathway was more than 50% complete at 33 sampling sites. Interestingly, this in contrast to the carbon fixation pathways associated with CPR and DPANN superphyla in aquifers (Probst et al. 2017), which suggest that nano-organisms may have a broader contribution to carbon fixation than currently assumed. It is possible that some carbon fixation genes are carried by viral particles (although our analyses did not find any signal for this).

Assuming microbial communities were sufficiently well sampled, the detection of partial metabolic pathways and associated key enzymes raises the question of the actual contribution of these genes to carbon fixation and cycling in the environment. These genes may play an effective role under two distinct conditions. First, the genomes hosting the partial pathways may also host alternative genes encoding for unknown enzymes that can perform the missing steps for carbon fixation. Second, alternative genes encoding unknown enzymes would perform the missing steps, which may be distributed across phylogenetically diverse community members and interacting *via* metabolic hand-offs (Embree et al. 2015; Tsoi et al. 2018; Rubin-Blum et al. 2019). The contribution of marine nano-organisms to carbon fixation might therefore be a collective property, in which different microbes contribute to different steps of carbon fixation. Such metabolic cooperation in microbial communities has been described (DeLong 2007; Stams and Plugge 2009), but in the ocean such interactions might be rare except for communities associated with floating particles and sediments. Under the first hypothesis, transporters for some of the metabolic intermediates should exist in nature. We indeed found transporter candidates in the WUO “dark matter” data set, including a putative citrate/succinate antiporter (COG0471), both molecules being present in rTCA, and numerous ATPase components of ABC transporters (COG0488). The alternative hypothesis, that is, the contribution of specific novel lineages to carbon fixation, could lead to the discovery of new autotrophic nano-organisms, which are of similar importance to *Prochlorococcus* or SAR11, currently the smallest described carbon fixing organism.

Under both hypotheses, our study encourages single cell genome analyses and/or the binning of metagenomes into genomes of nanosized micro-organisms. This would allow further characterization of the precise mechanisms by which the organisms contribute to carbon fixation.

## Supplementary Material

Supplementary data are available at *Genome Biology and Evolution* online.

## Supplementary Material

Supplement_Material_evz050
