# Supplementary material for: Carbon Fixation by Marine Ultrasmall Prokaryotes
Source: Genome Biol Evol. 2019 Mar 23;11(4):1166–77. doi: 10.1093/gbe/evz050 (PMC6475129; doi:10.1093/gbe/evz050)
Supplement: Supplement_Material_evz050 [file supplement_material_evz050.zip › SI_Fig1.pdf]

Color chart

- all
- UO
- WUO
- CPR
- DPANN

- Acidobacteria
- Actinobacteria
- Aquificae
- Armatimonadetes
- Bacteroidetes
- Caldiserica
- Calditrichaeota
- Chlamydiae
- Chlorobi
- Chloroflexi
- Chrysiogenetes
- Coprothermobacterota

- Cyanobacteria
- Deferribacteres
- Dictyoglomi
- Elusimicrobia
- Fibrobacteres
- Firmicutes
- Fusobacteria
- Gemmatimonadetes
- Ignavibacteriae
- Kiritimatiellaeota
- Melainabacteria
- NC10
- Nitrospirae
- Planctomycetes
- Proteobacteria
- Spirochaetes
- Synergistetes
- Tenericutes
- Thermodesulfobacteria
- Thermotogae
- Thermus
- Verrucomicrobia

- Annelida
- Apicomplexa
- Arthropoda
- Ascomycota
- Bacillariophyta
- Basidiomycota
- Chlorophyta
- Chordata
- Cnidaria
- Eukaryota
- Microsporidia
- Mollusca
- Nematoda
- Placozoa
- Platyhelminthes
- Streptophyta

- Bathyarchaeota
- Crenarchaeota
- Euryarchaeota
- Korarchaeota
- Lokiarchaeota
- Thaumarchaeota

K02863 (large subunit ribosomal protein L1)
K02865 (large subunit ribosomal protein L10Ae)

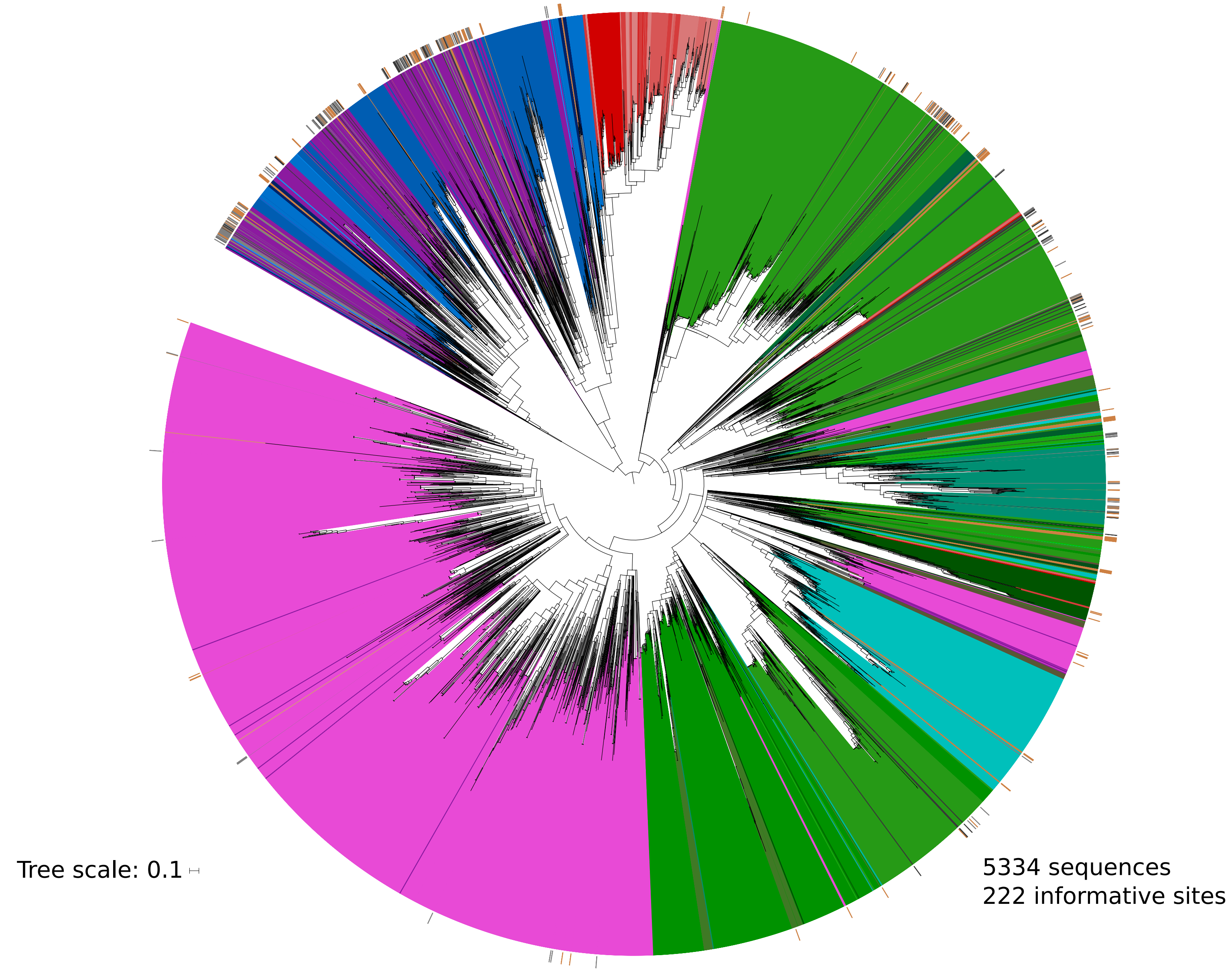

K02864 (large subunit ribosomal protein L10)
K02941 (large subunit ribosomal protein LP0)

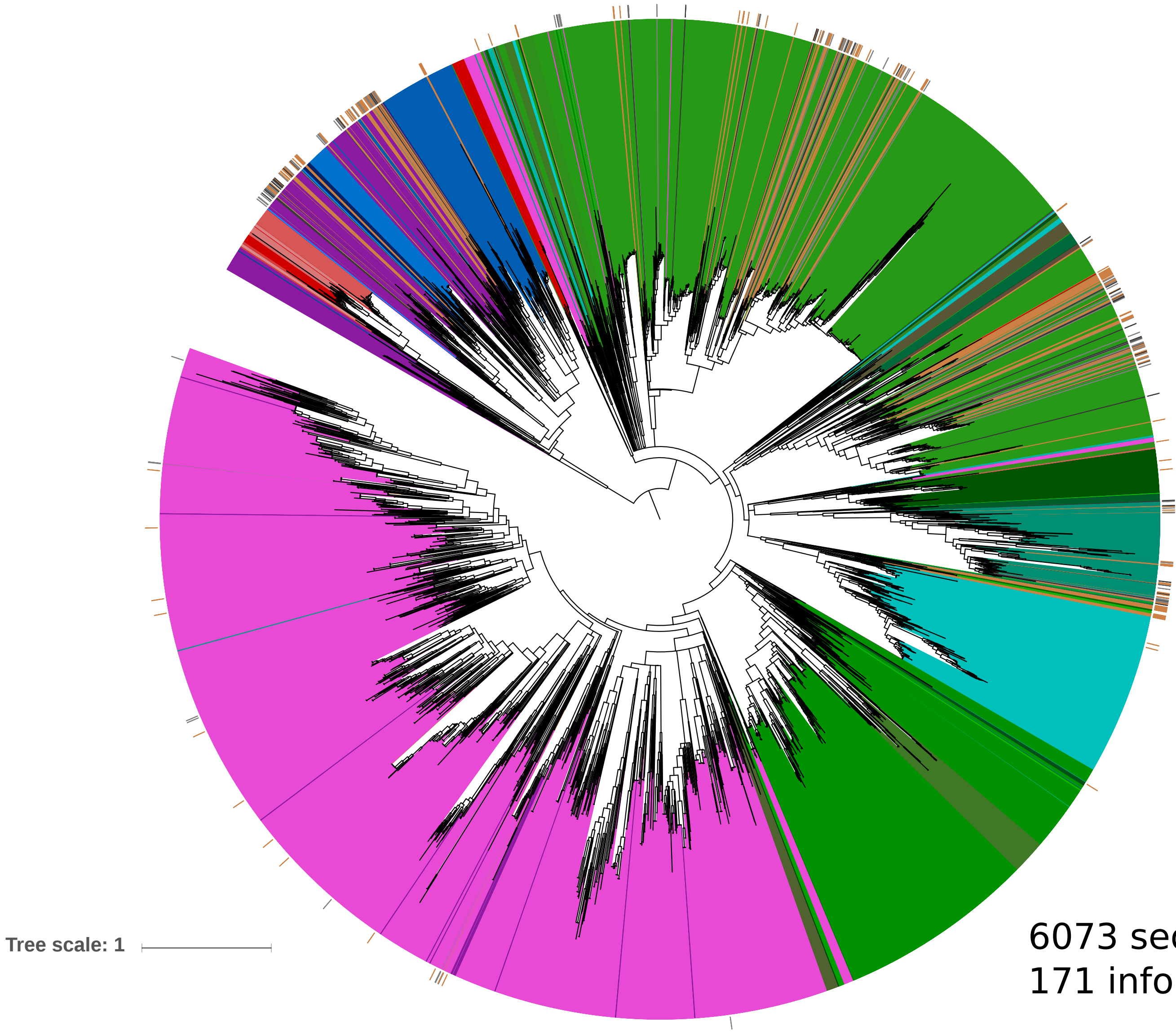

6073 sequences
171 informative sites

large subunit ribosomal protein L11 (K02867)
large subunit ribosomal protein L12e (K02870)

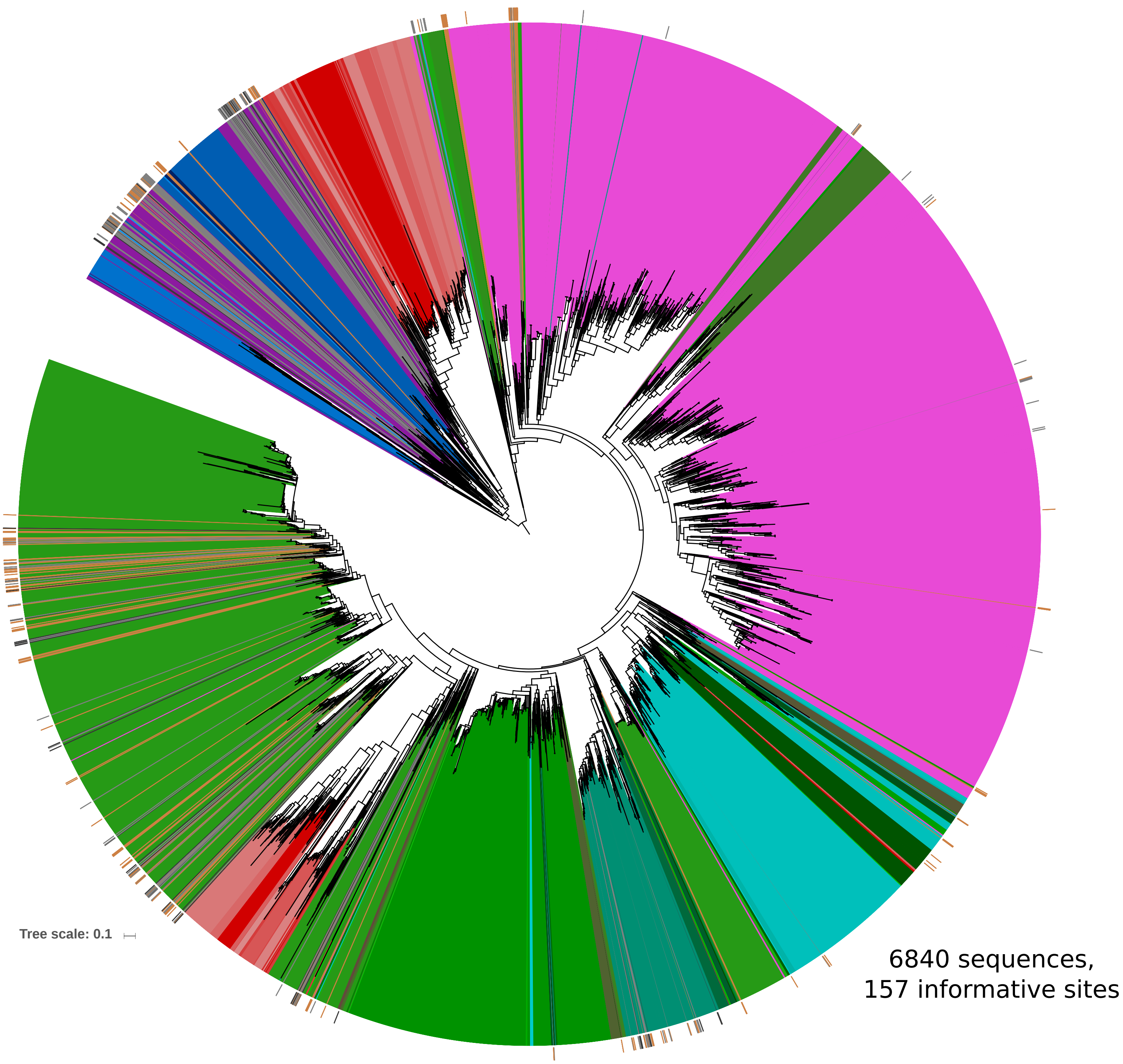

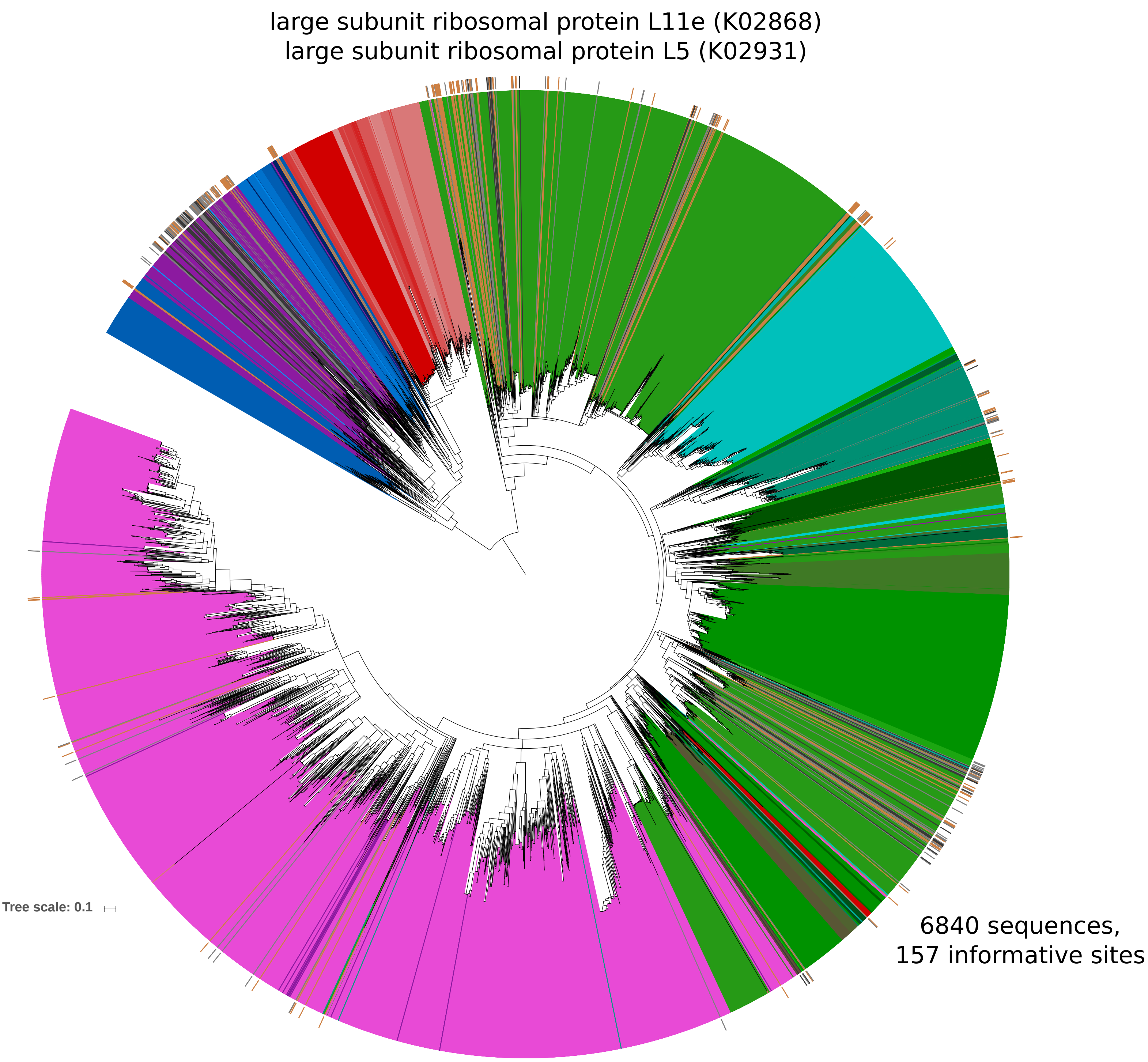

large subunit ribosomal protein L11e (K02868)
large subunit ribosomal protein L5 (K02931)
6840 sequences,
157 informative sites
Tree scale: 0.1

large subunit ribosomal protein L13 (K02871)
large subunit ribosomal protein L13Ae (K02872)

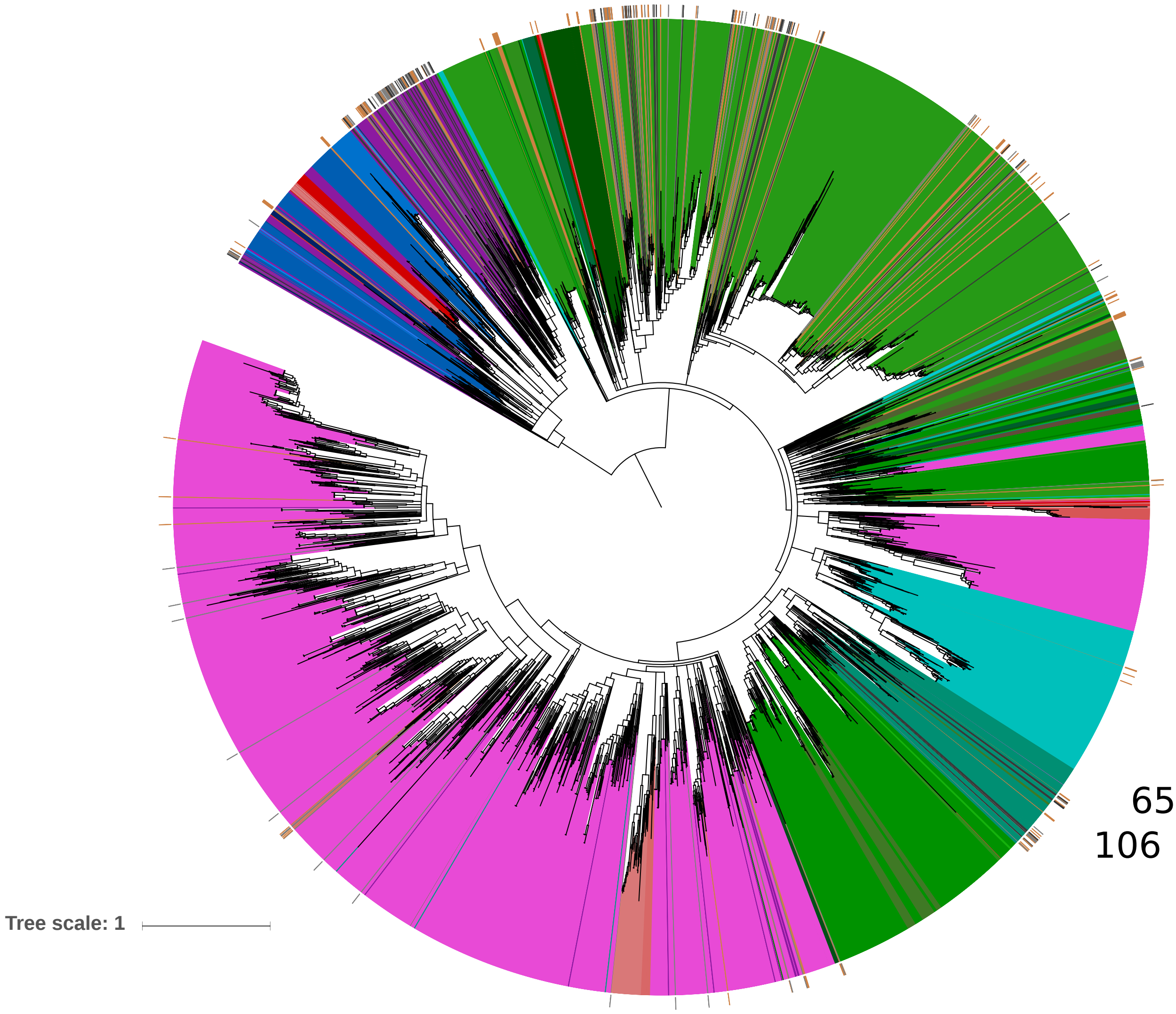

6533 sequences,
106 informative sites

large subunit ribosomal protein L14 (K02874)
large subunit ribosomal protein L23e (K02894)

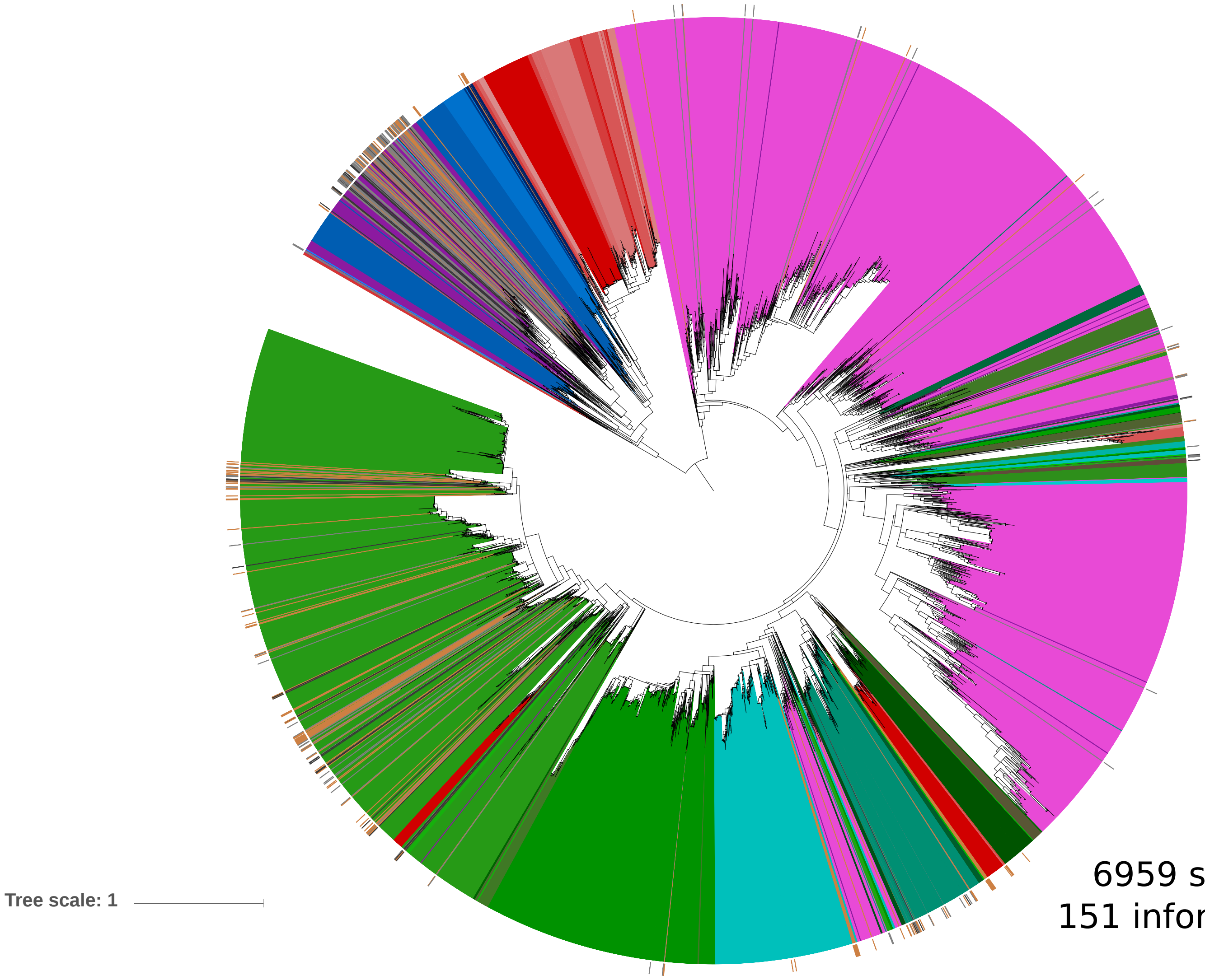

6959 sequences,
151 informative sites

large subunit ribosomal protein L14e (K02875)

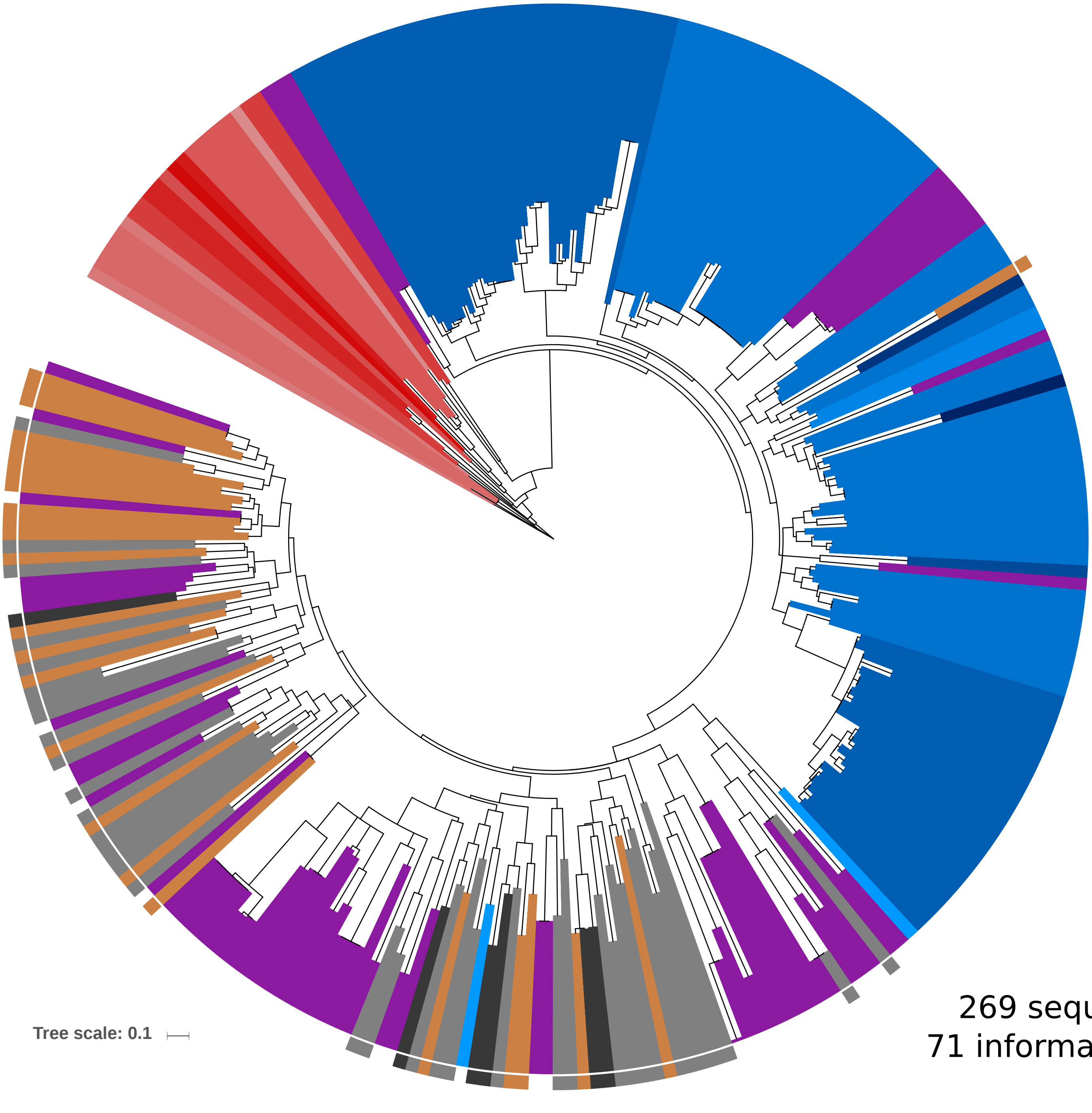

269 sequences,
71 informative sites

large subunit ribosomal protein L15 (K02876)
large subunit ribosomal protein L18e (K02883)
large subunit ribosomal protein L27Ae (K02900)

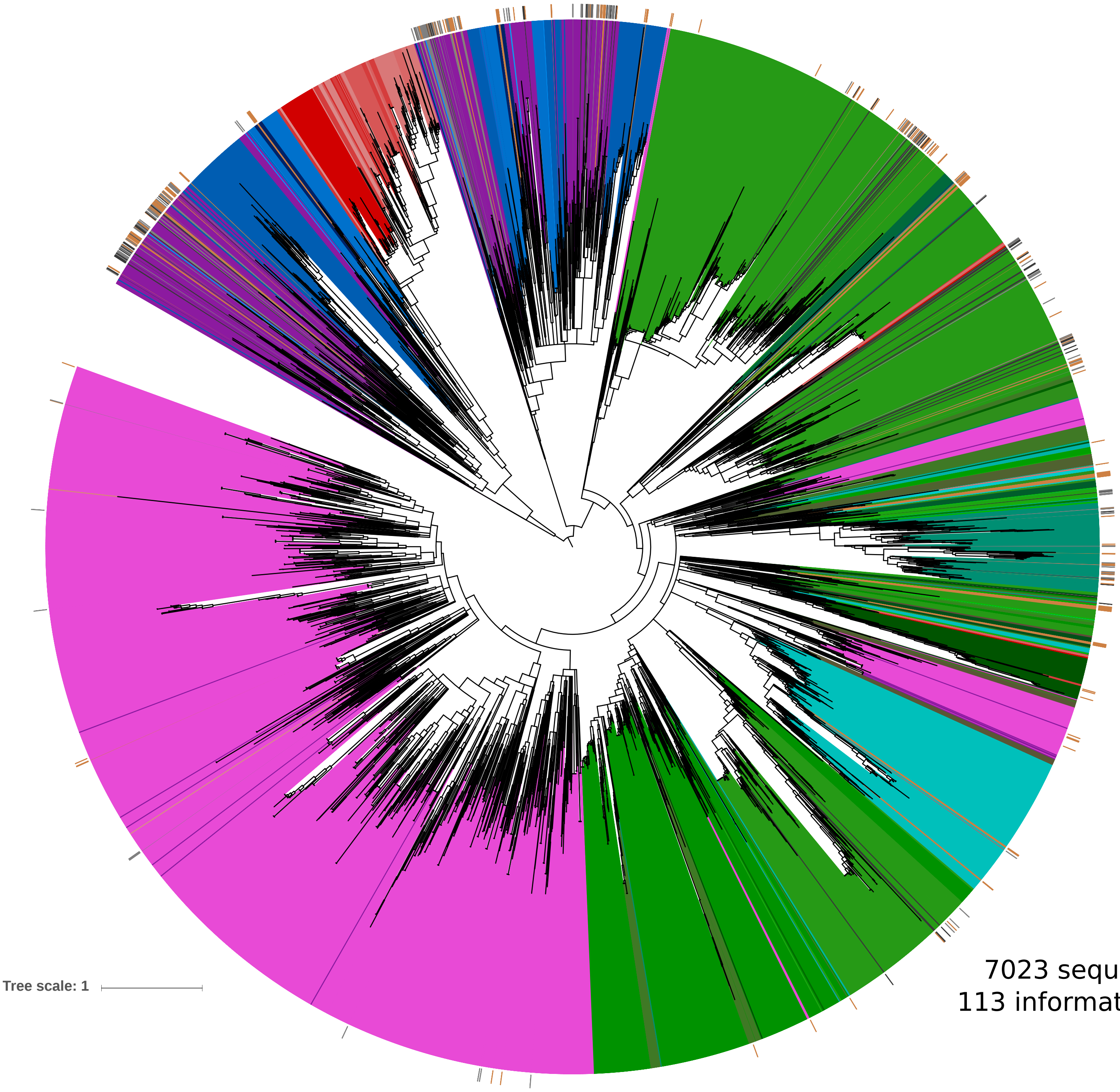

7023 sequences,
113 informative sites

Large Subunit Ribosomal Protein L15e (K02877)

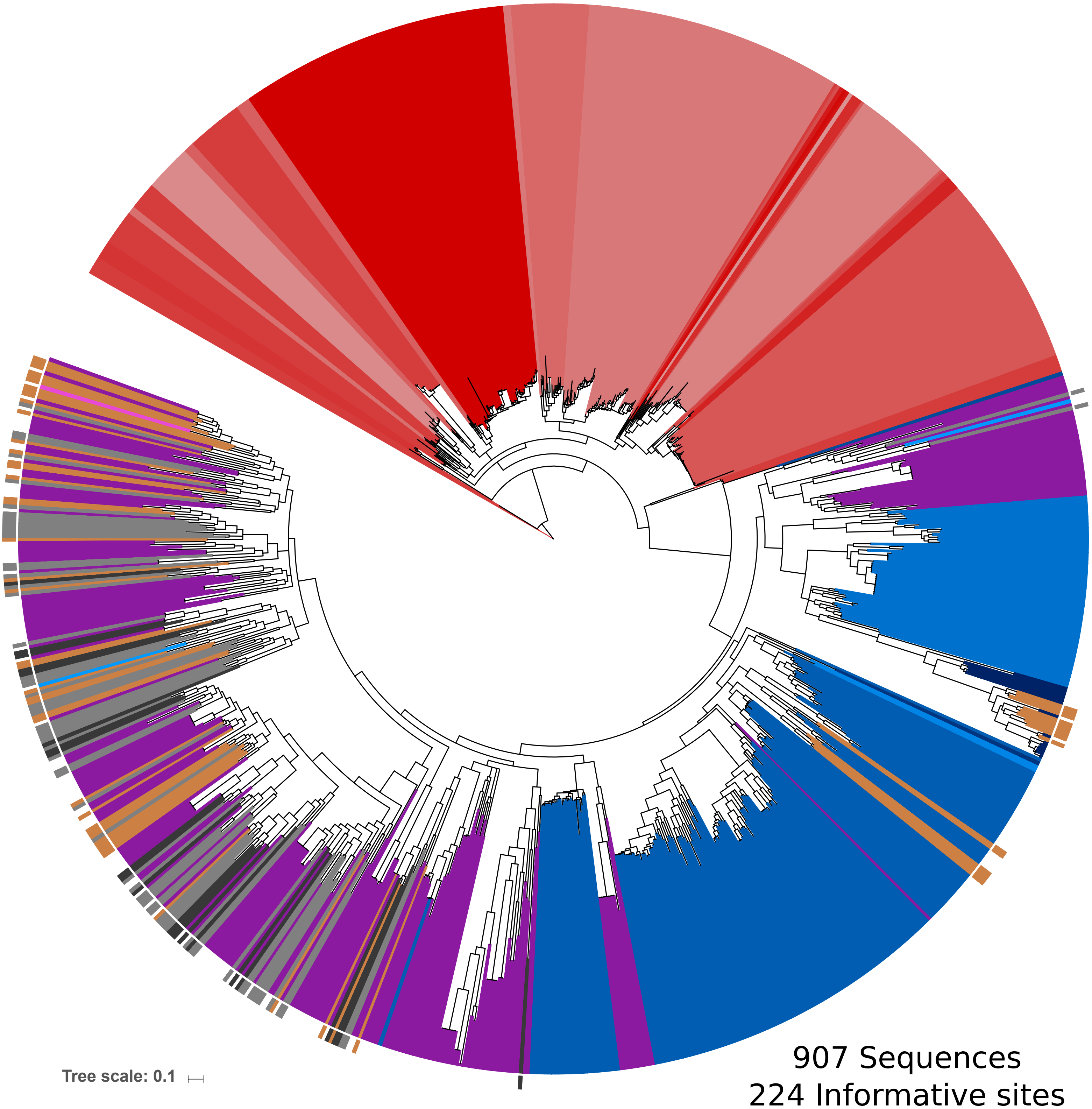

907 Sequences
224 Informative sites

Large Subunit Ribosomal Protein L17 (K02879)

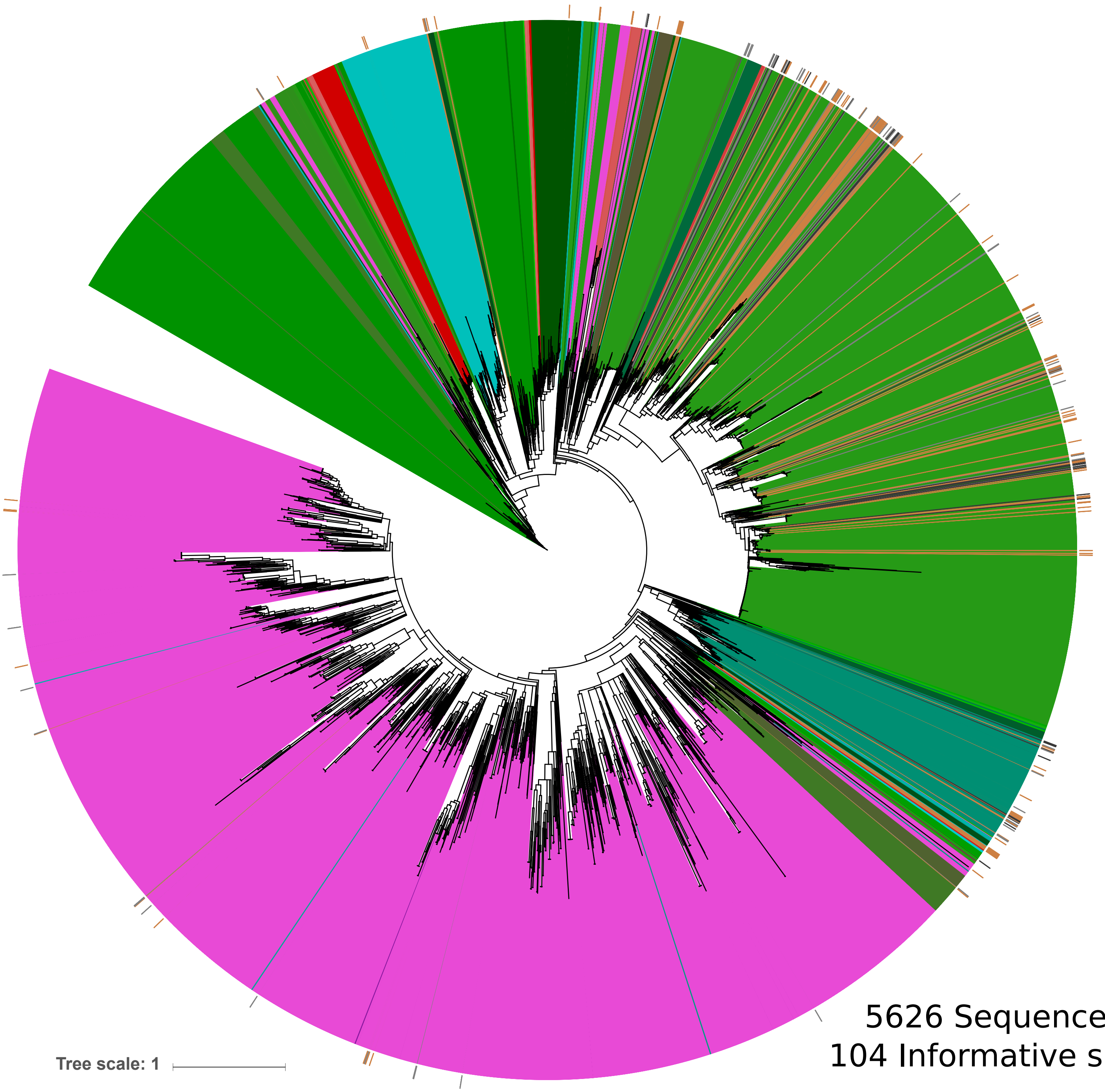

5626 Sequences
104 Informative sites

Large Subunit Ribosomal Protein L17e (K02880)/Large Subunit Ribosomal Protein L22 K02890

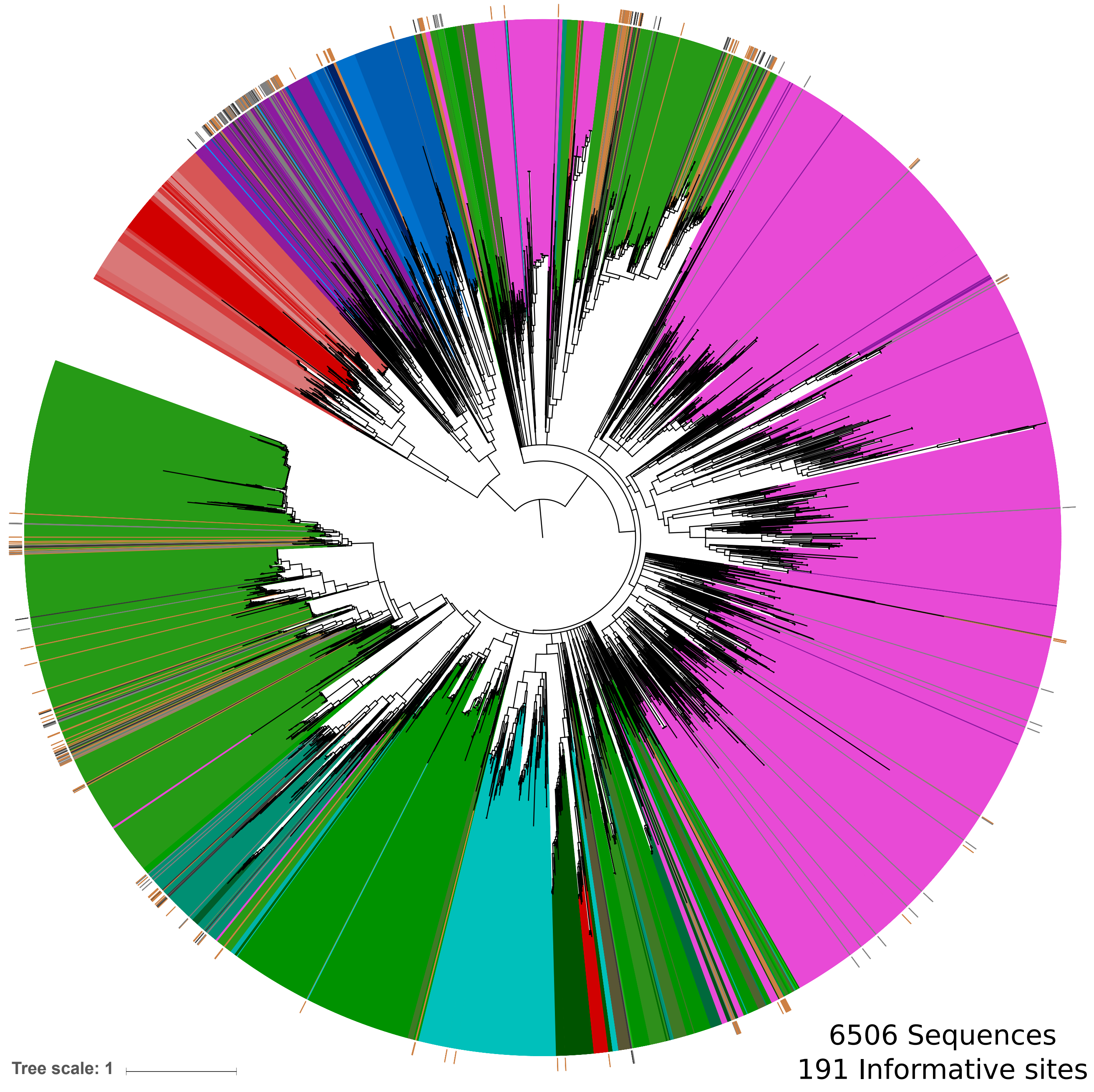

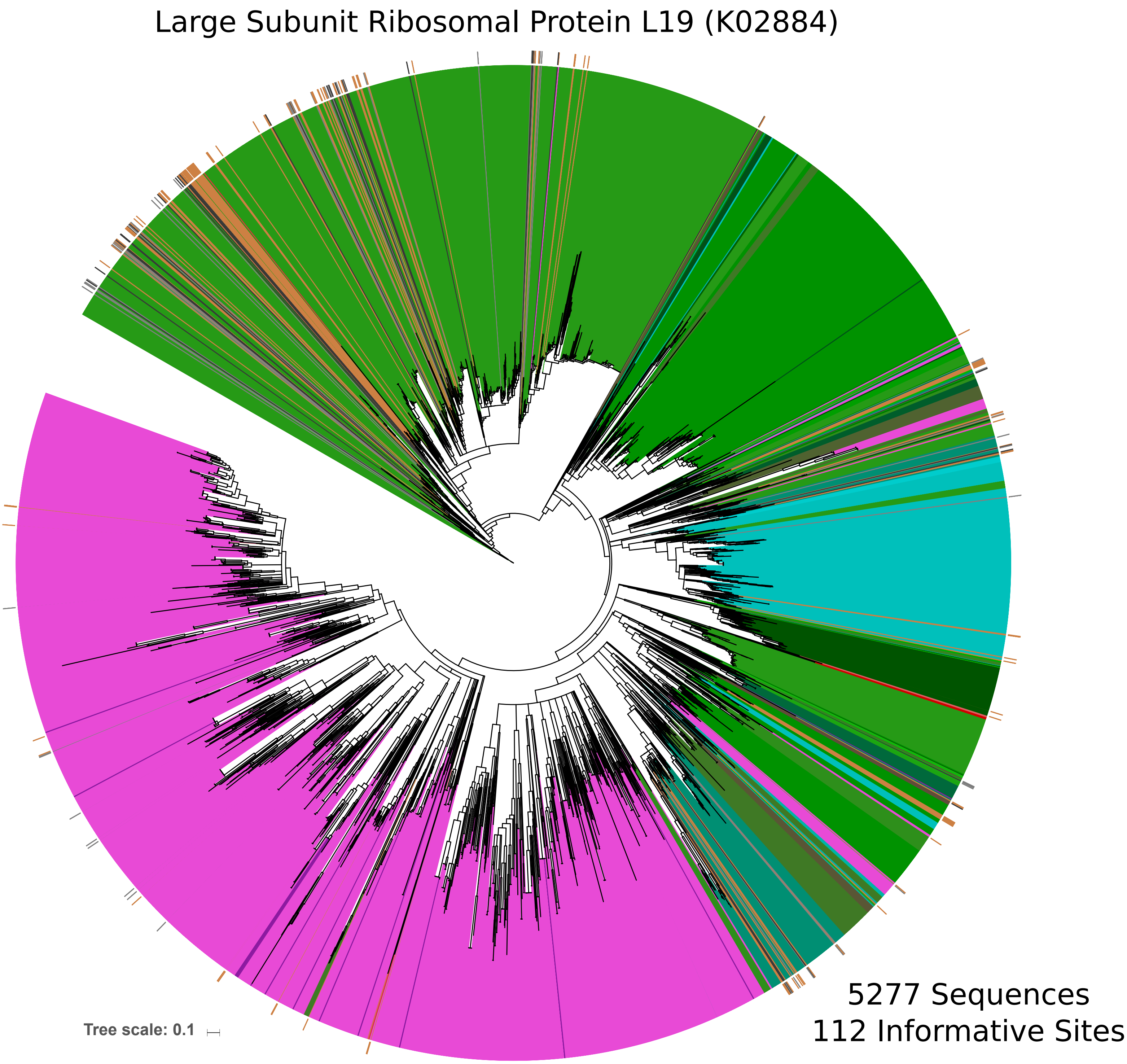

Large Subunit Ribosomal Protein L19 (K02884)
Tree scale: 0.1
5277 Sequences
112 Informative Sites

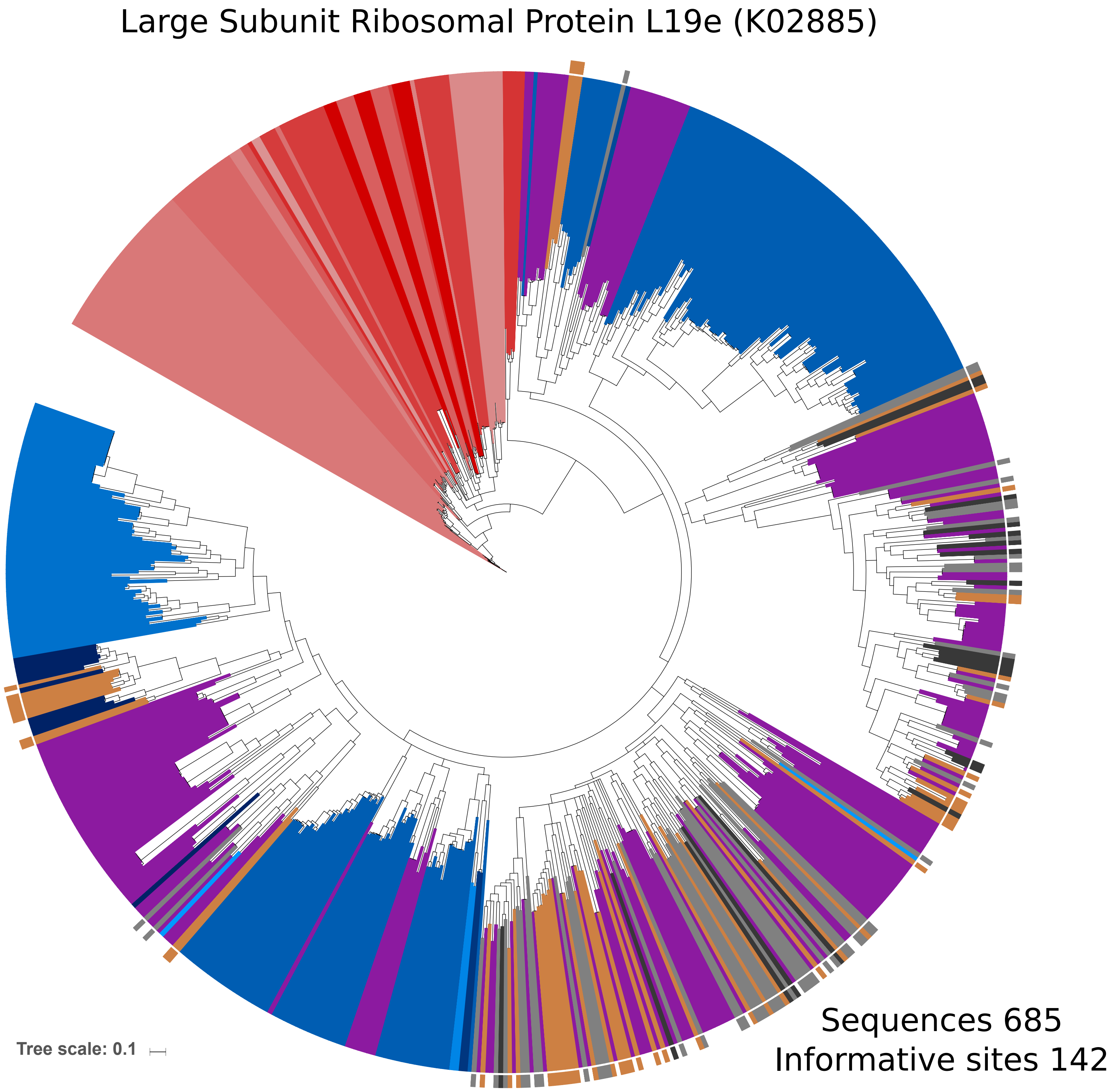
Large Subunit Ribosomal Protein L19e (K02885)
Sequences 685
Informative sites 142
Tree scale: 0.1

Large Subunit Ribosomal Protein L2 (K02886)
Large Subunit Ribosomal Protein L8e (K02938)

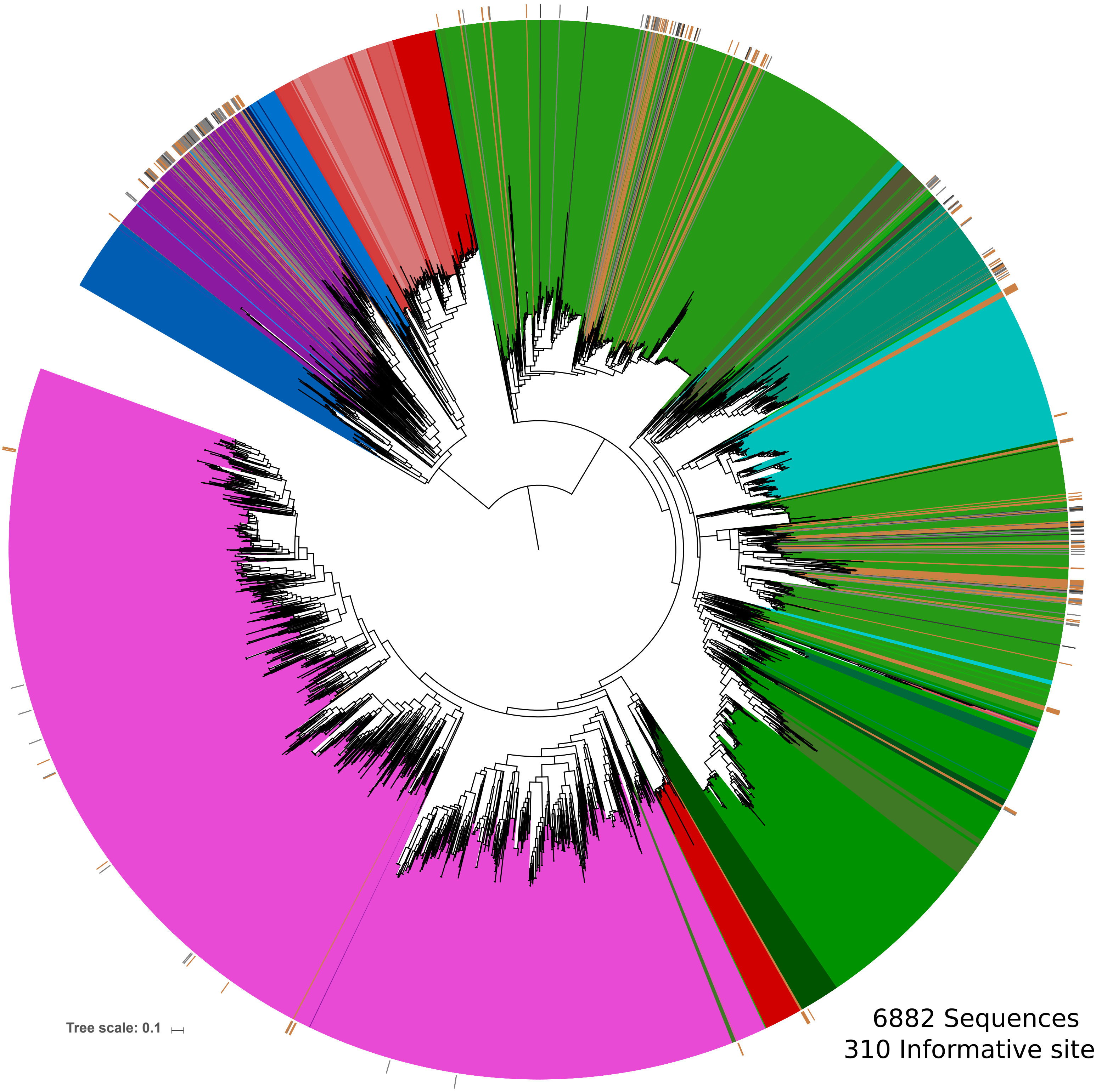

6882 Sequences
310 Informative sites

Large Subunit Ribosomal Protein L20 (K02887)

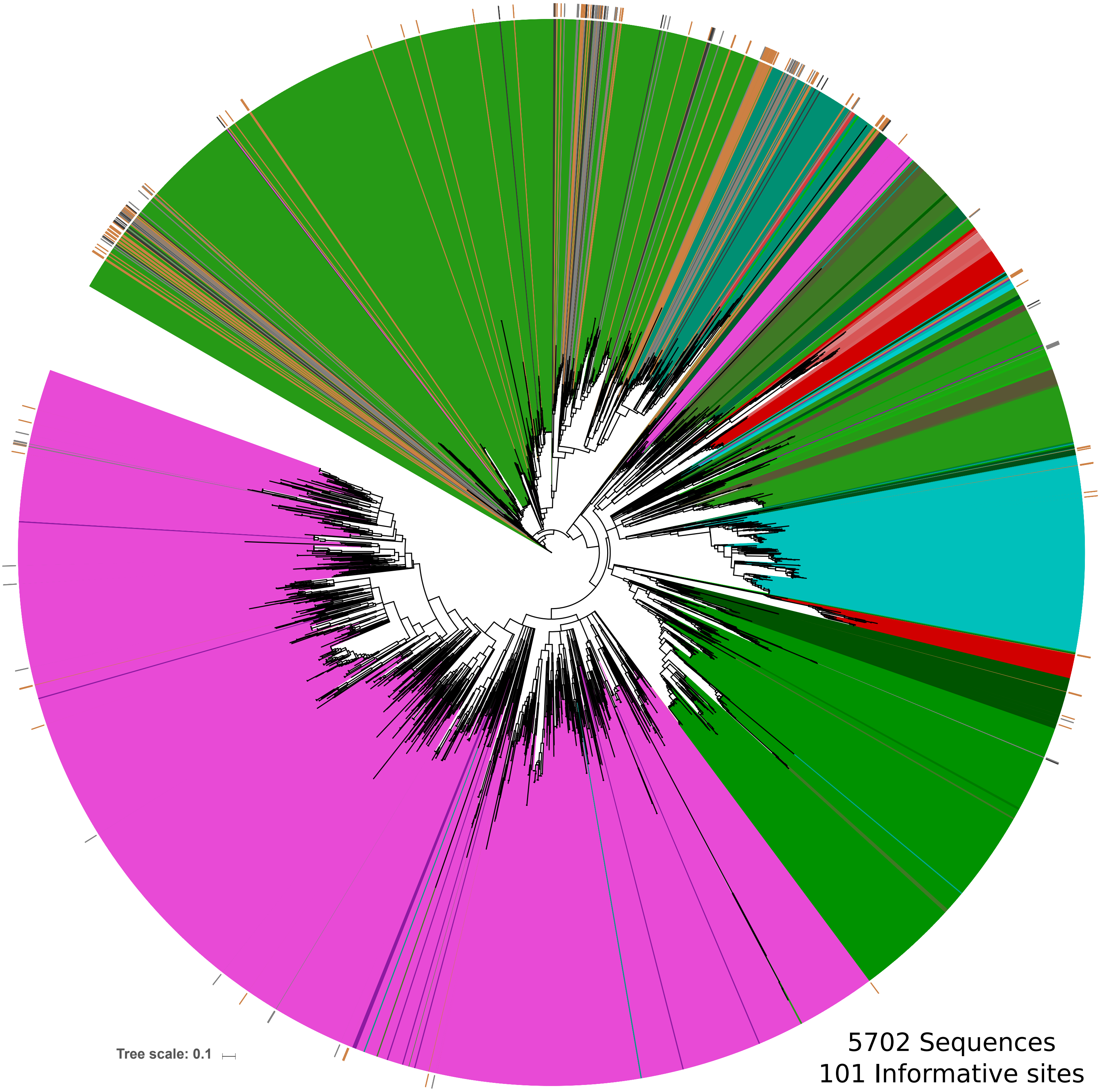

5702 Sequences
101 Informative sites

Large Subunit Ribosomal Protein L21 (K02888)
Large Subunit Ribosomal Protein L27 (K02899)

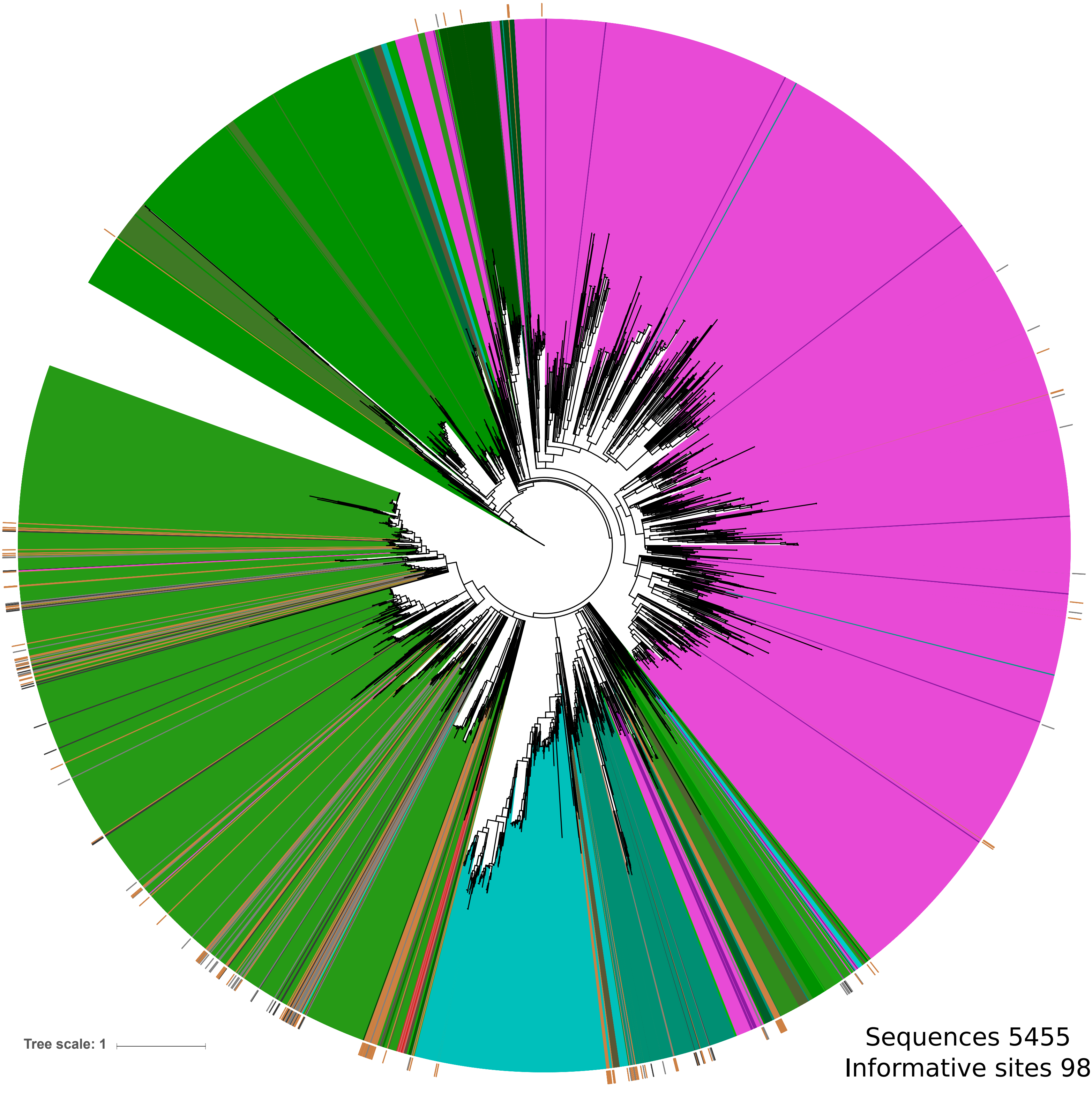

Sequences 5455
Informative sites 98

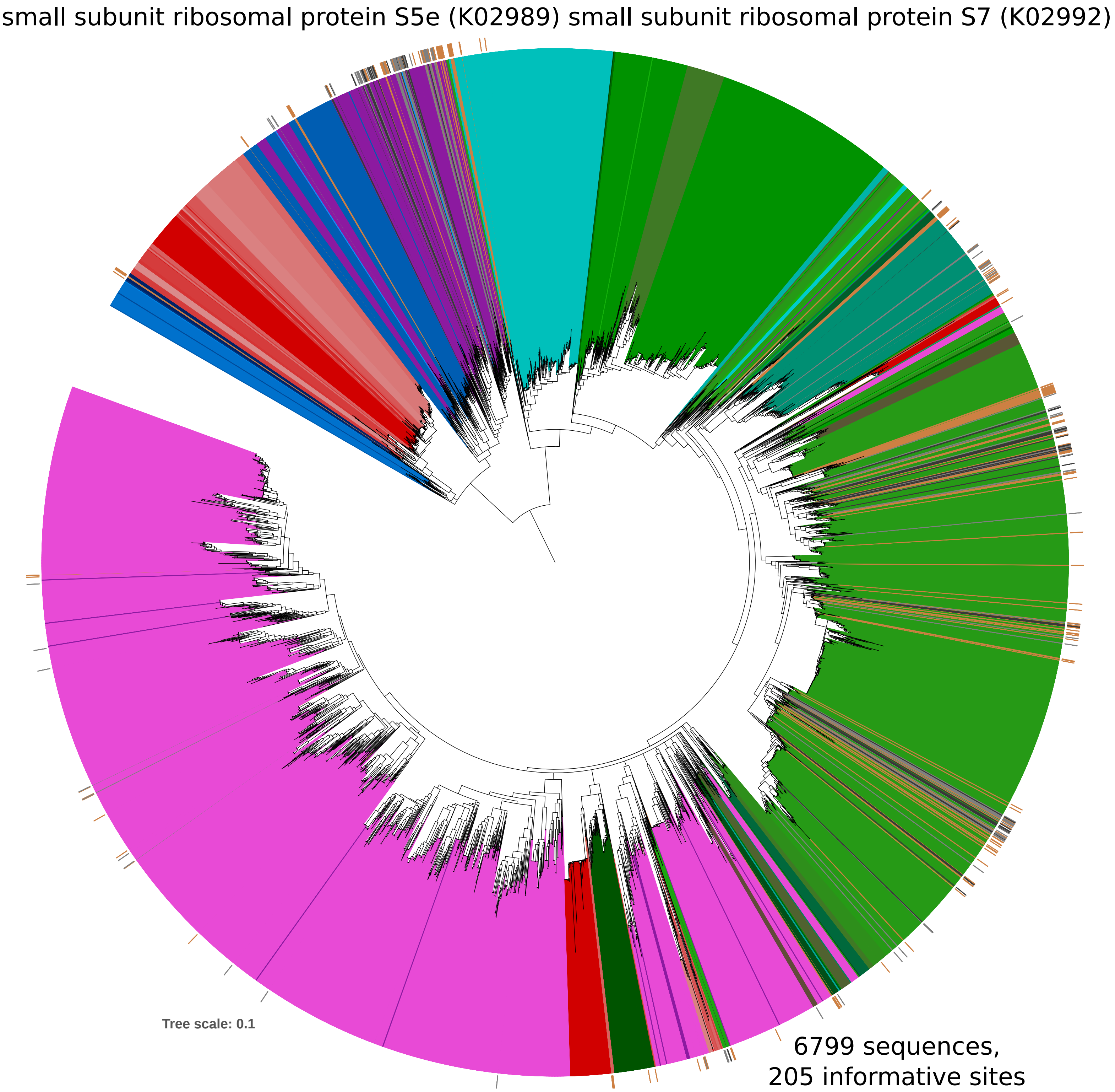
small subunit ribosomal protein S5e (K02989) small subunit ribosomal protein S7 (K02992)
Tree scale: 0.1
6799 sequences,
205 informative sites

Large Subunit Ribosomal Protein L21e (K02889)

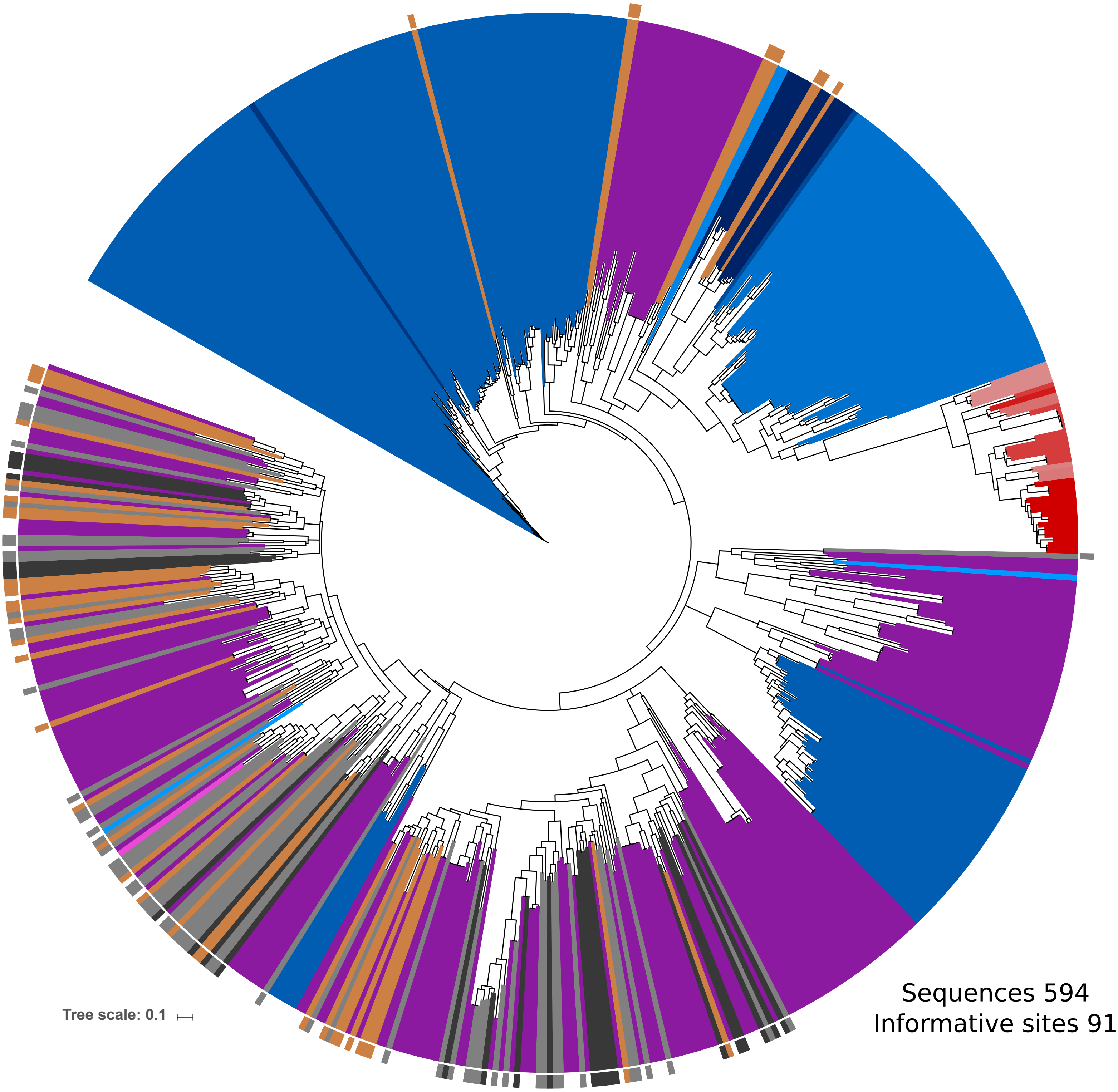

Large Subunit Ribosomal Protein L23 (K02892)
Large Subunit Ribosomal Protein L23Ae (K02893)

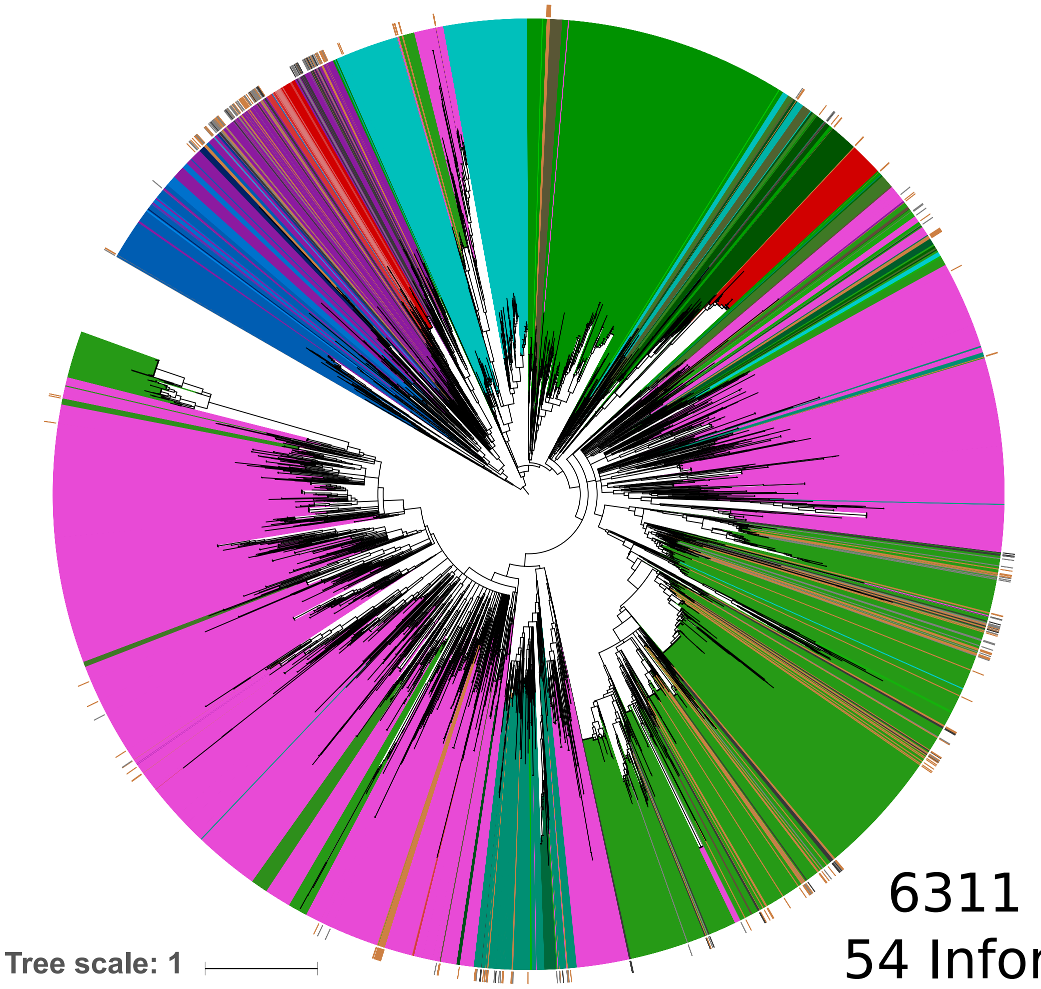

6311 Sequences
54 Informative sites

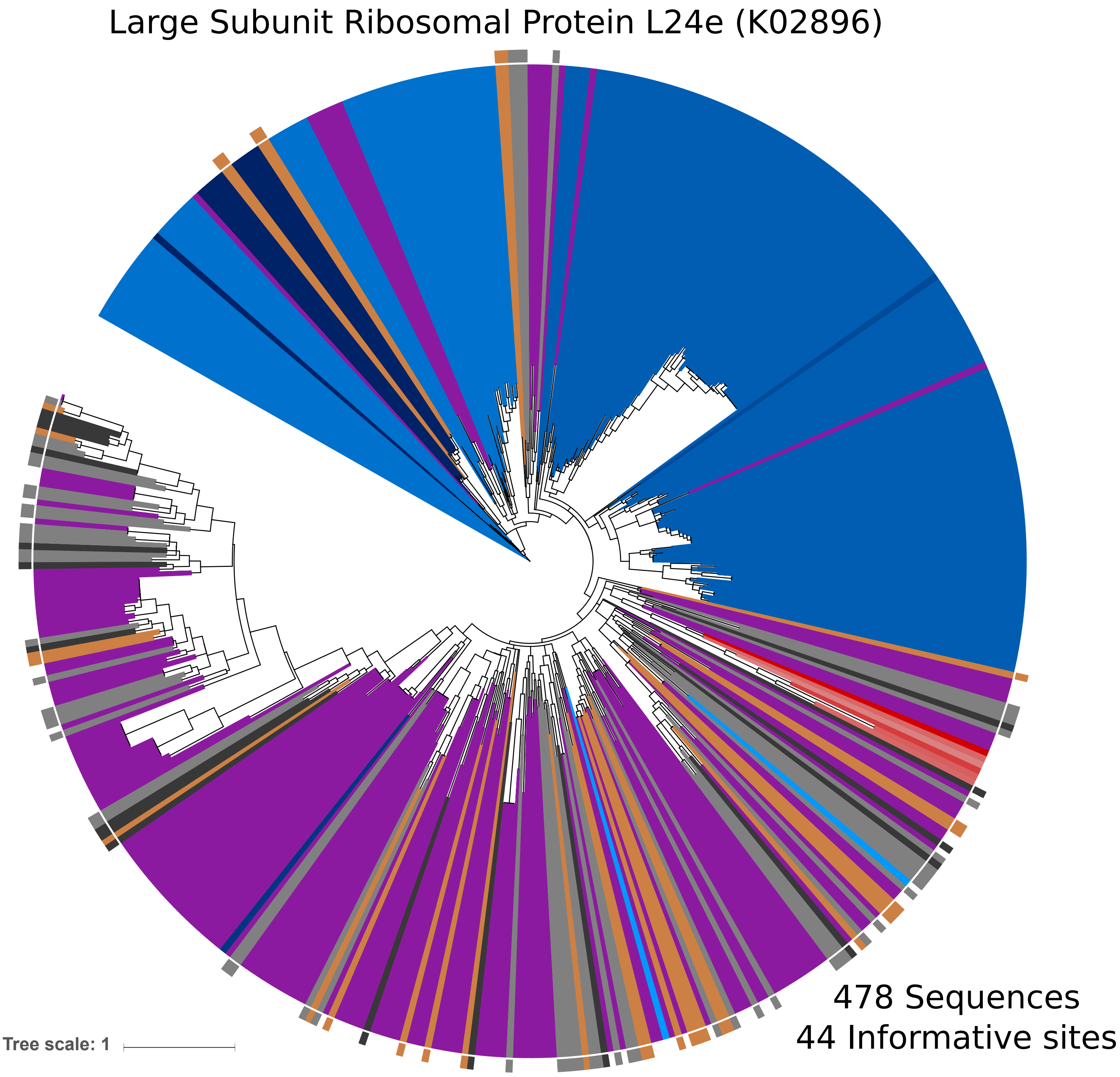
Large Subunit Ribosomal Protein L24e (K02896)
478 Sequences
44 Informative sites
Tree scale: 1

large subunit ribosomal protein L25 (K02897)

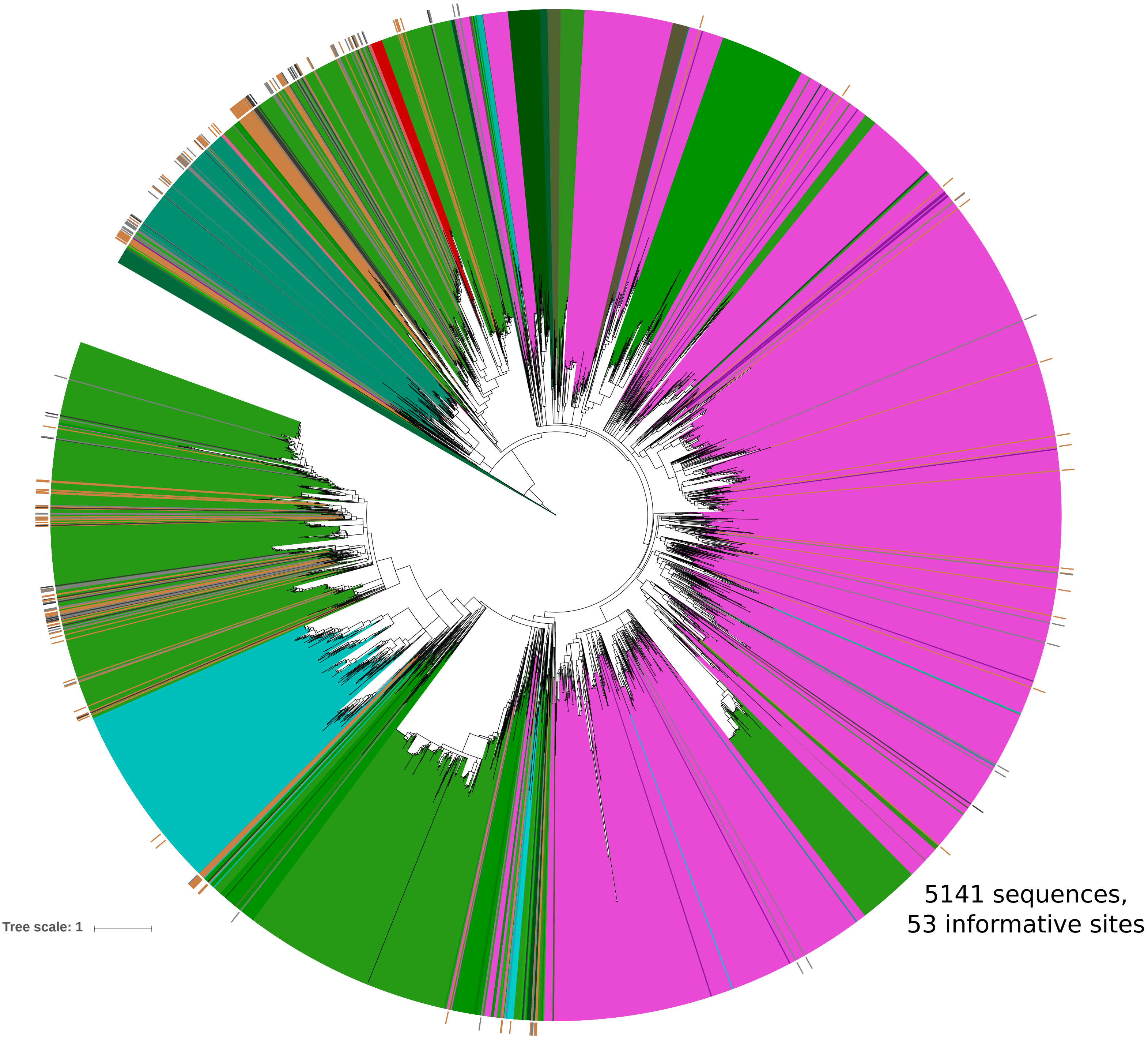

large subunit ribosomal protein L27 (K02899)

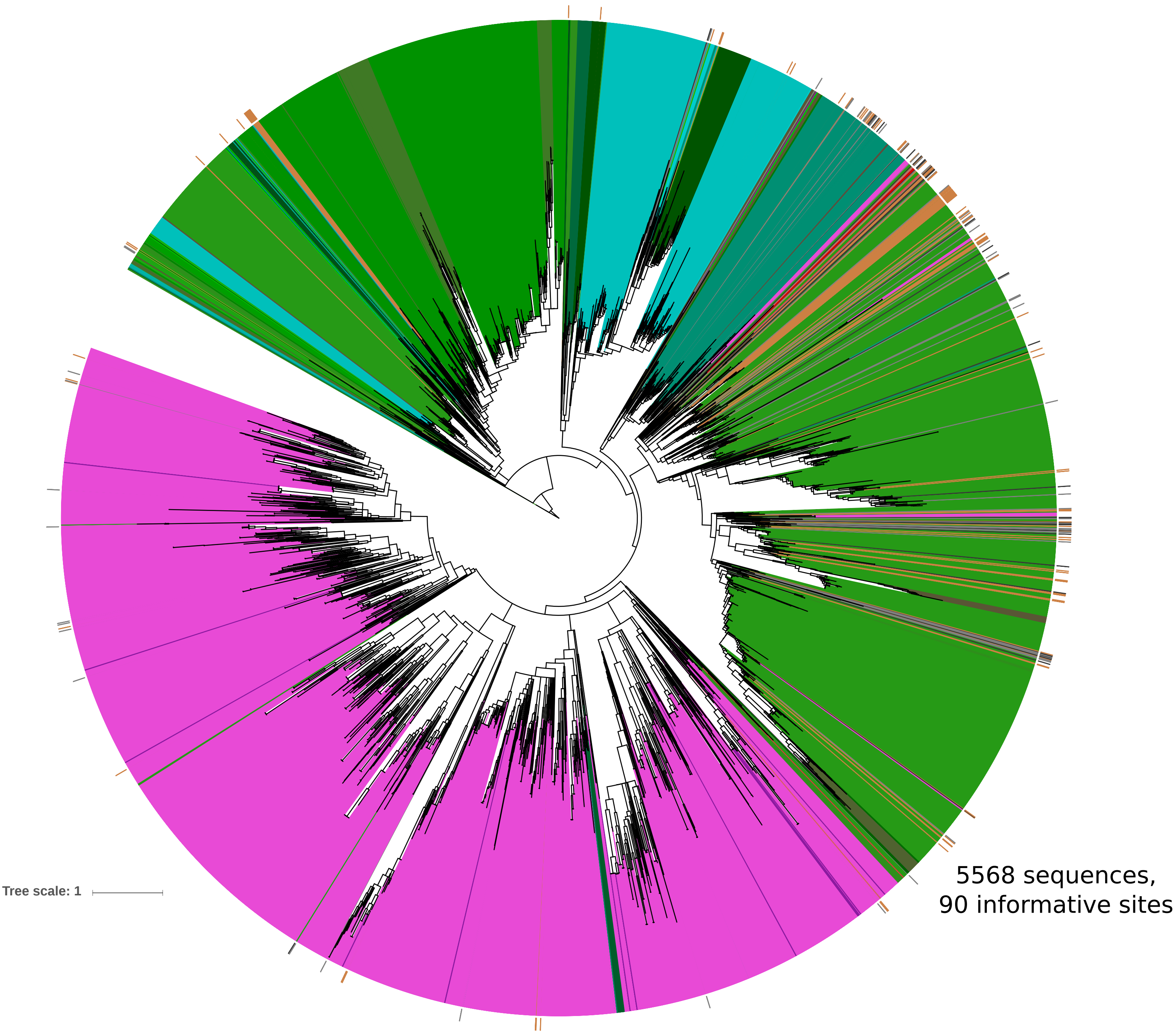

large subunit ribosomal protein L29 (K02904)

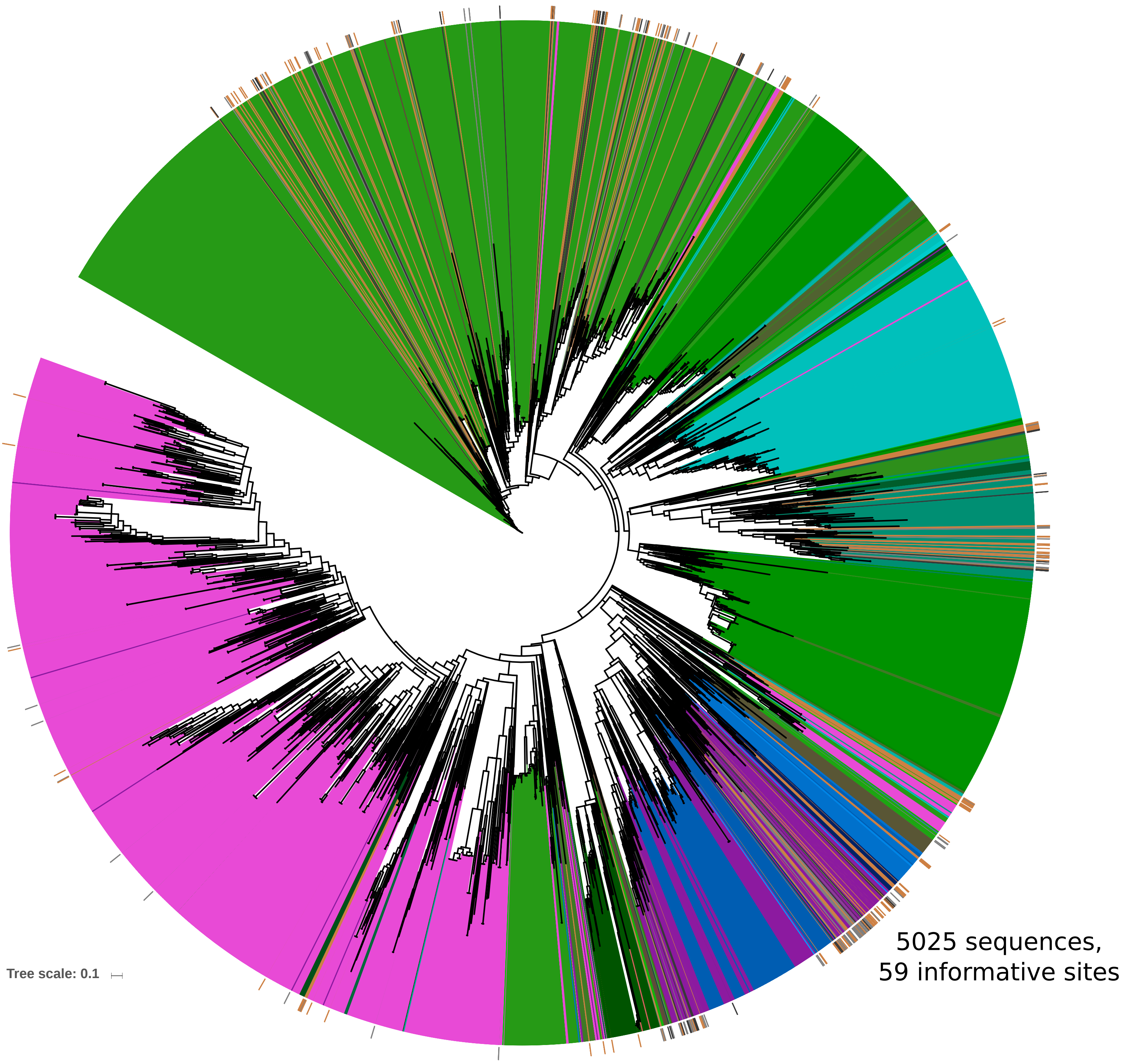

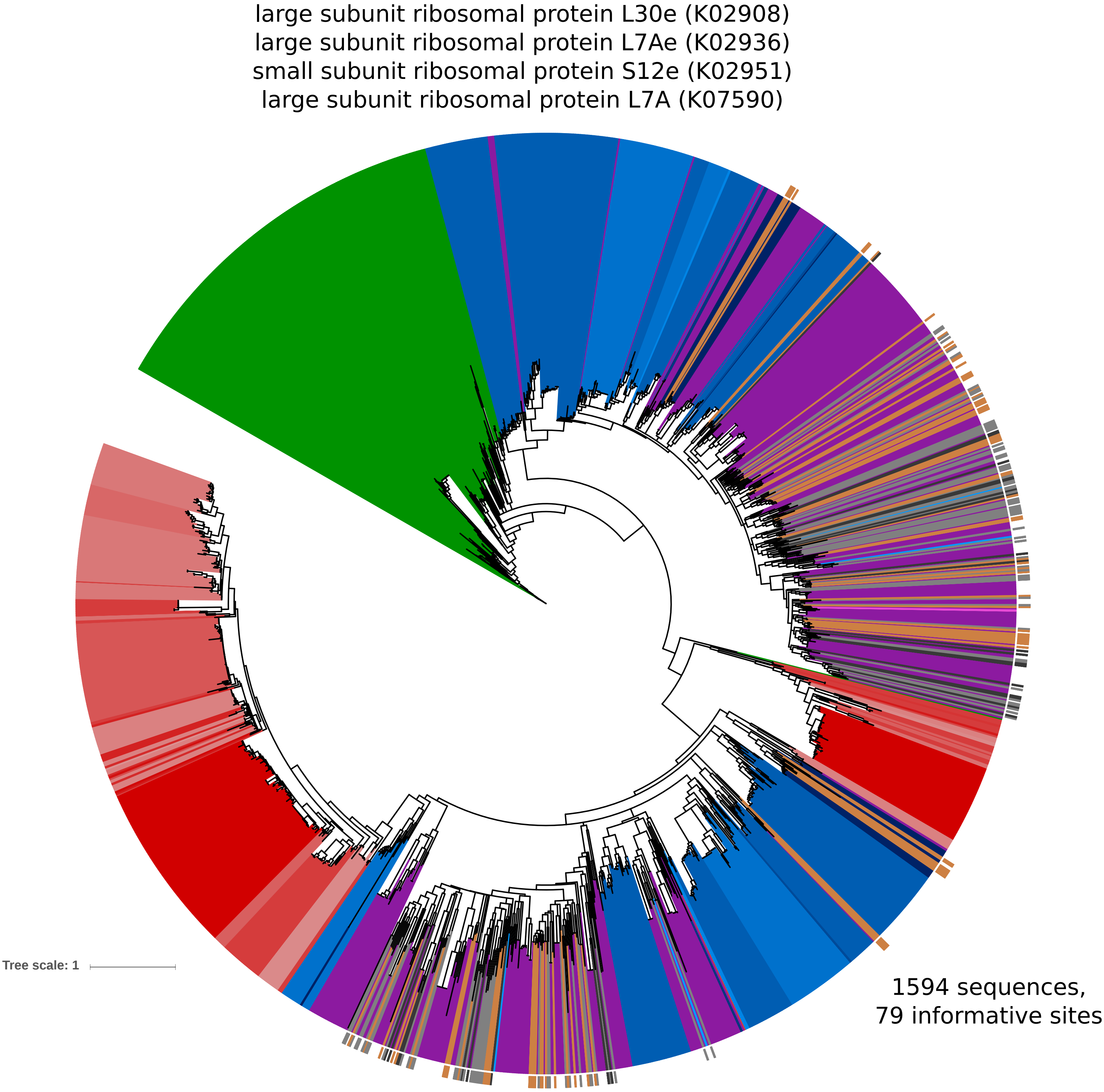

large subunit ribosomal protein L30e (K02908)
large subunit ribosomal protein L7Ae (K02936)
small subunit ribosomal protein S12e (K02951)
large subunit ribosomal protein L7A (K07590)
Tree scale: 1
1594 sequences,
79 informative sites

large subunit ribosomal protein L31 (K02909)

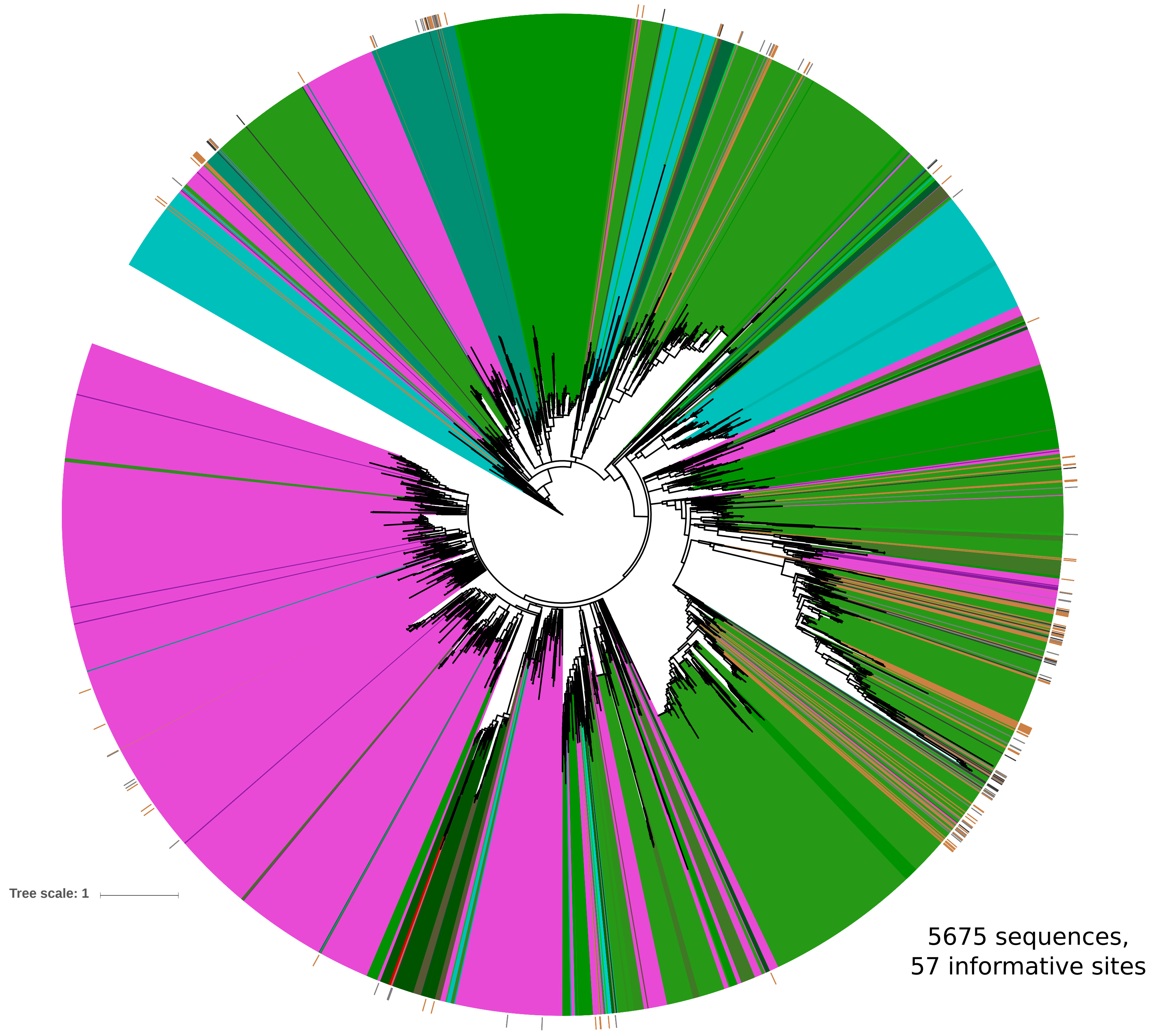

large subunit ribosomal protein L31e (K02910)

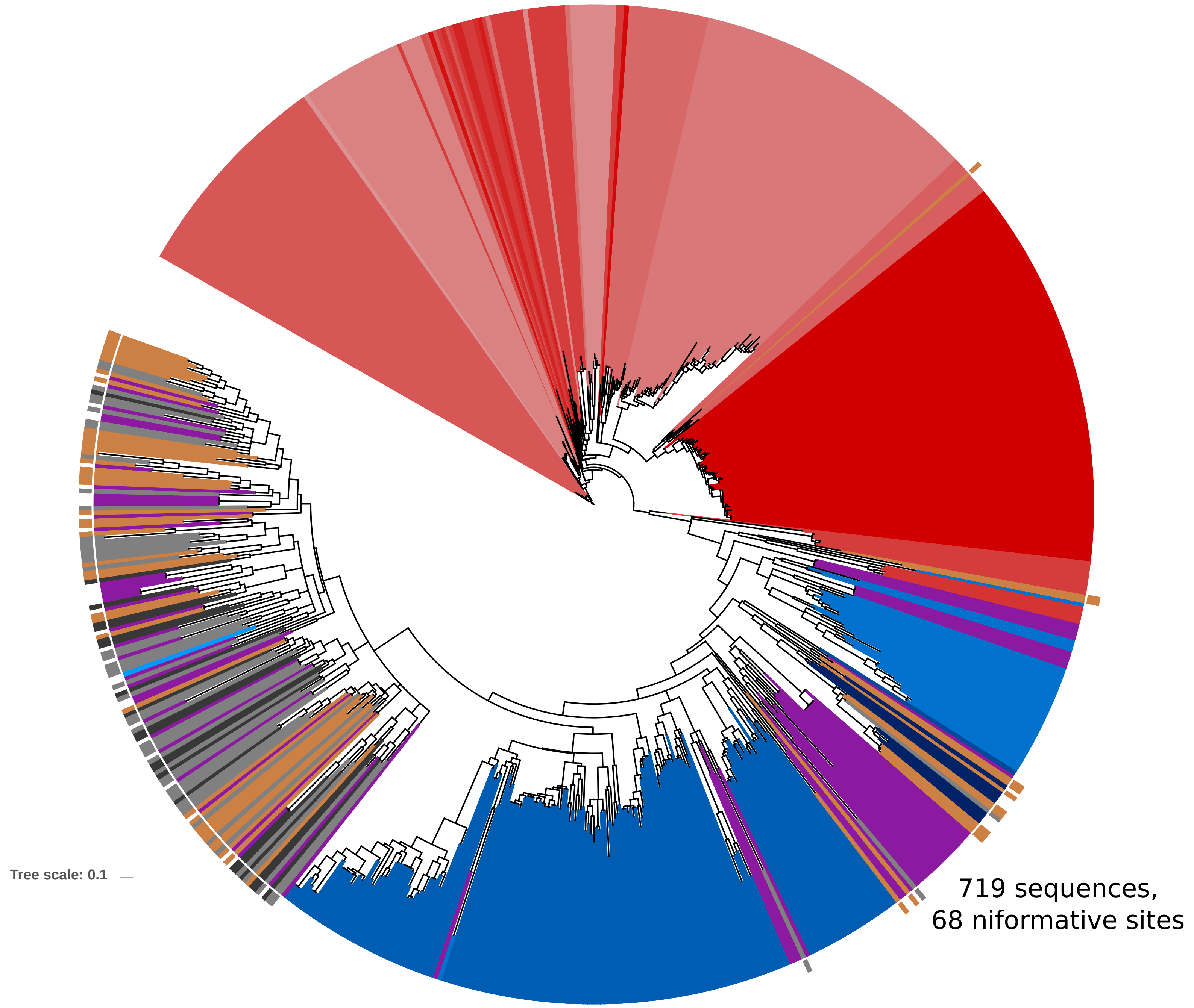

large subunit ribosomal protein L32 (K02911)

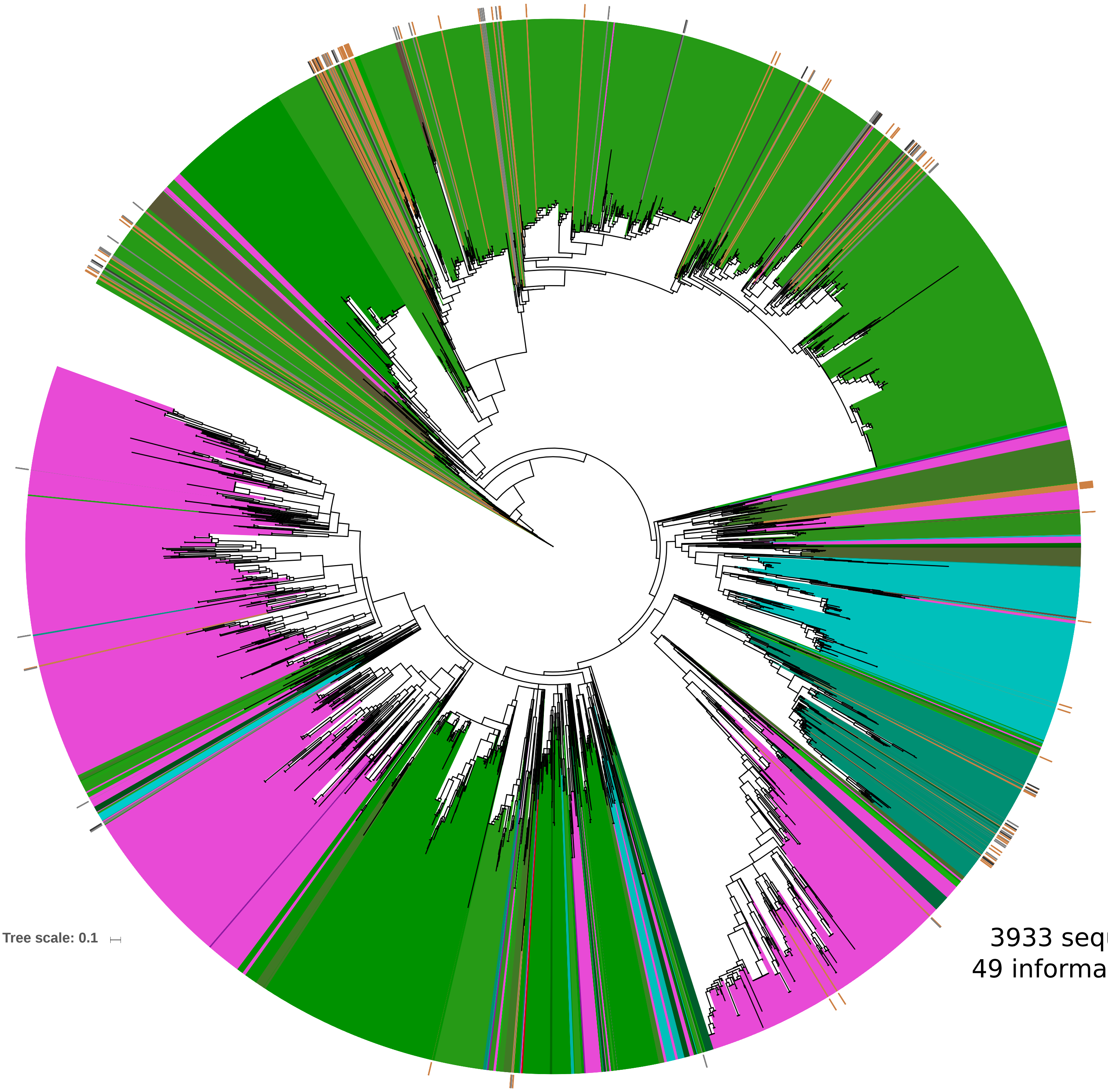

3933 sequences,
49 informative sites

large subunit ribosomal protein L32e (K02912)

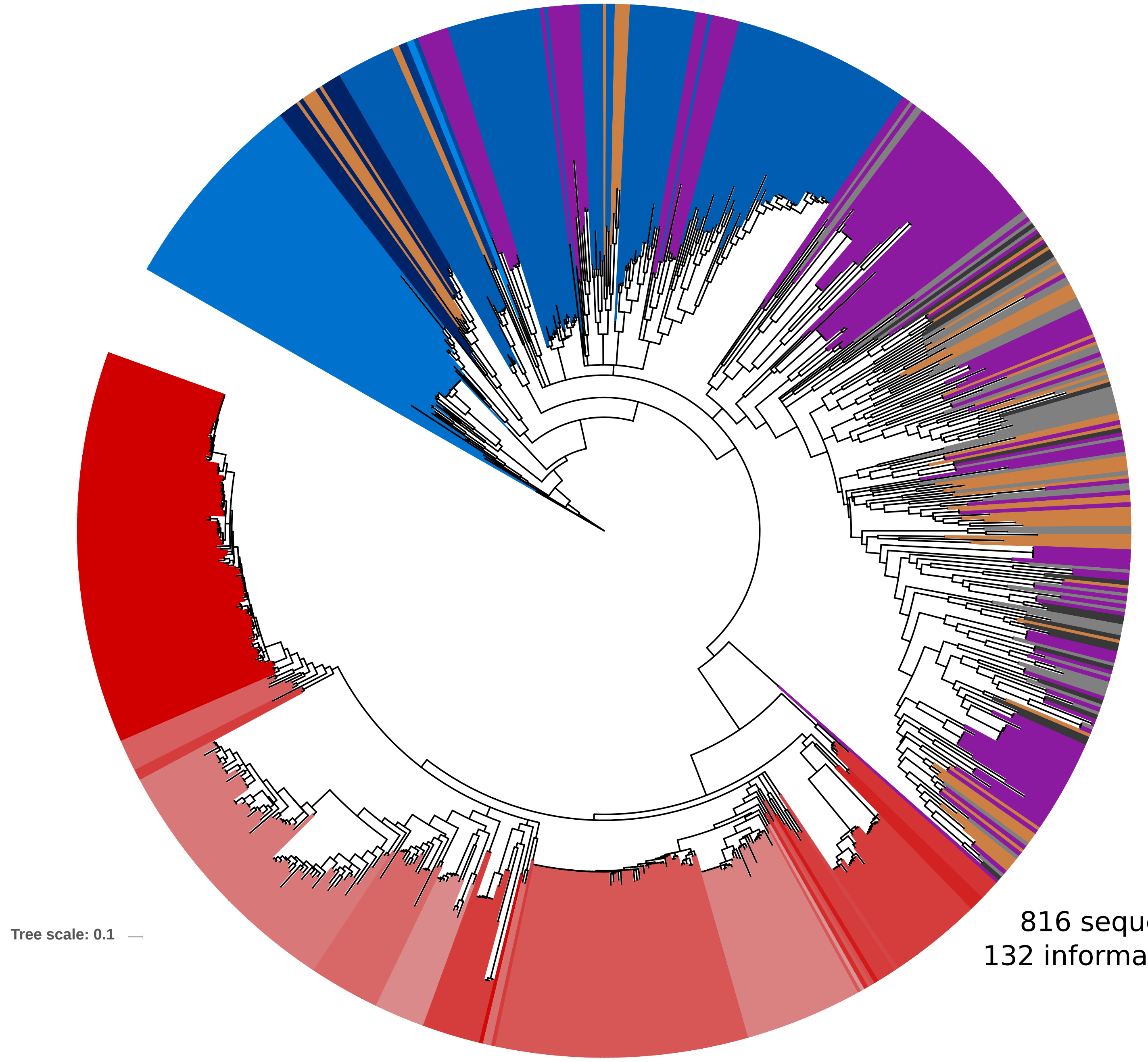

816 sequences,
132 informative sites

small subunit ribosomal protein S12 (K02950)
small subunit ribosomal protein S23e (K02973)

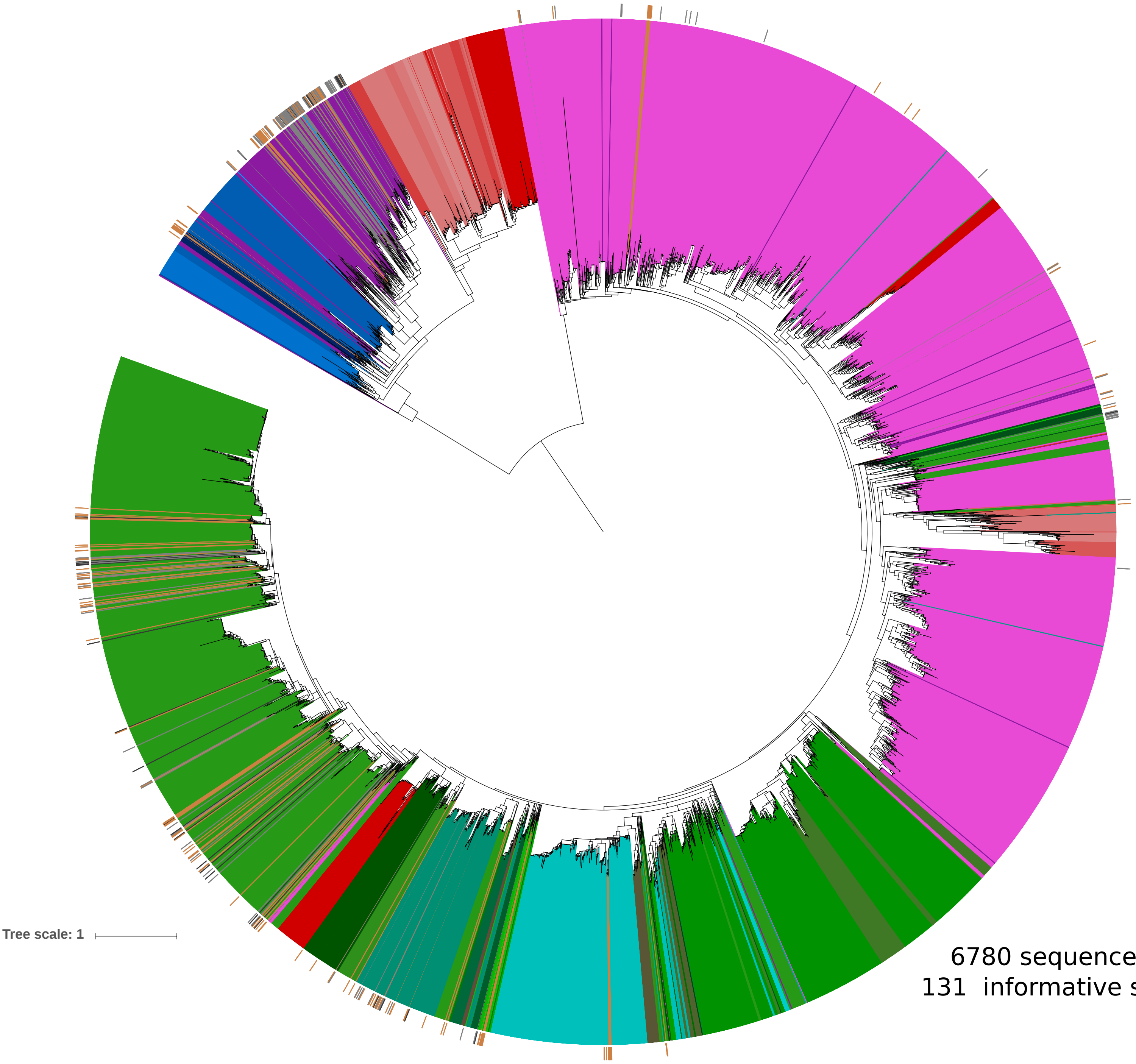

6780 sequences,
131 informative sites

small subunit ribosomal protein S13 (K02952)
small subunit ribosomal protein S18e (K02964)

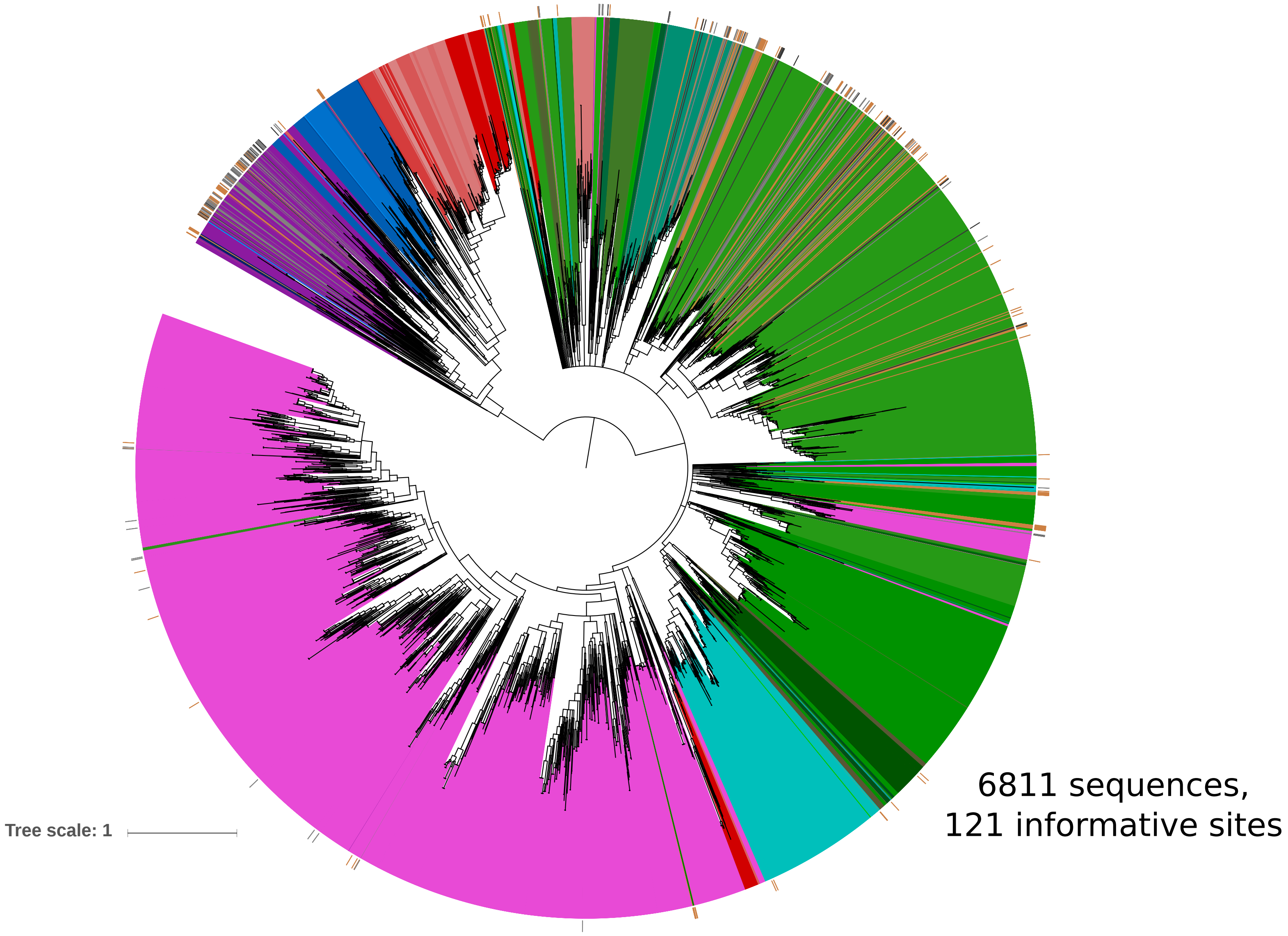

small subunit ribosomal protein S13e (K02953)
small subunit ribosomal protein (K02956)

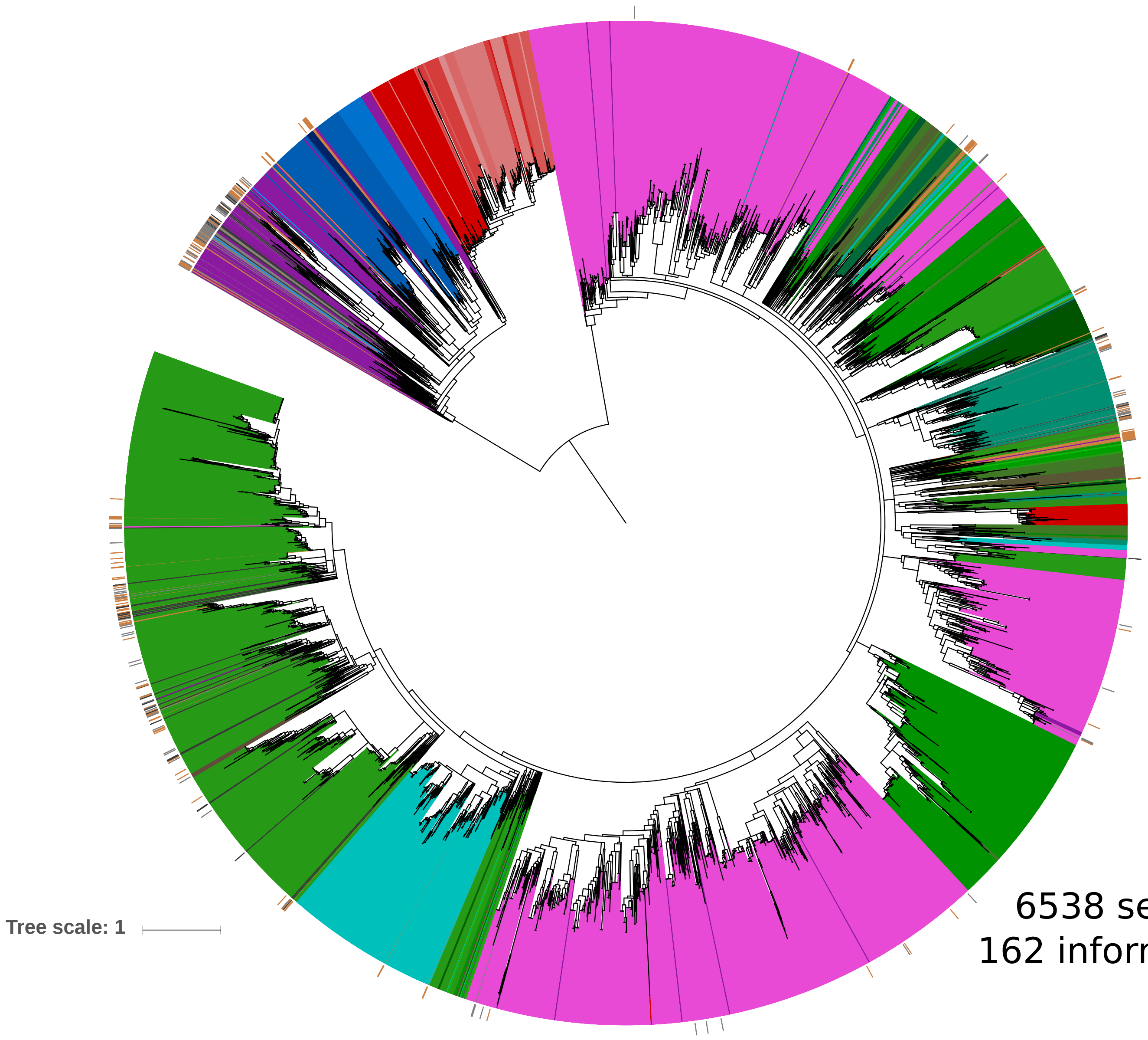

6538 sequences,
162 informative sites

small subunit ribosomal protein S14 (K02954)
small subunit ribosomal protein S29e (K02980)

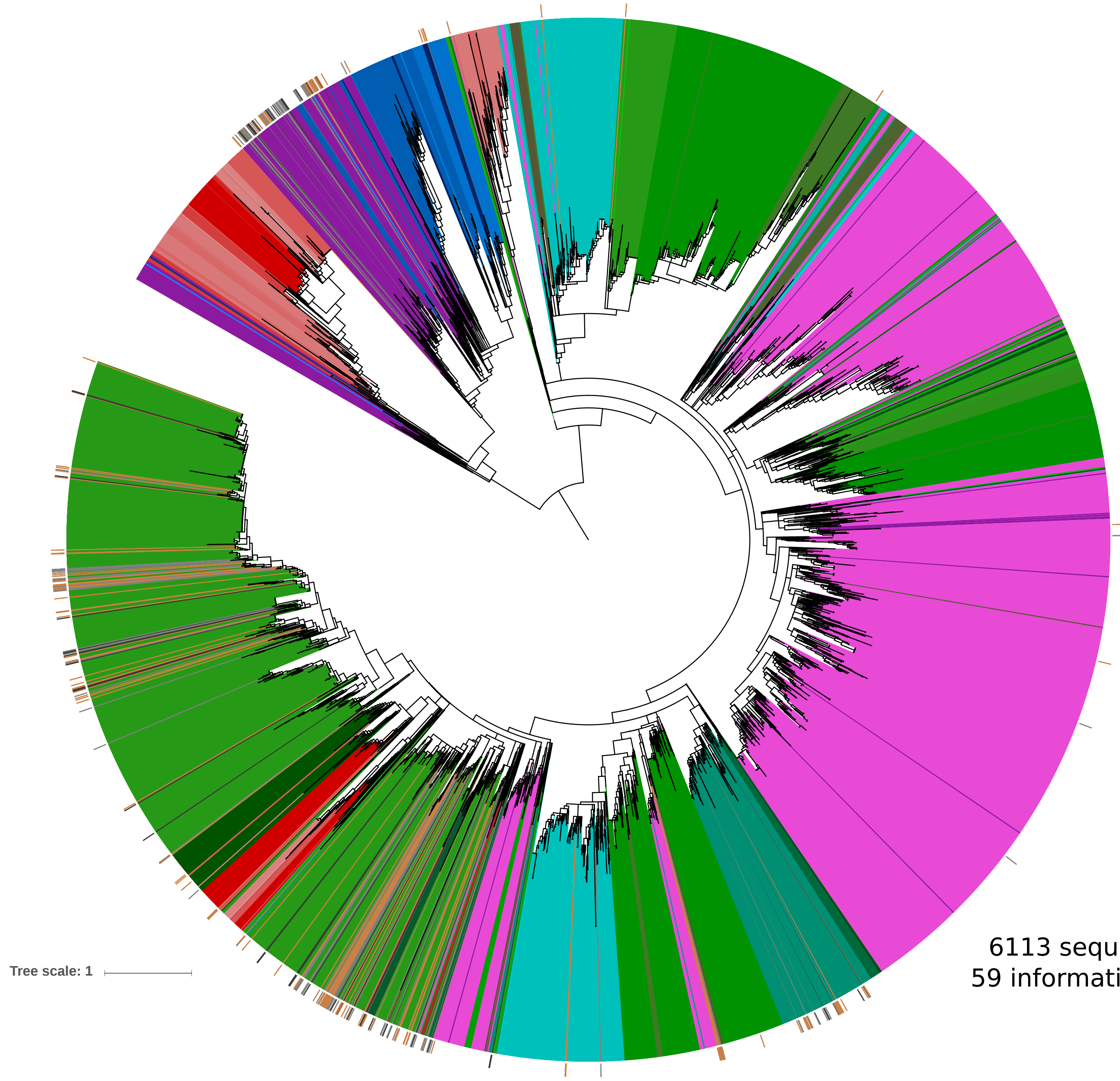

6113 sequences,
59 informative sites

small subunit ribosomal protein S15Ae (K02957)
small subunit ribosomal protein S8 (K02994)

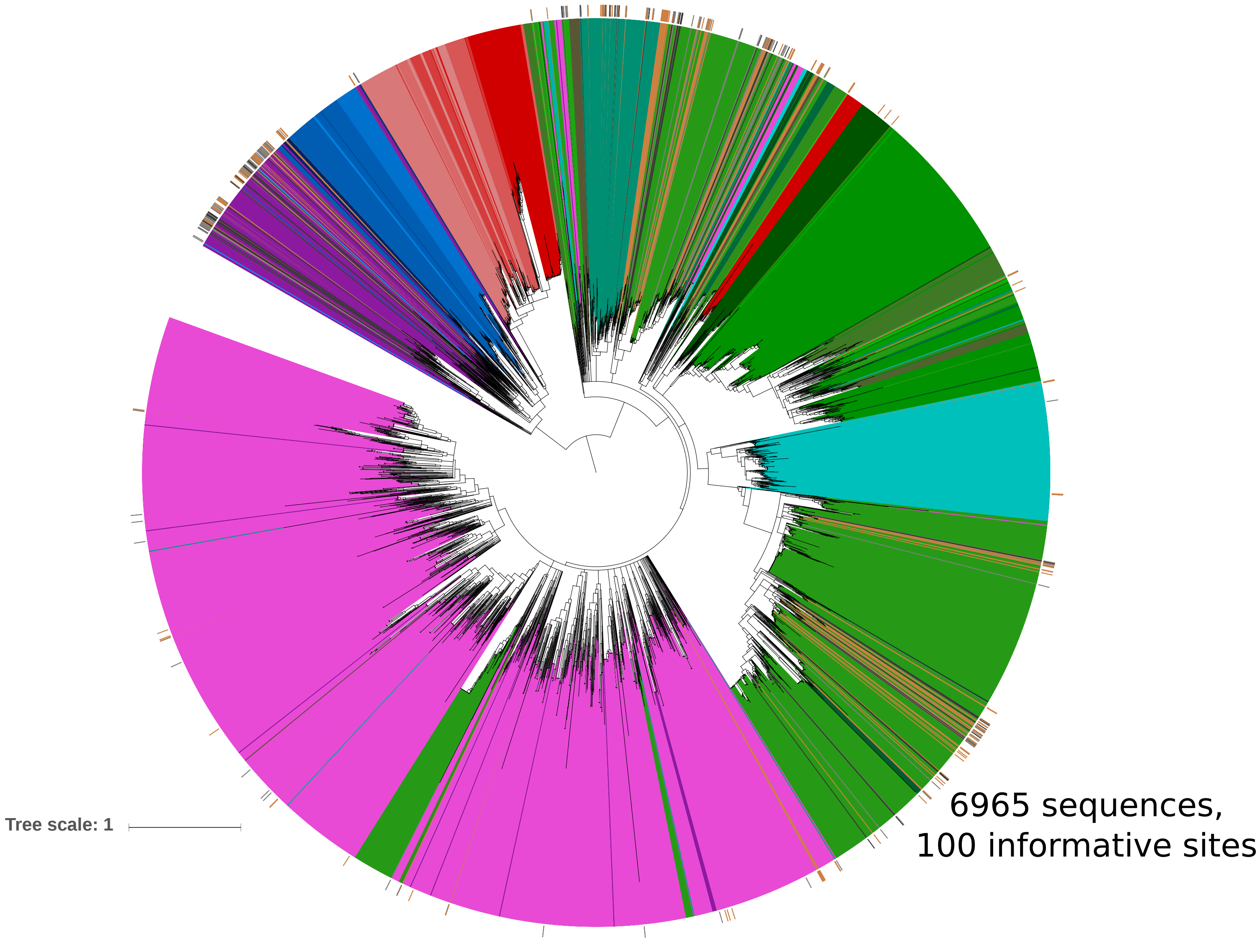

small subunit ribosomal protein S15e (K02958)
small subunit ribosomal protein S19 (K02965)

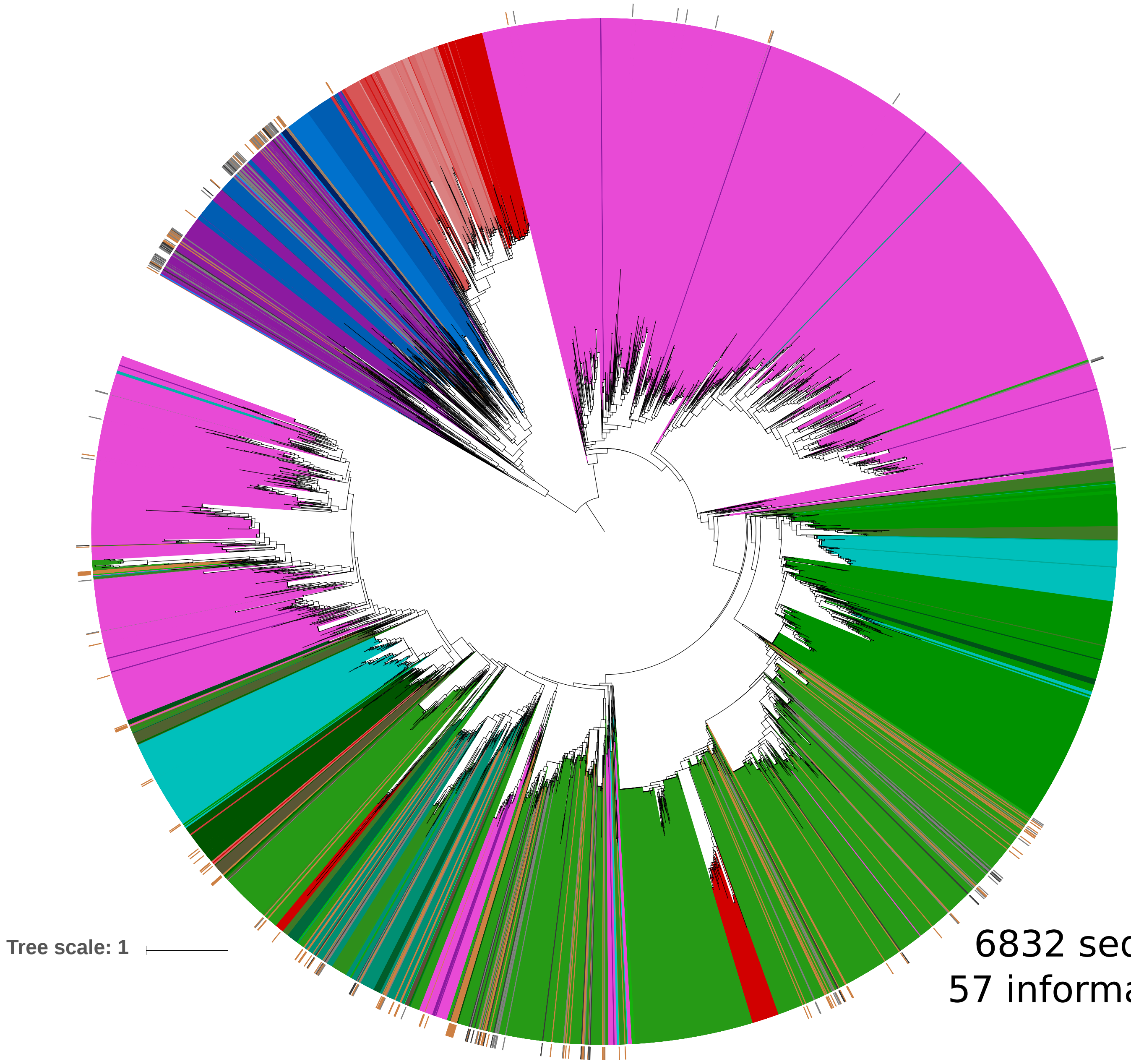

6832 sequences,
57 informative sites

small subunit ribosomal protein S16 (K02959)

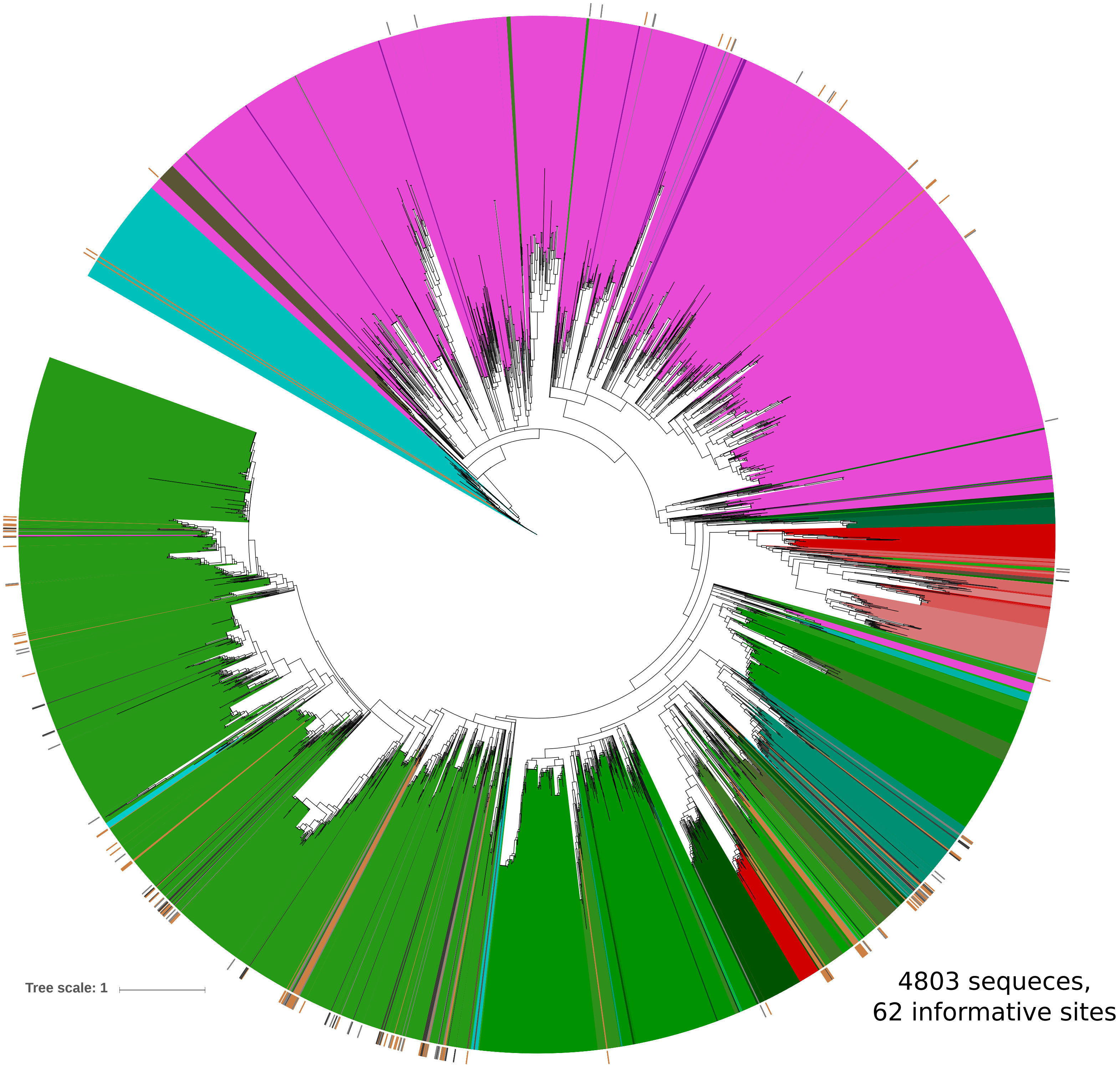

small subunit ribosomal protein S16e (K02960) small subunit ribosomal protein S9 (K02996)

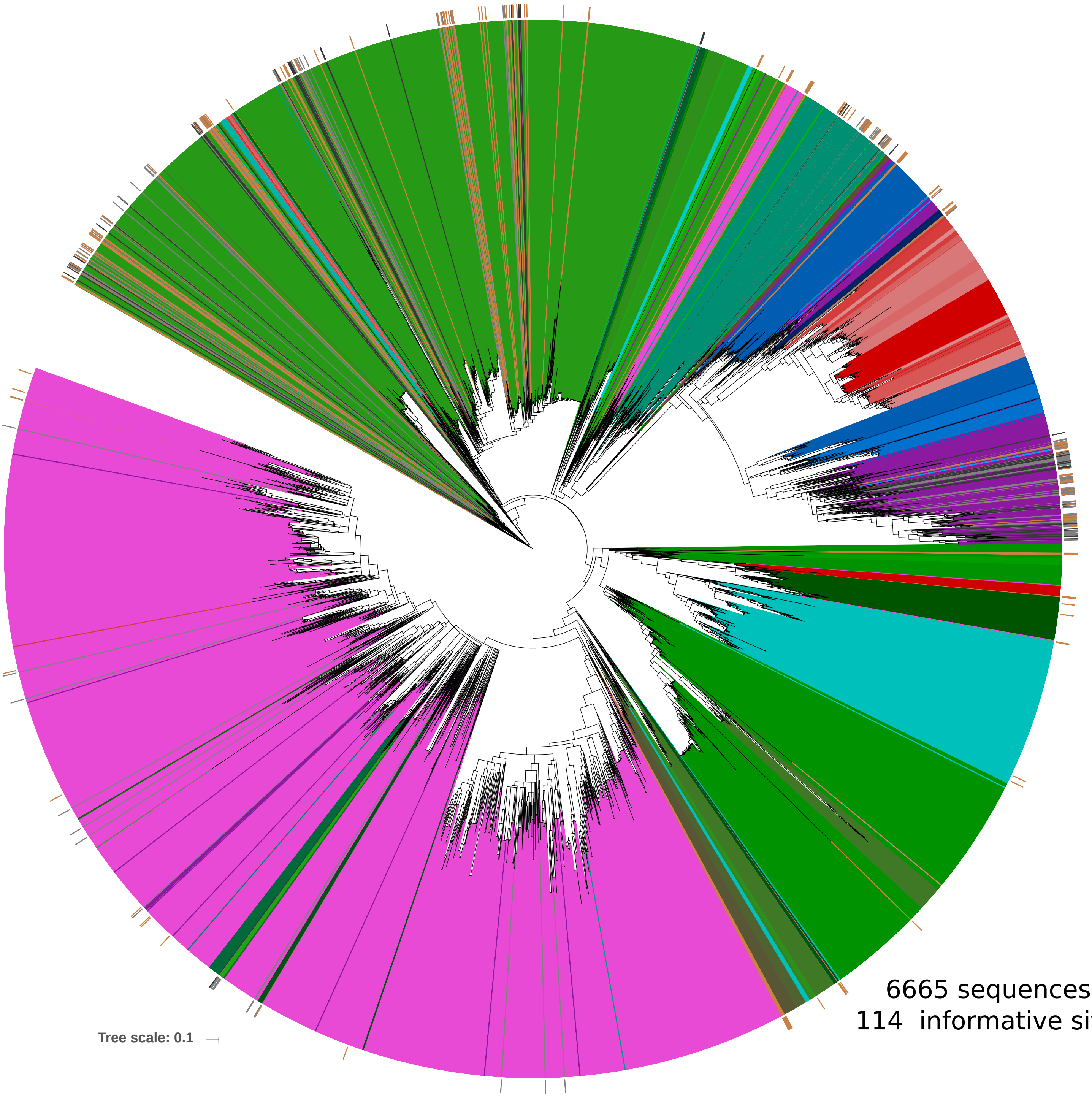

6665 sequences,
114 informative sites

small subunit ribosomal protein S17e (K02962)

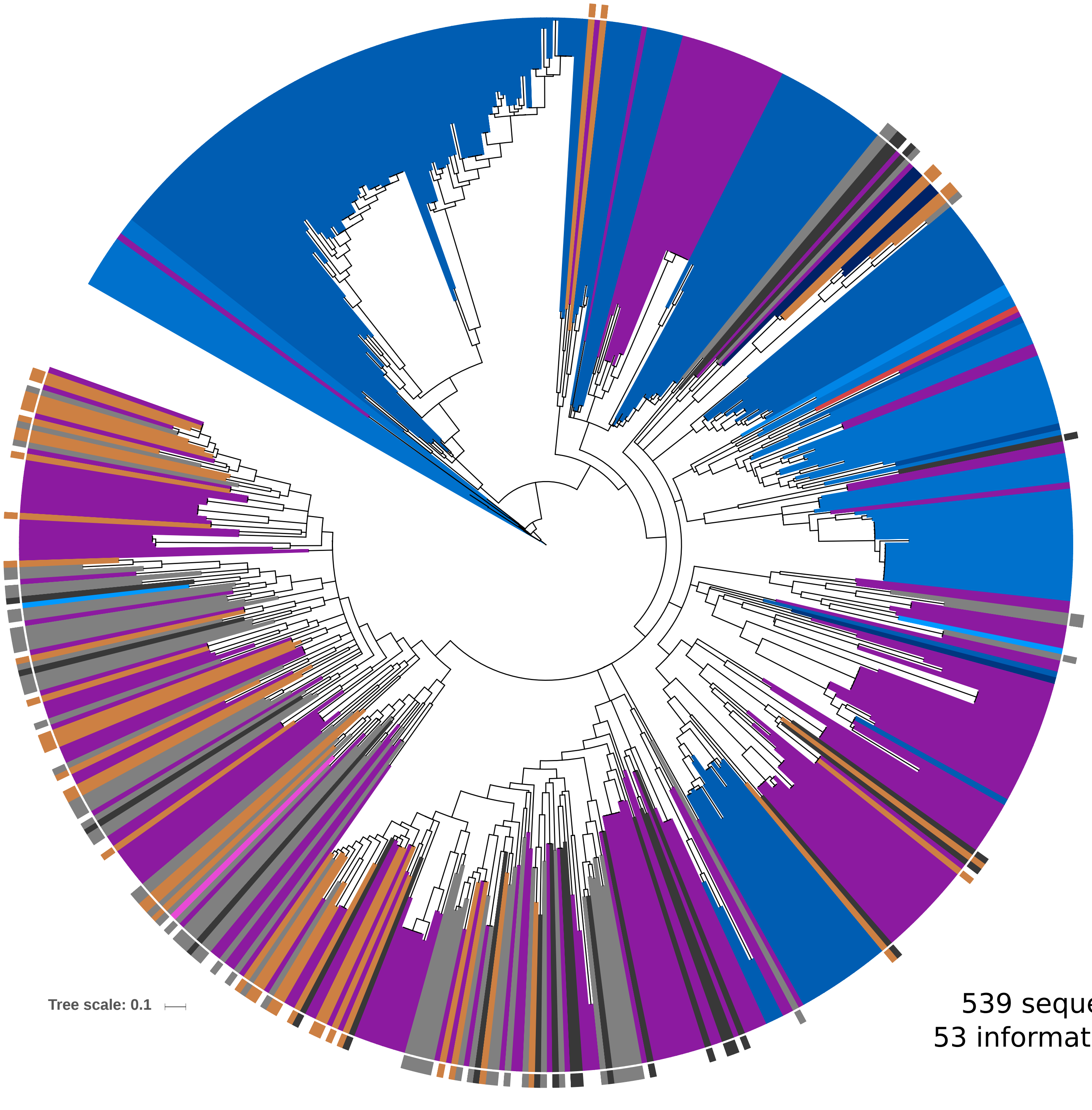

539 sequences,
53 informative sites

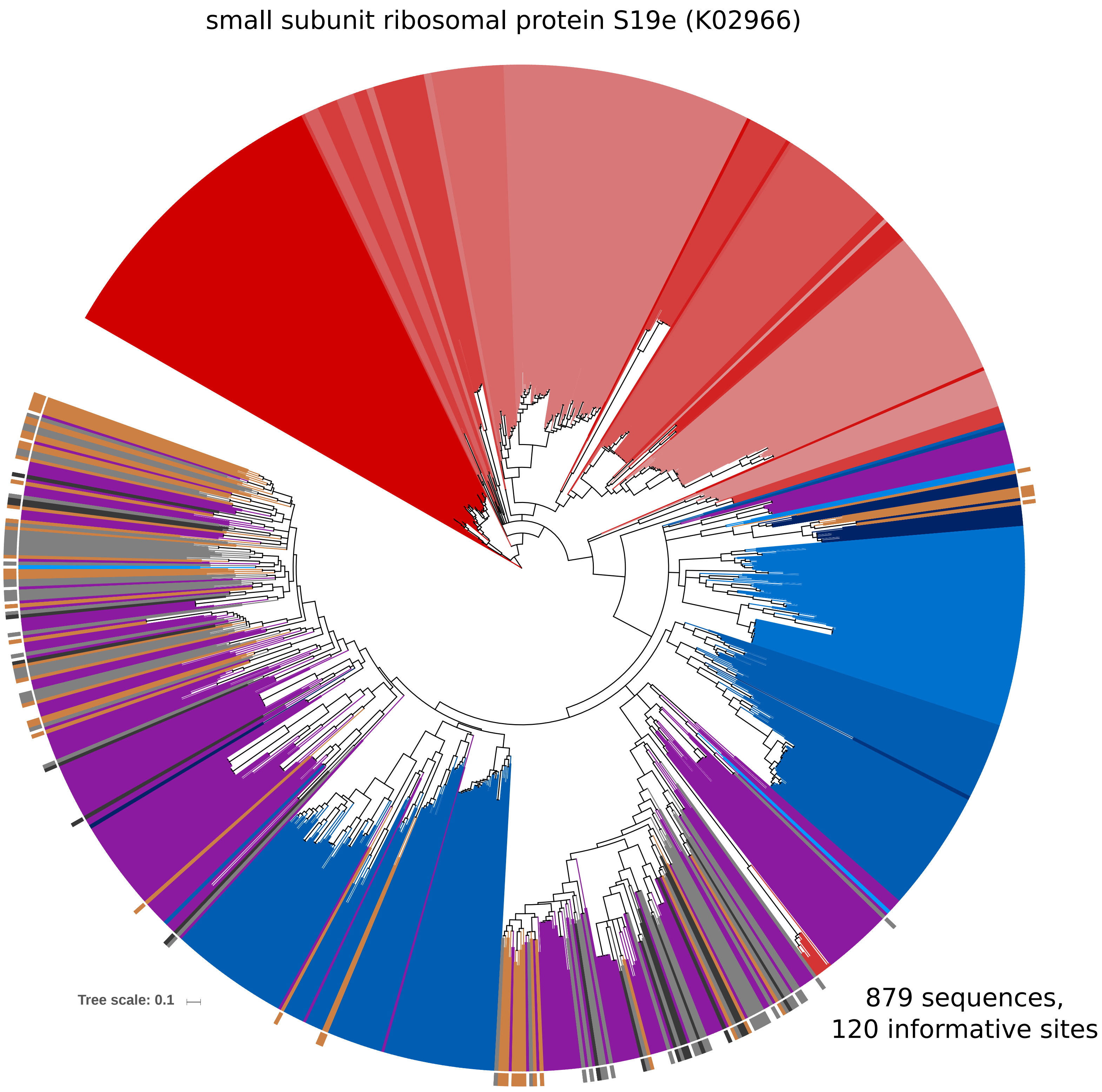
small subunit ribosomal protein S19e (K02966)
Tree scale: 0.1
879 sequences,
120 informative sites

small subunit ribosomal protein S2 (K02967) small subunit ribosomal protein SAe (K02998)

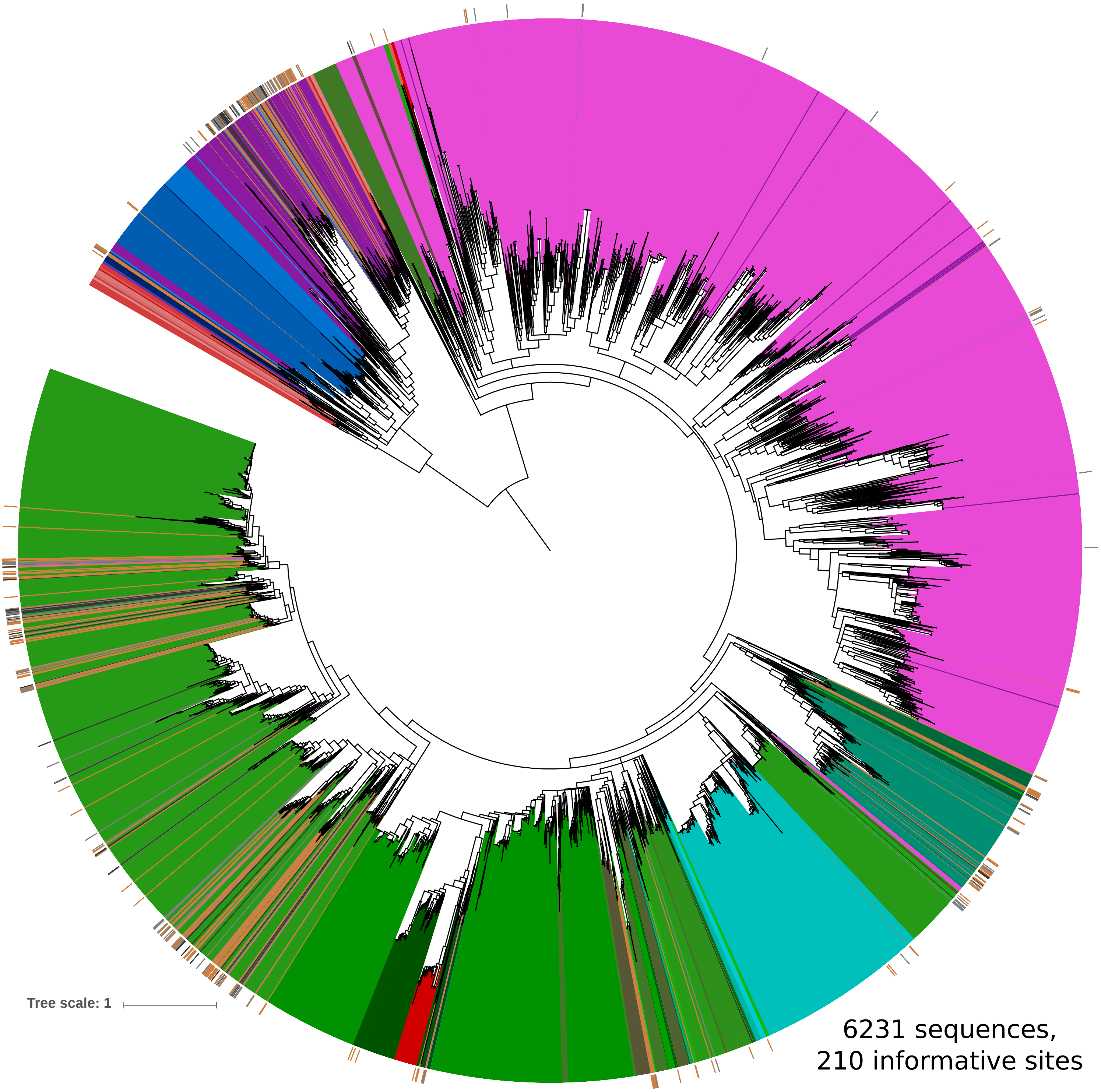

6231 sequences,
210 informative sites

small subunit ribosomal protein S20 (K02968)

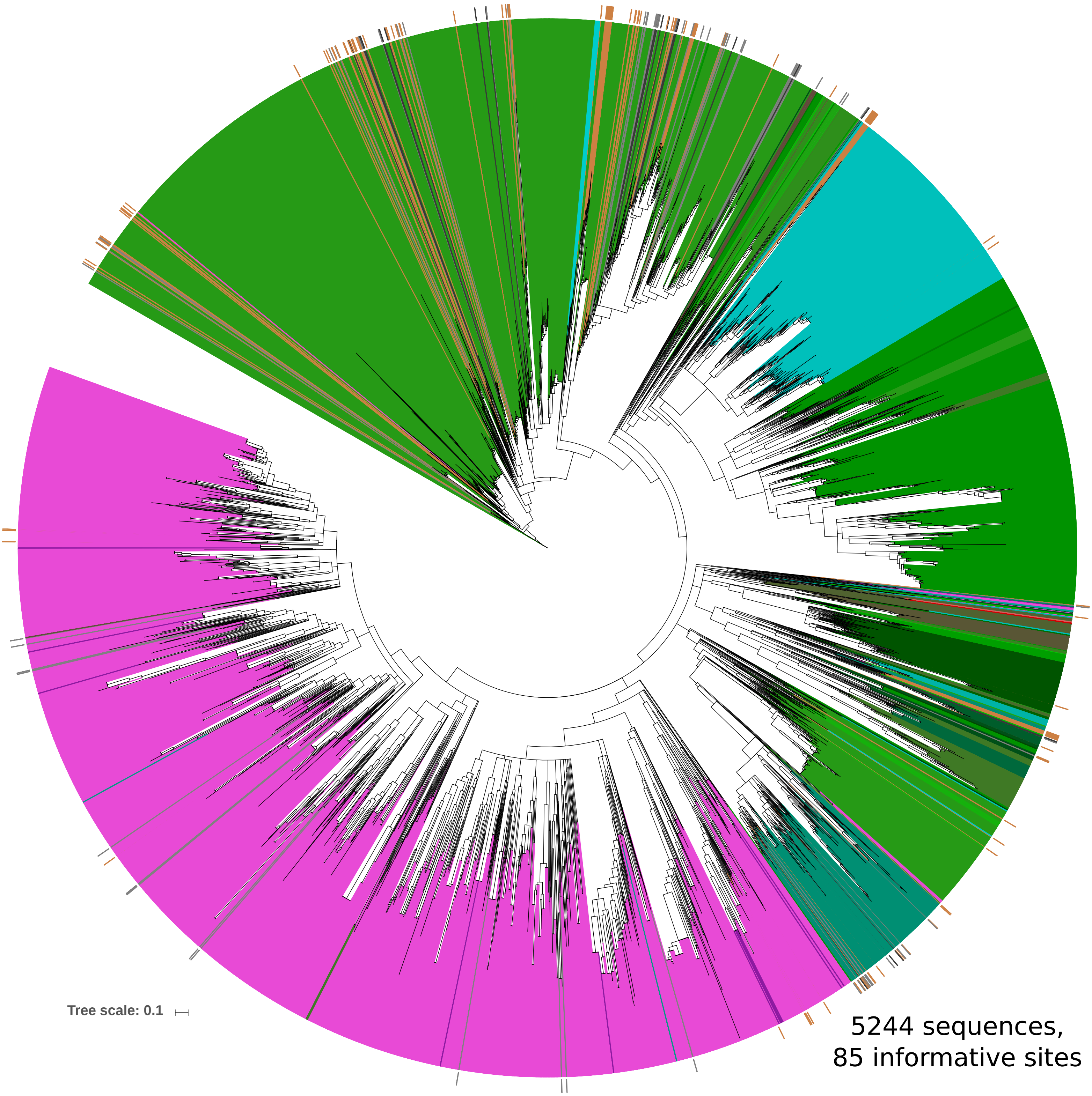

5244 sequences,
85 informative sites

small subunit ribosomal protein S21 (K02970)

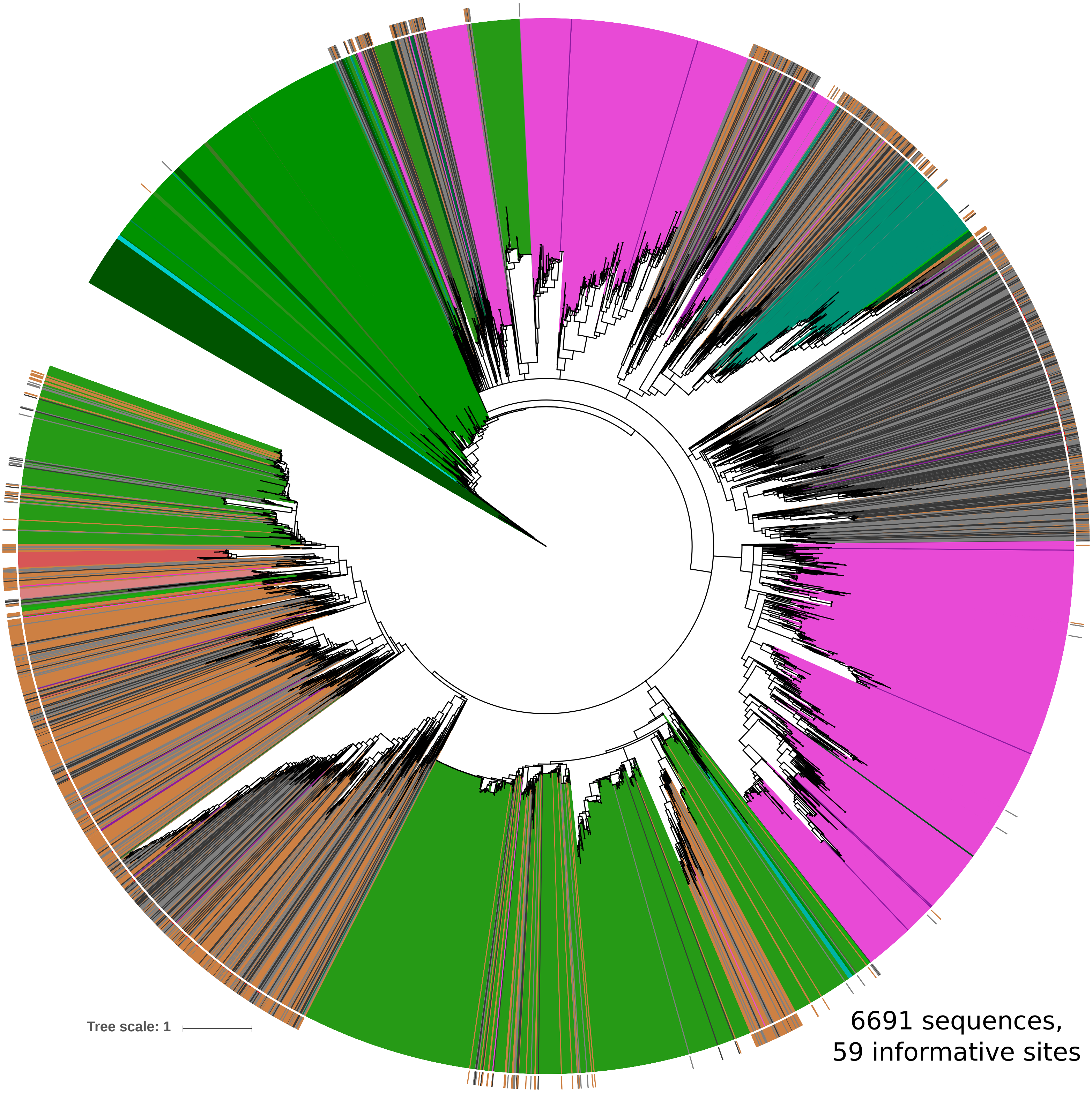

6691 sequences,
59 informative sites

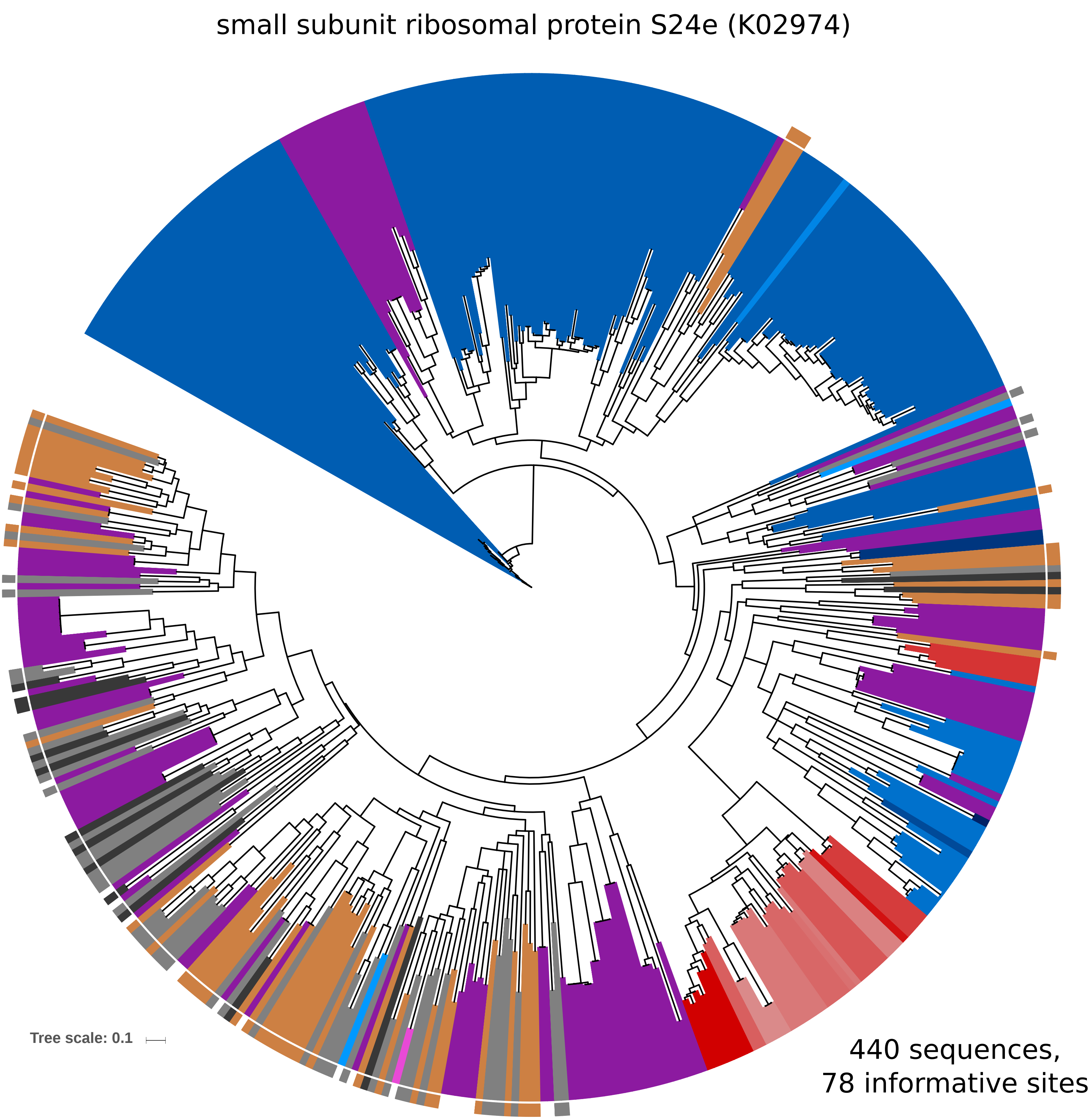
small subunit ribosomal protein S24e (K02974)
Tree scale: 0.1
440 sequences,
78 informative sites

small subunit ribosomal protein S26e (K02976)

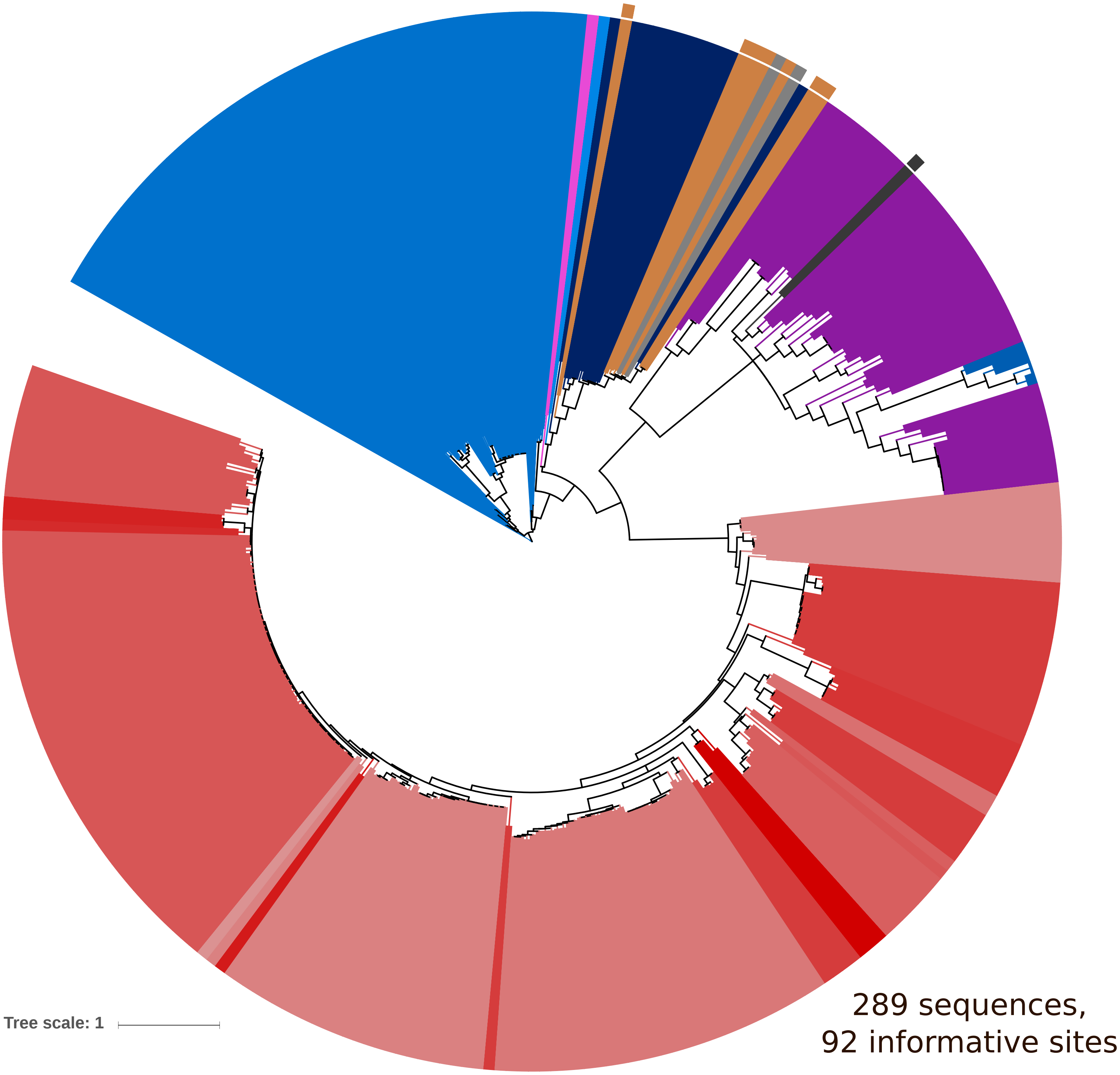

small subunit ribosomal protein S27e (K02978)

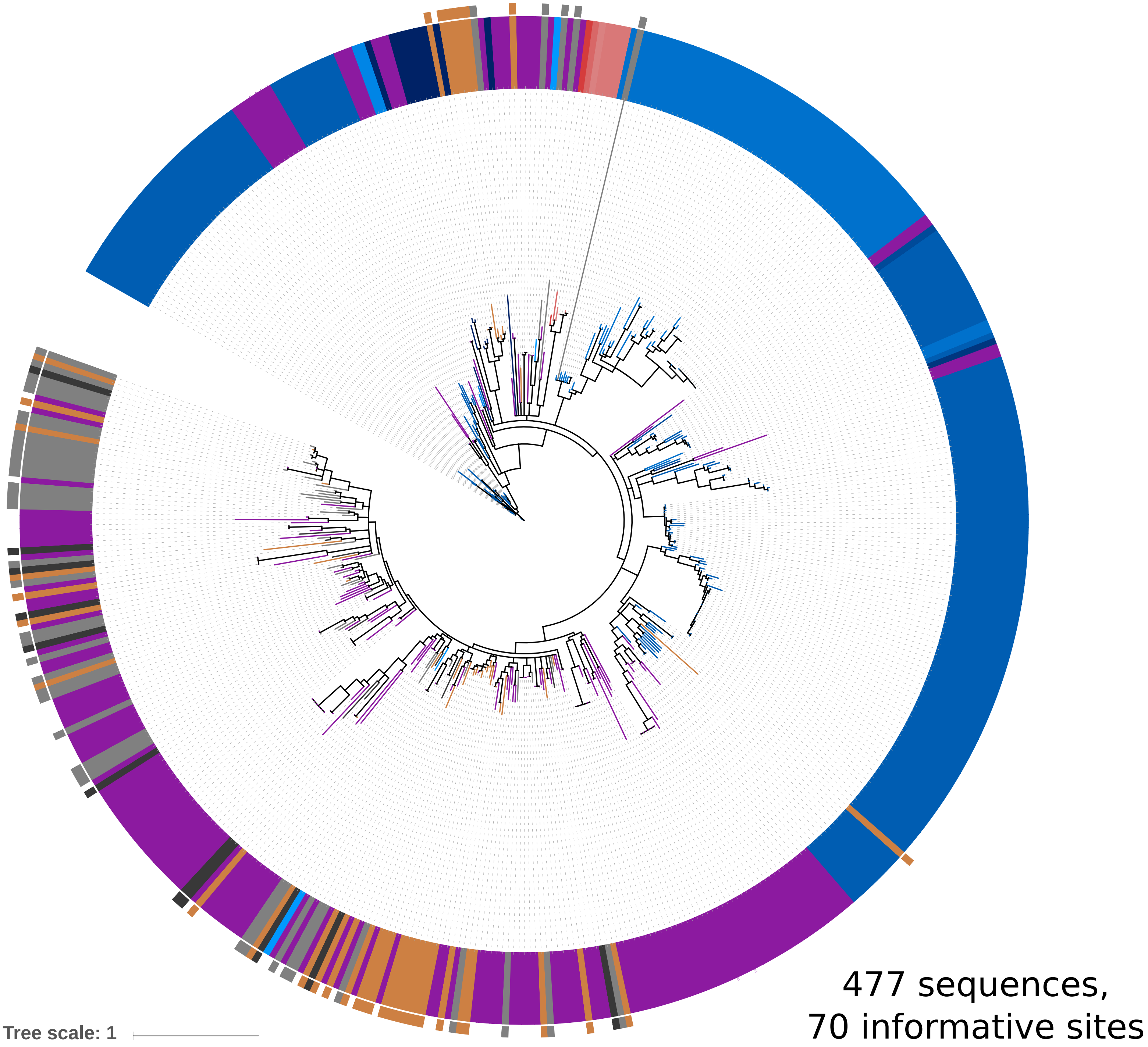

Ribosome K02979 RP-S28e (K02979)

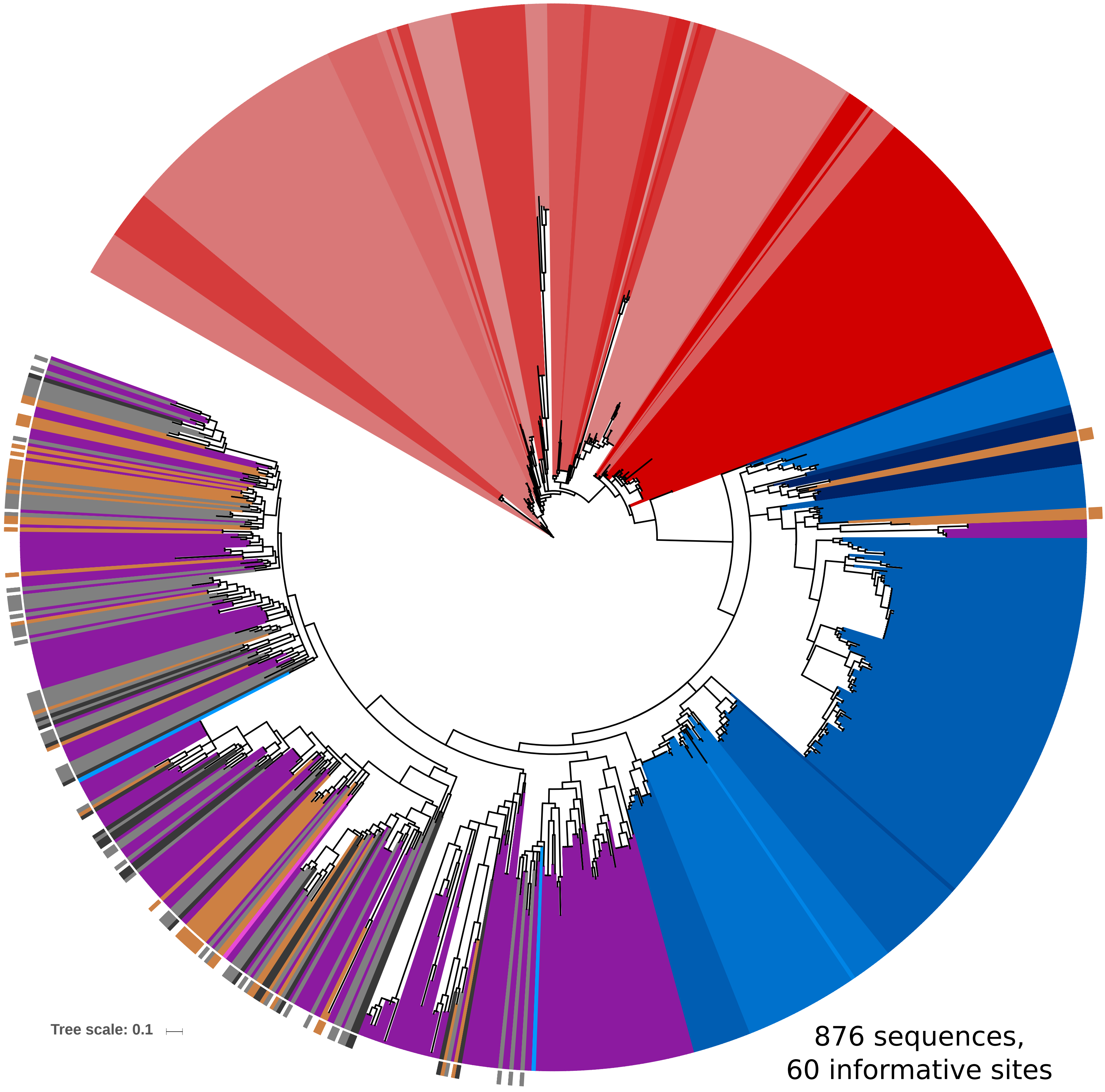

Ribosome K02984 RP-S3Ae (K02984)

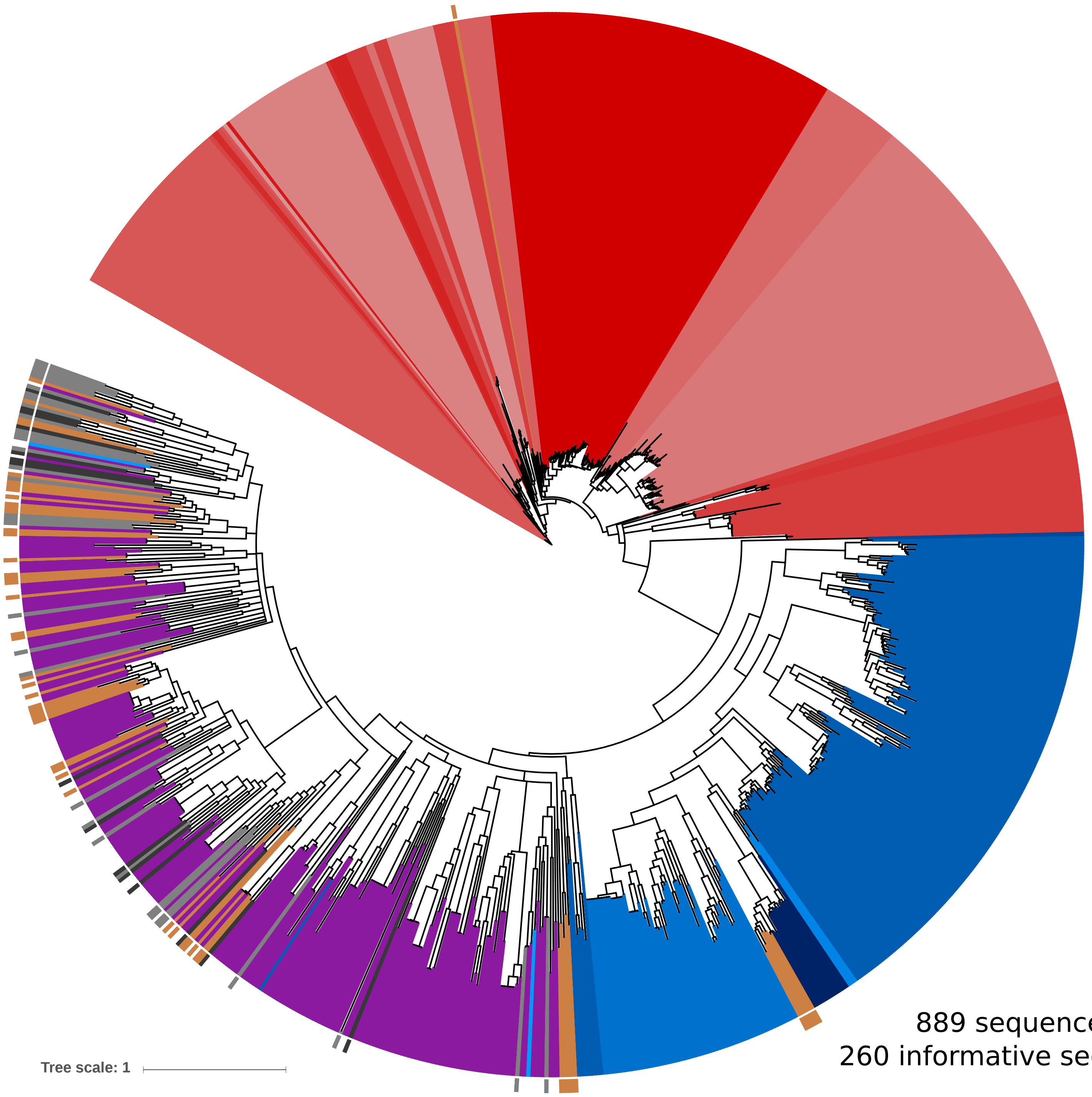

889 sequences,
260 informative sequences

small subunit ribosomal protein S4 (K02986), small subunit ribosomal protein S9e (K02997)

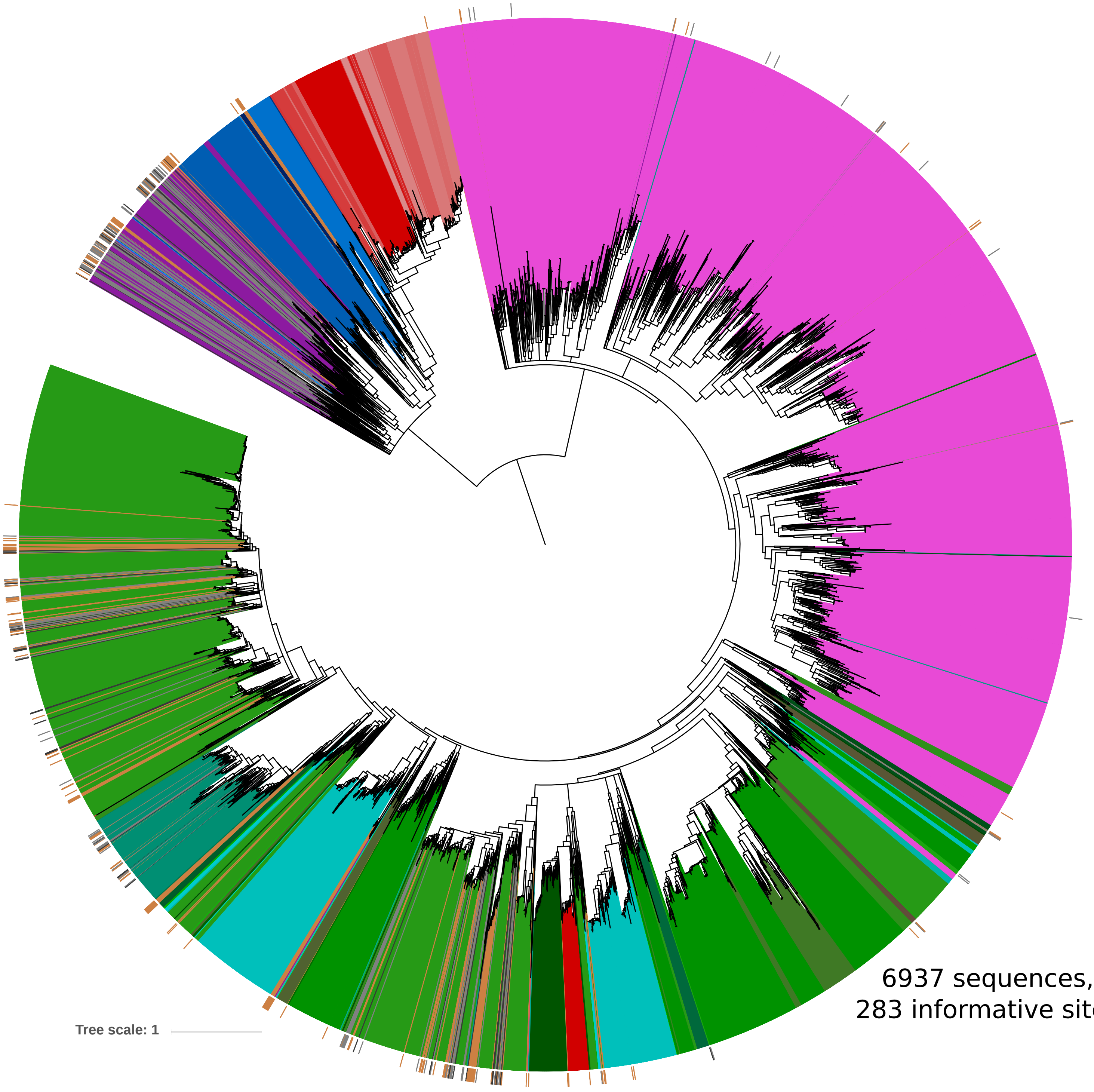

6937 sequences,
283 informative sites

small subunit ribosomal protein S4e (K02987)

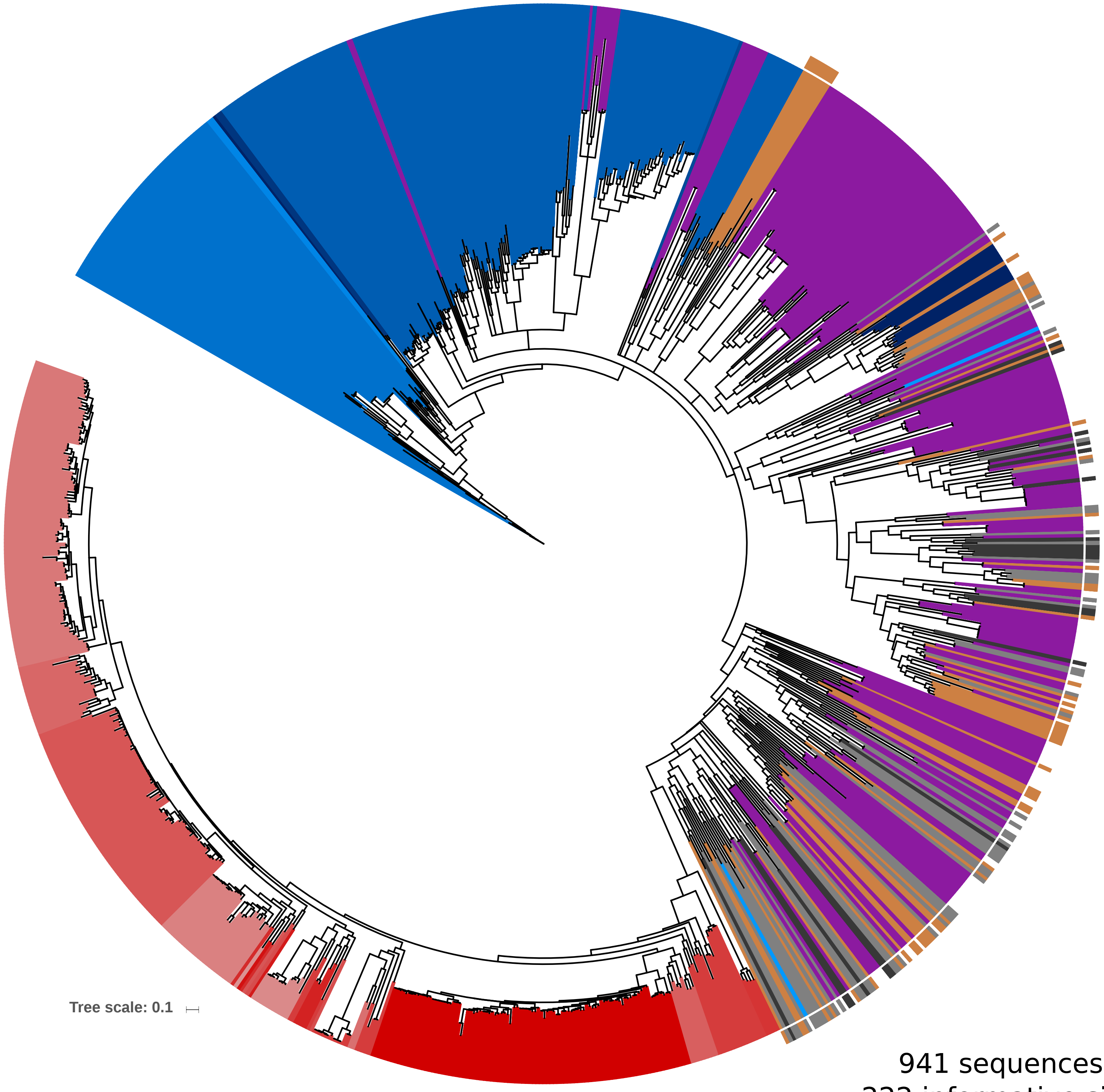

941 sequences,
222 informative sites

small subunit ribosomal protein S6e (K02991)

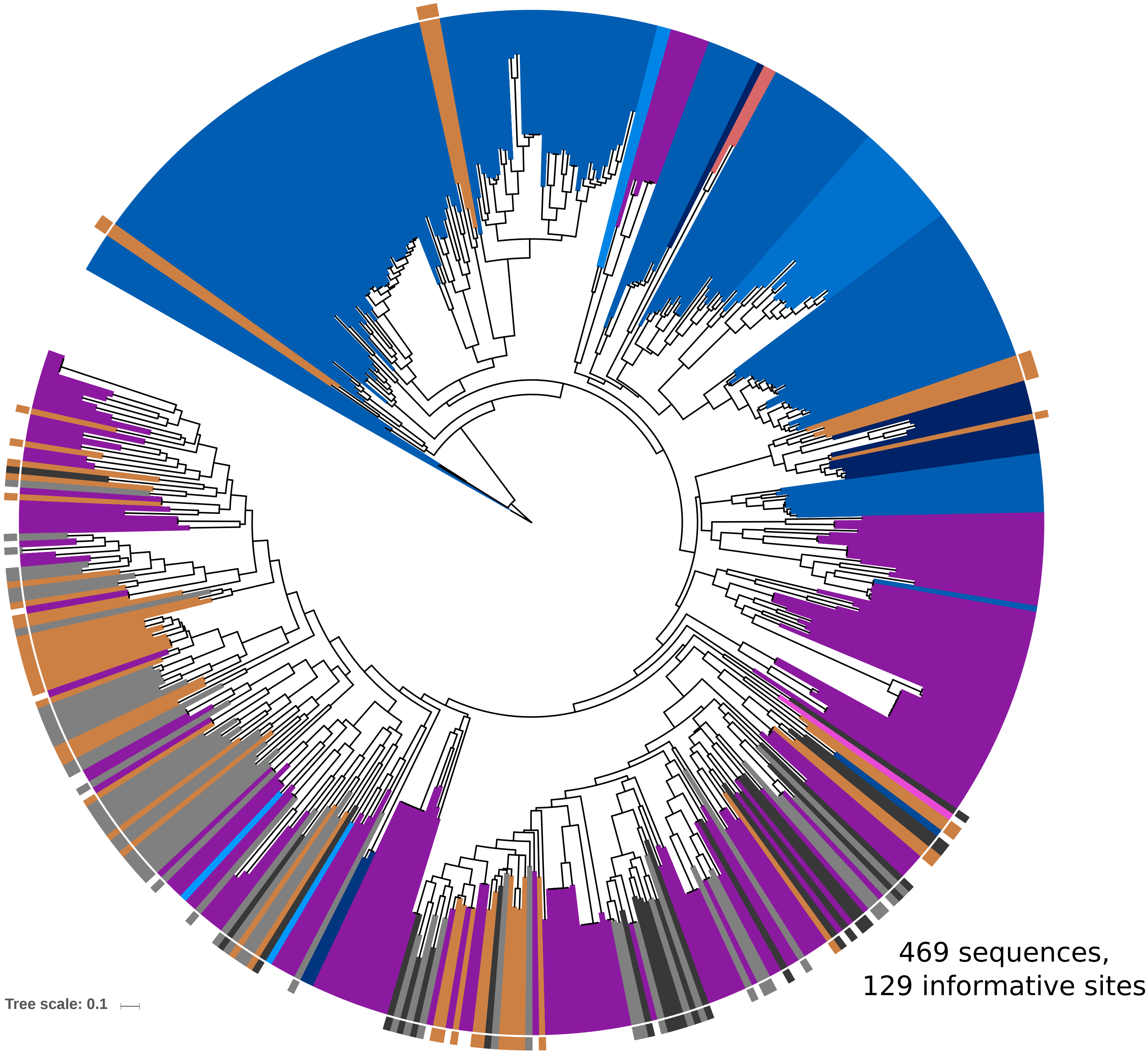

small subunit ribosomal protein S8e (K02995)

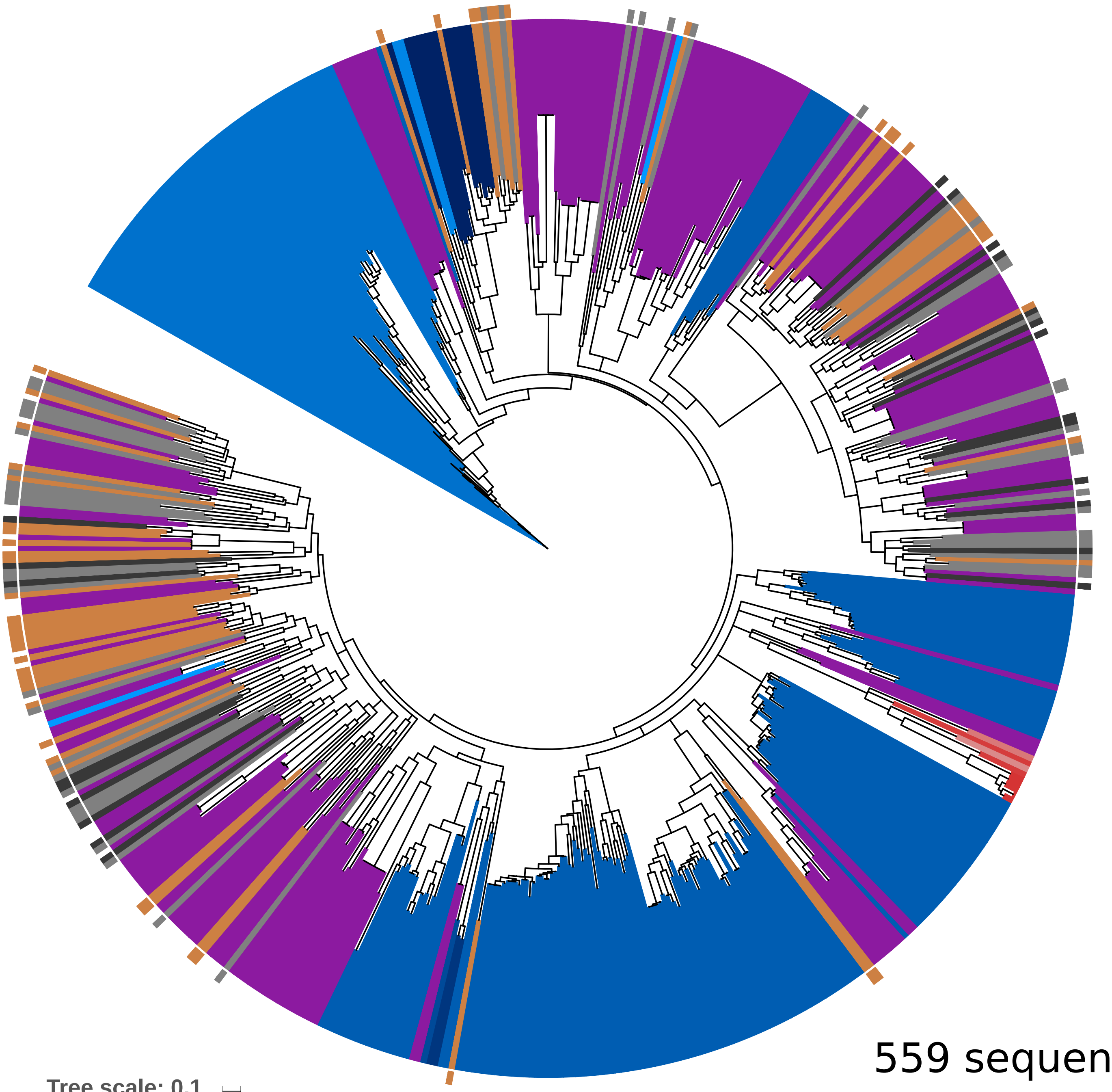

559 sequences
114 informative sites

large subunit ribosomal protein L33 (K02913)

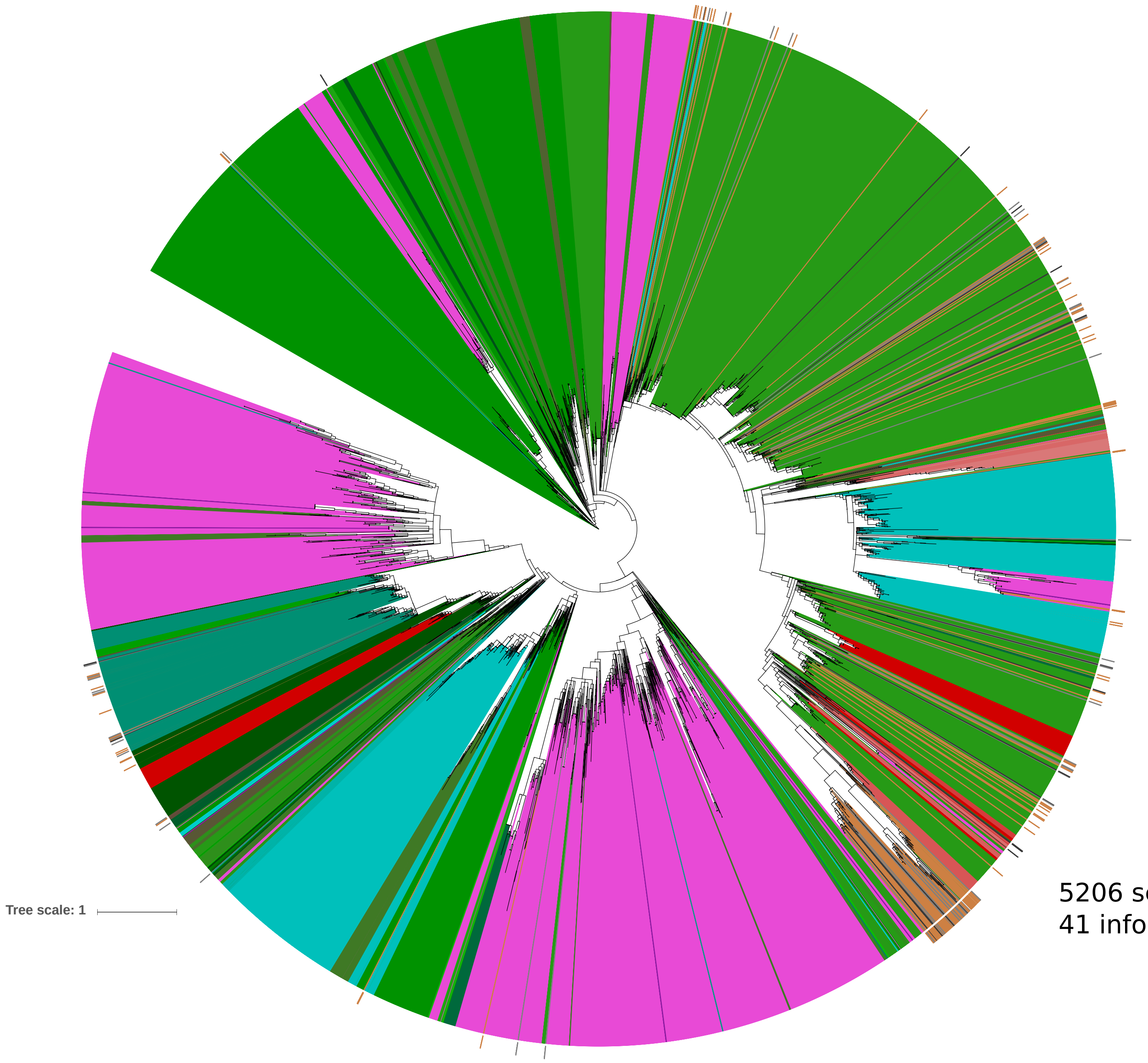

5206 sequences,
41 informative sites

large subunit ribosomal protein L34 K02914)

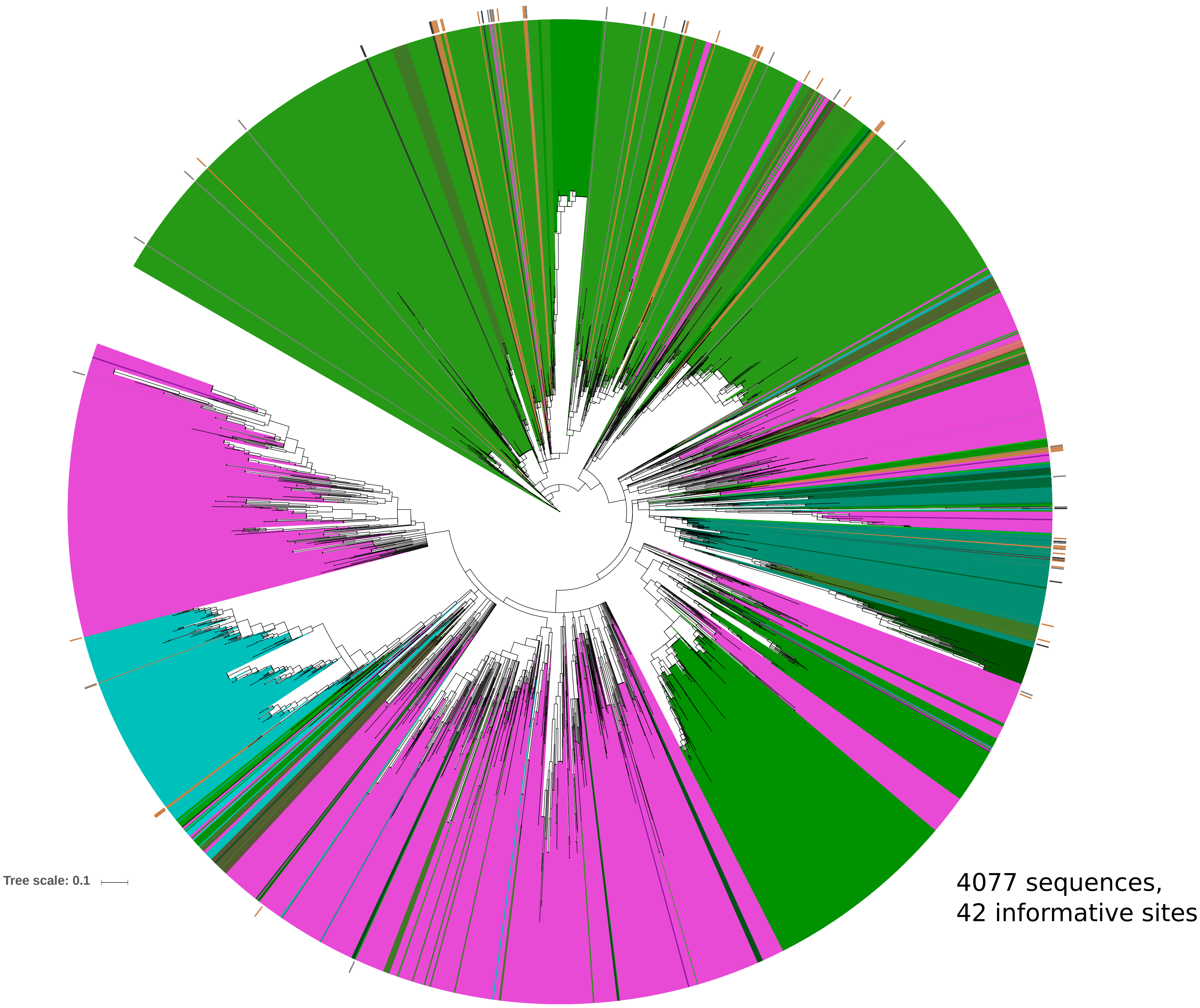

large subunit ribosomal protein L35Ae

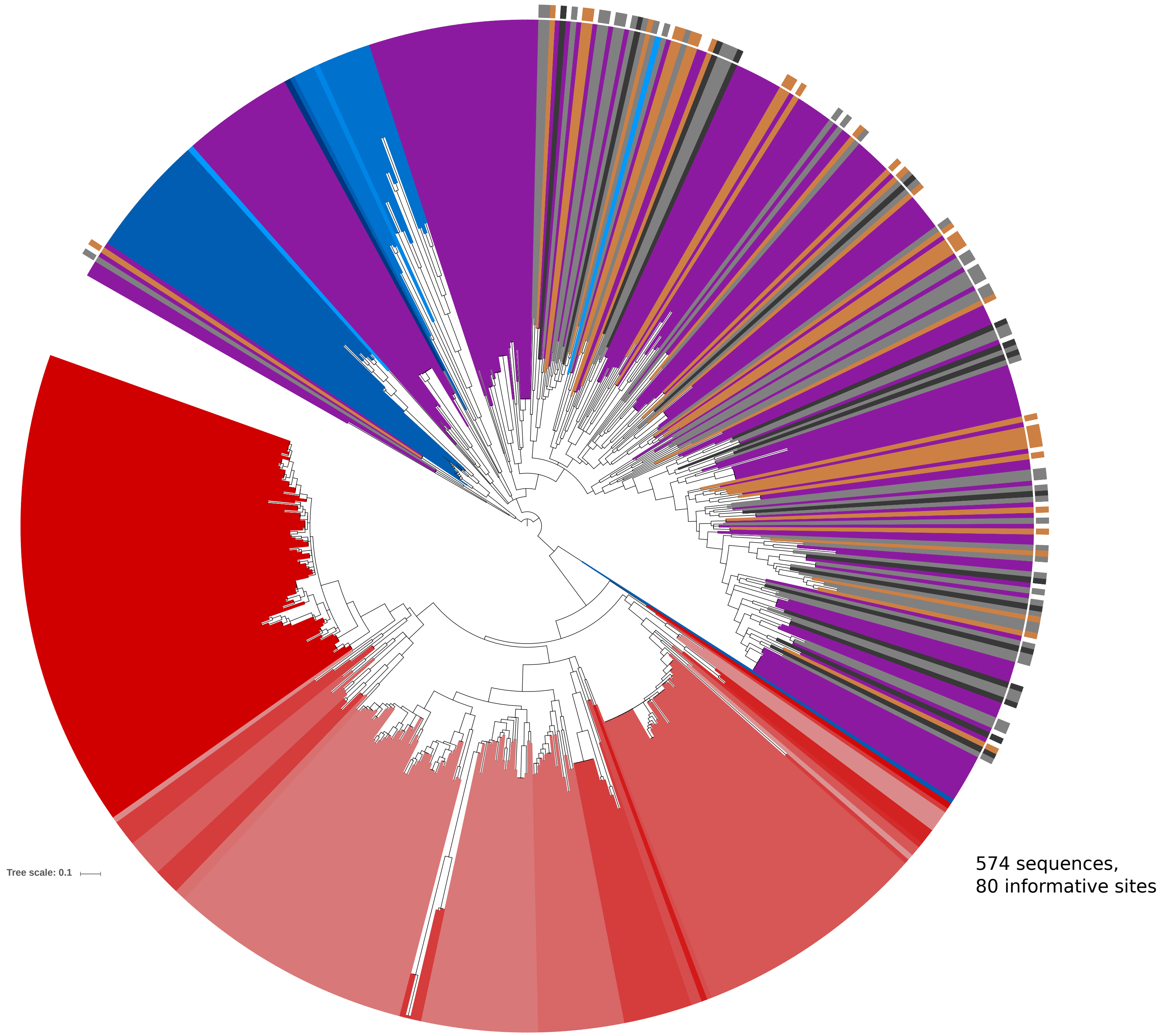

574 sequences,
80 informative sites

large subunit ribosomal protein L36 (K02919)

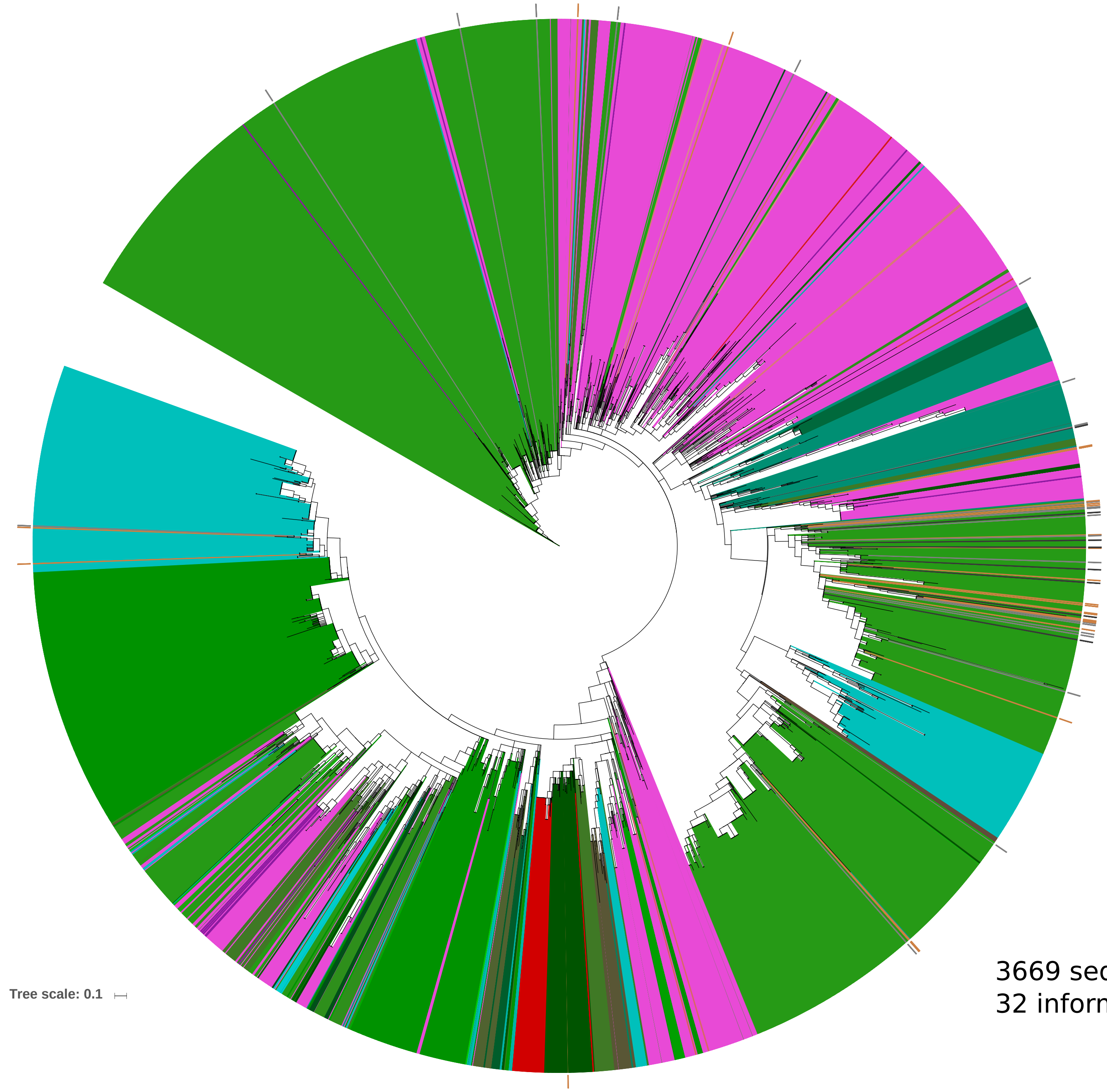

3669 sequences,
32 informative sites

large subunit ribosomal protein L37Ae (K02921)

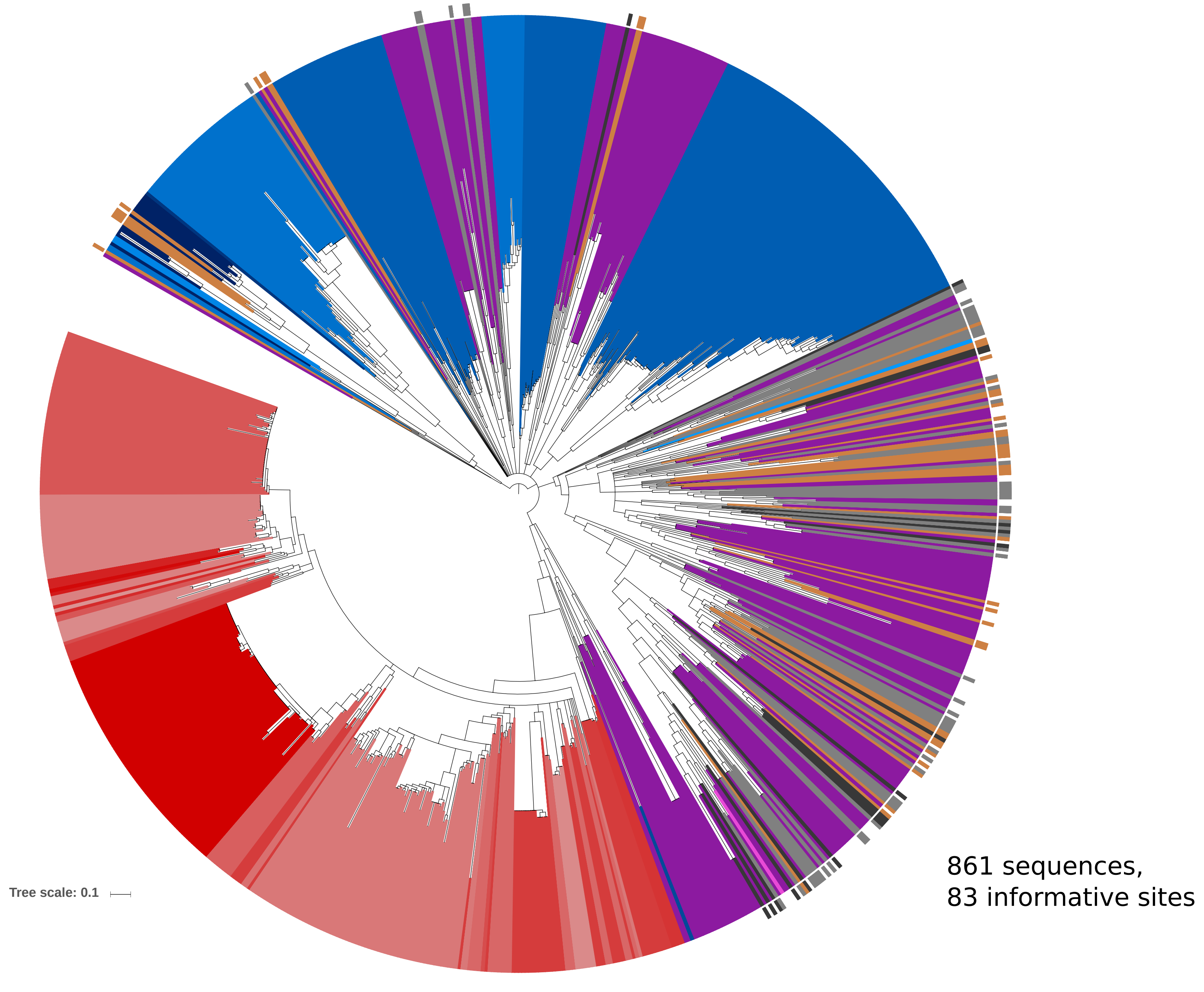

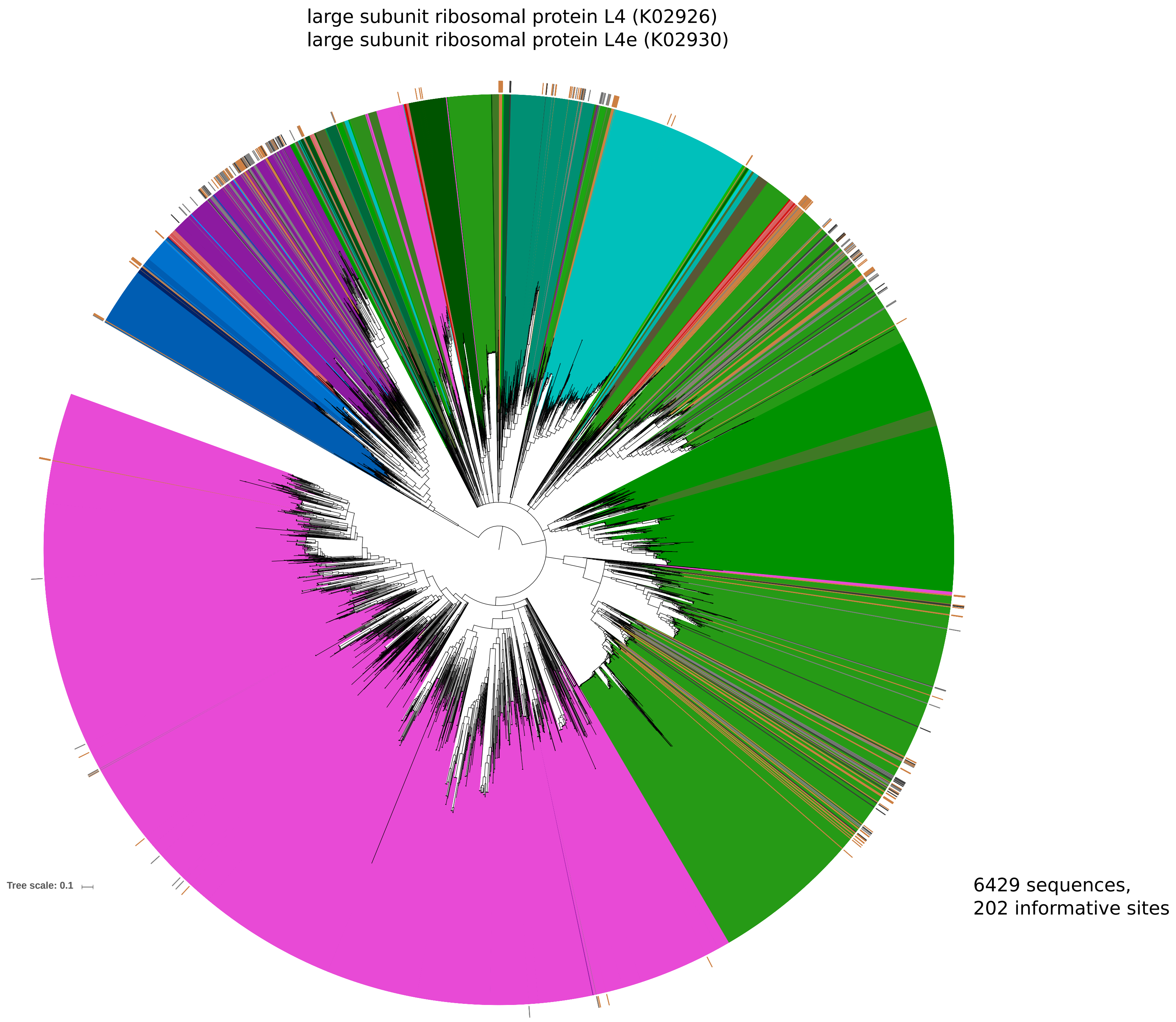
large subunit ribosomal protein L4 (K02926)
large subunit ribosomal protein L4e (K02930)
Tree scale: 0.1
6429 sequences,
202 informative sites

large subunit ribosomal protein L40e (K02927)
small subunit ribosomal protein S27Ae (K02977)
small subunit ribosomal protein S30e (K02983)

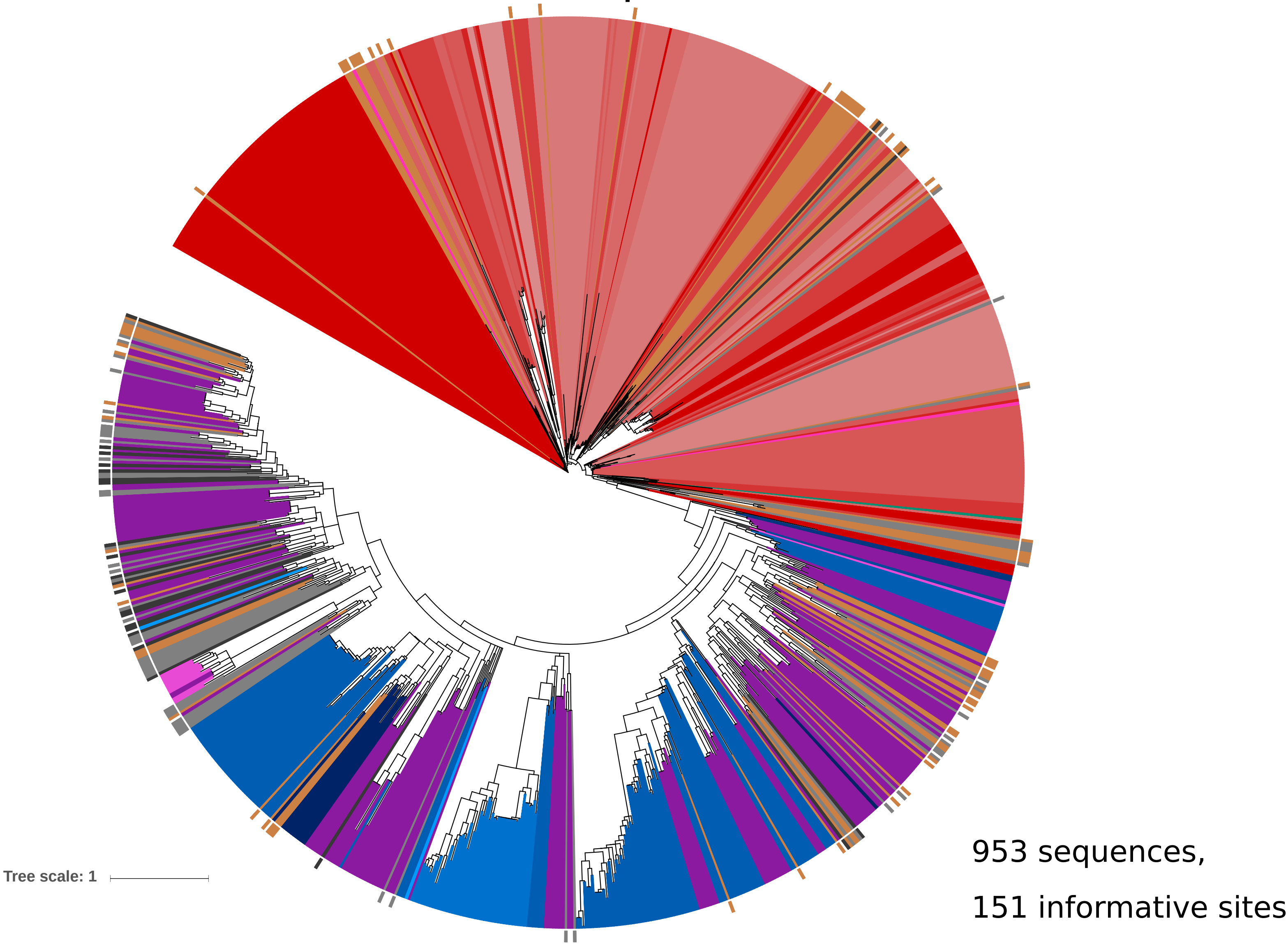

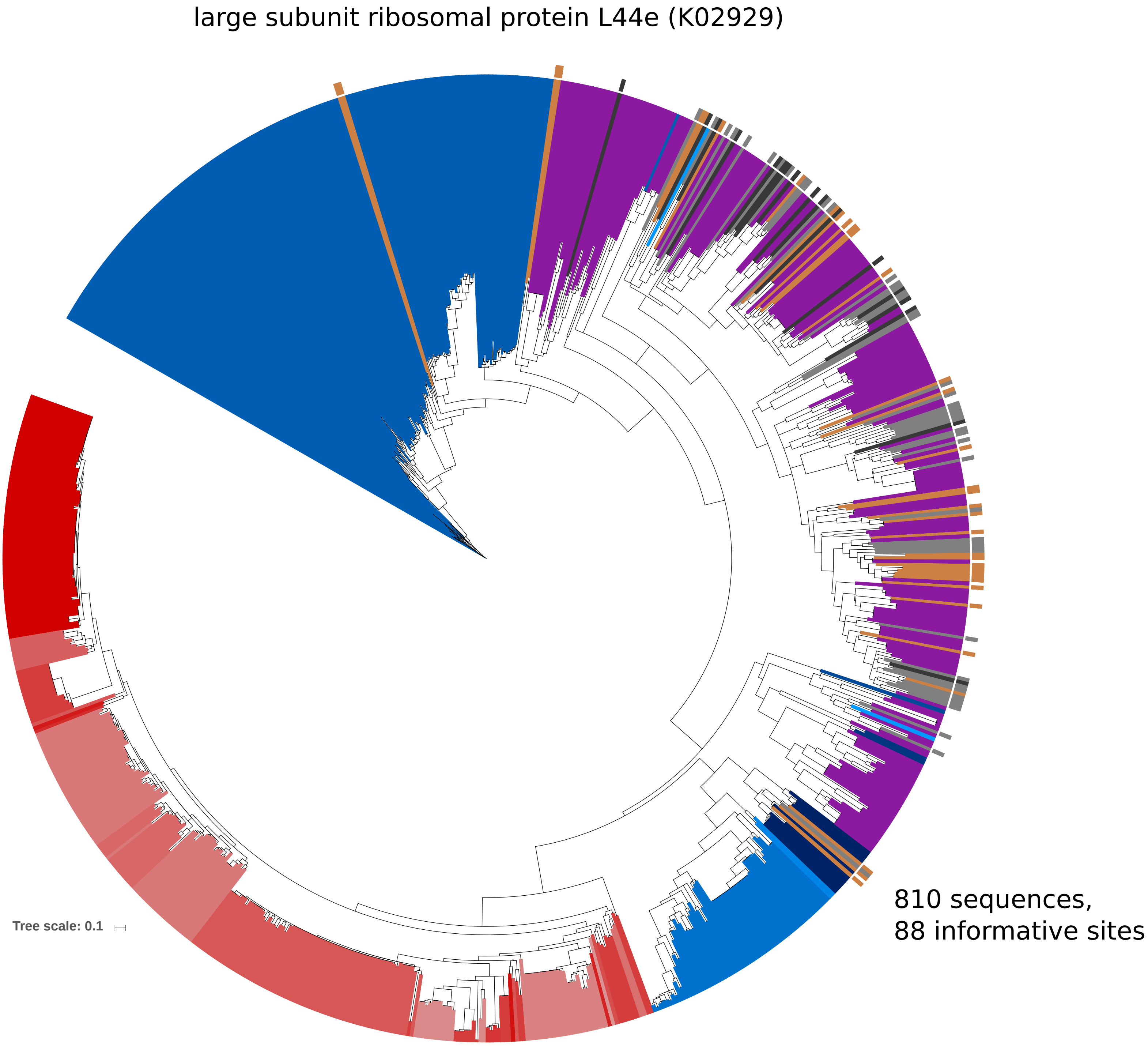
large subunit ribosomal protein L44e (K02929)
Tree scale: 0.1
810 sequences,
88 informative sites

large subunit ribosomal protein L7/L12 (K02935)

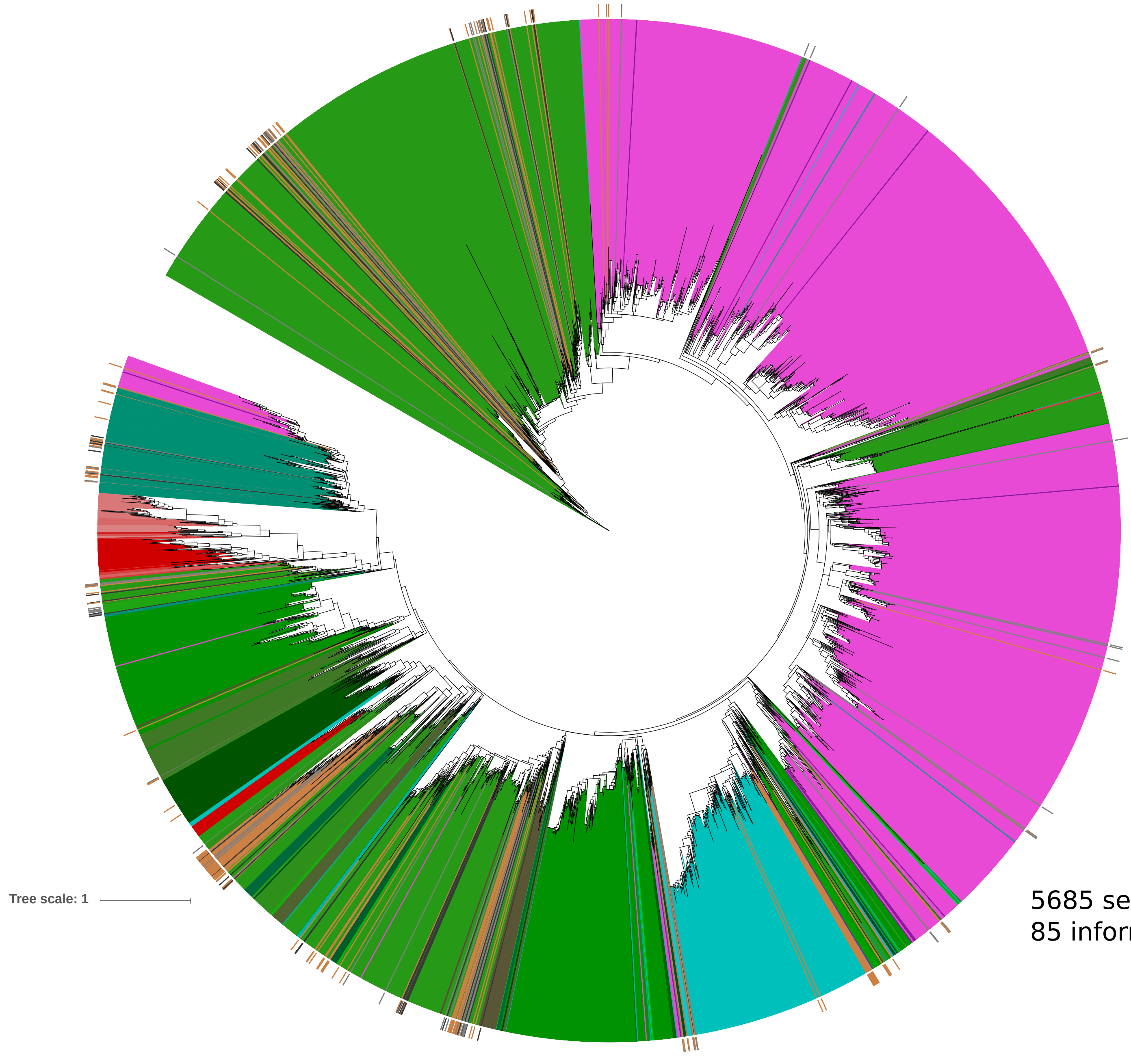

5685 sequences,
85 informative sites

large subunit ribosomal protein LX (K02944)

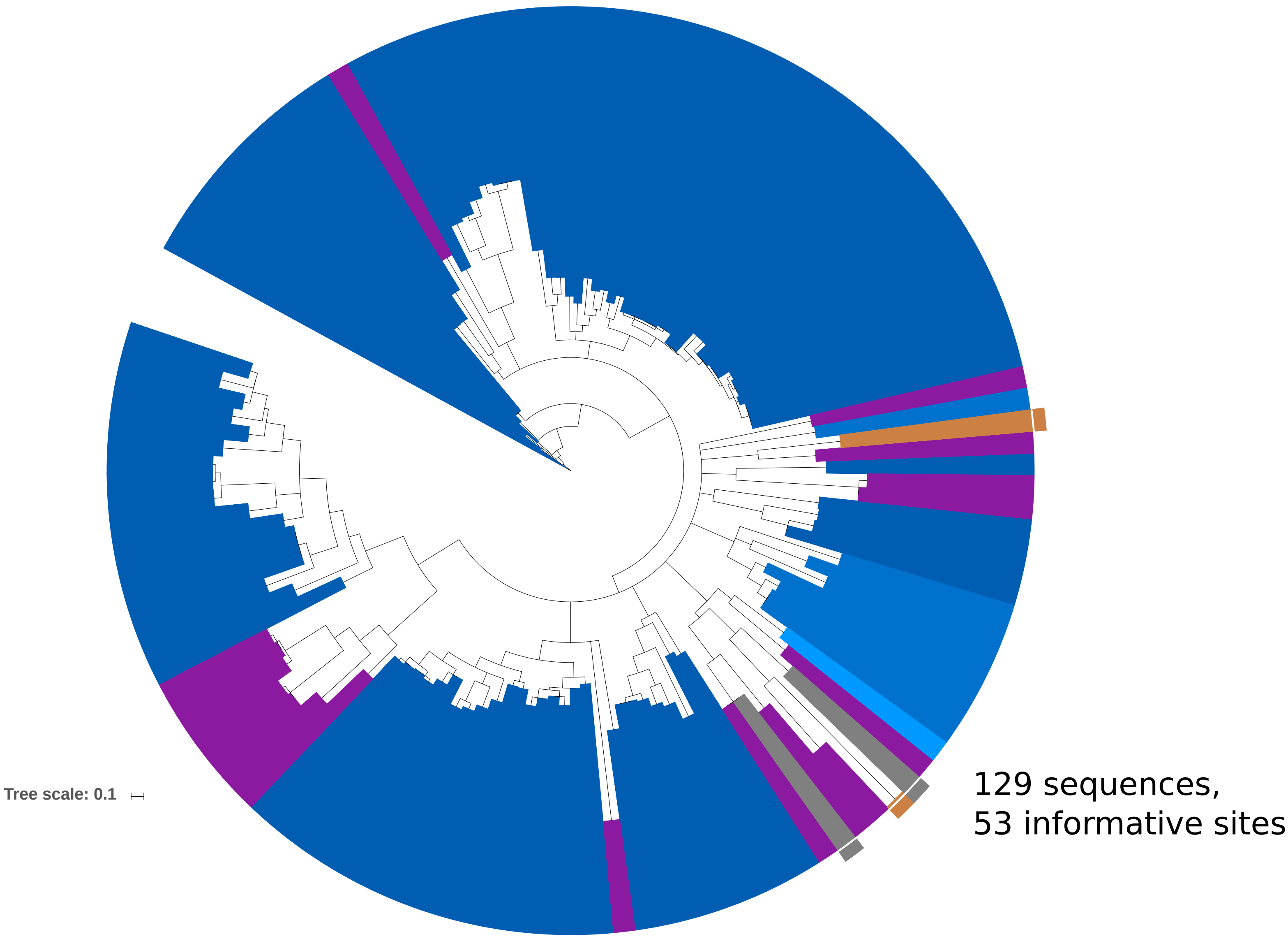

small subunit ribosomal protein S1 (K02945)

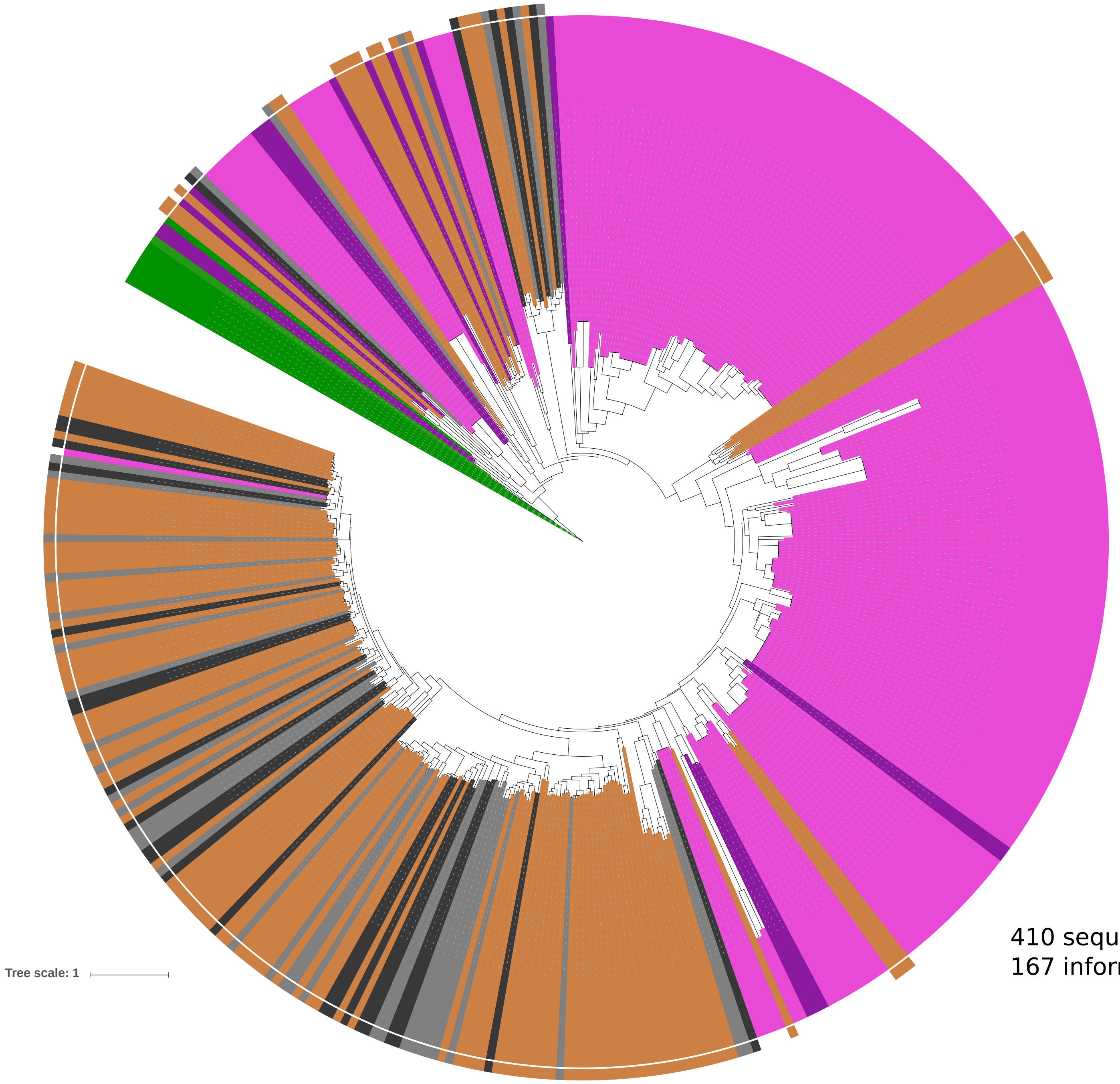

410 sequences,
167 informative sites

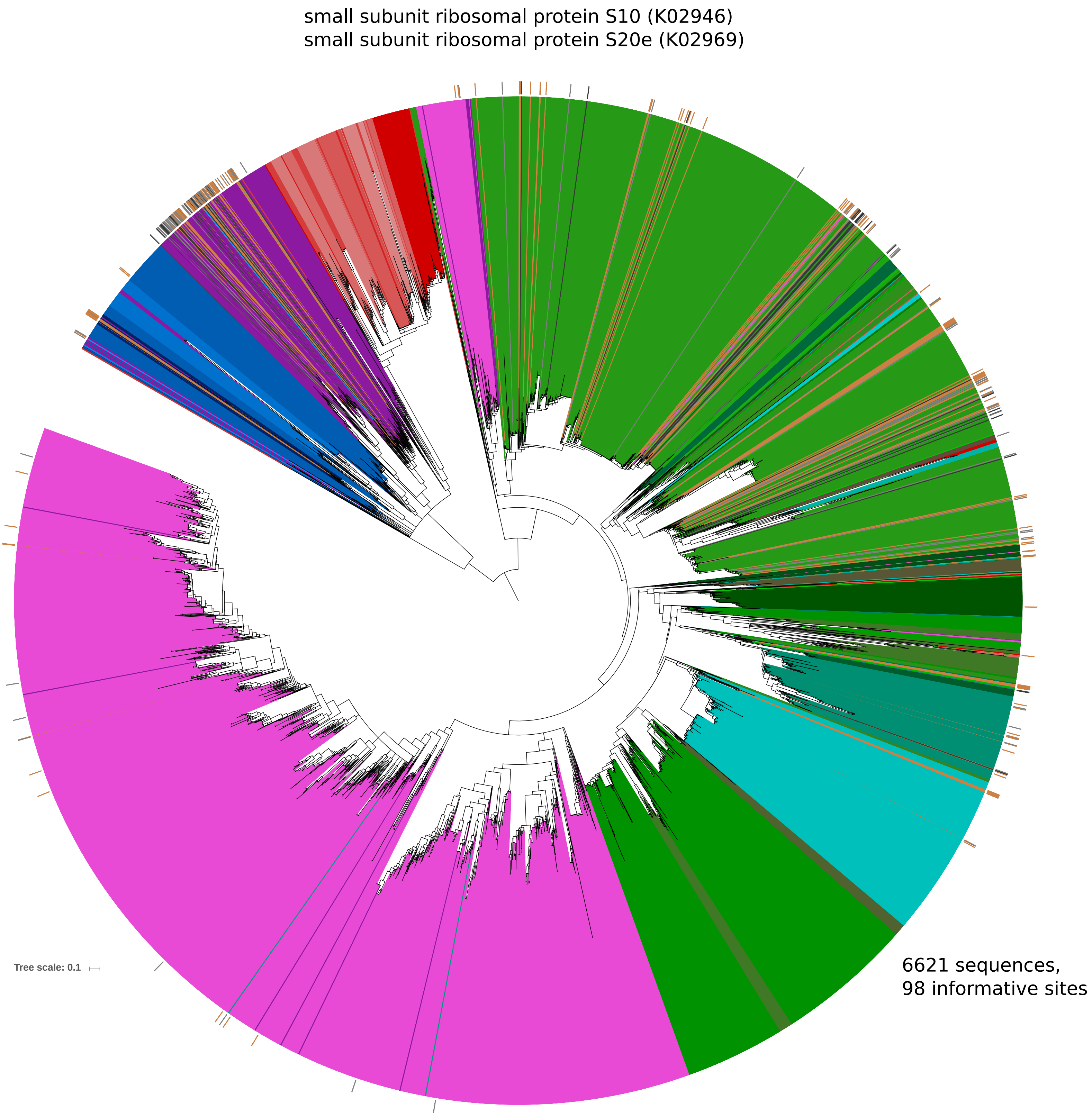
small subunit ribosomal protein S10 (K02946)
small subunit ribosomal protein S20e (K02969)
Tree scale: 0.1
6621 sequences,
98 informative sites

small subunit ribosomal protein S11 (K02948)
small subunit ribosomal protein S14e (K02955)

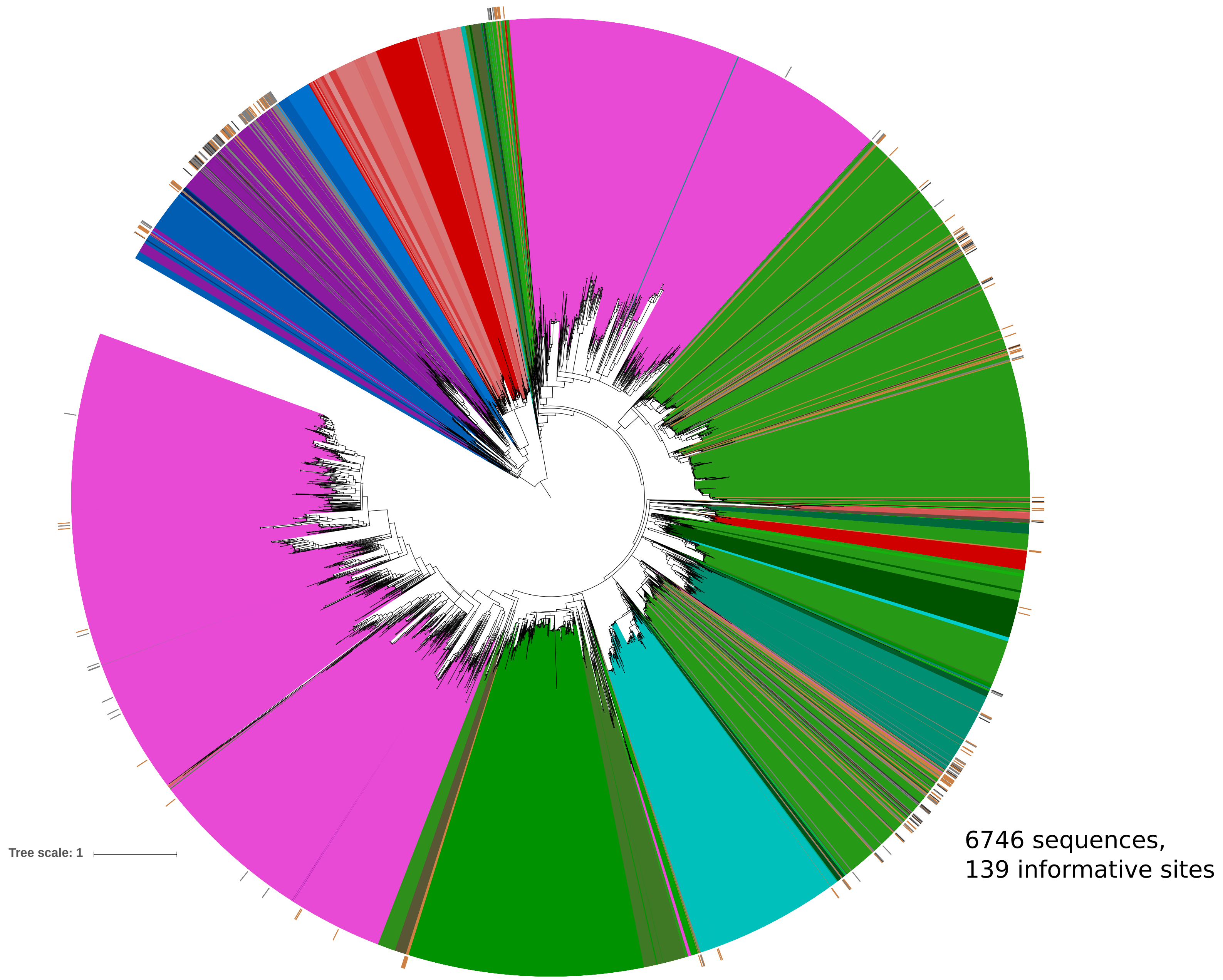

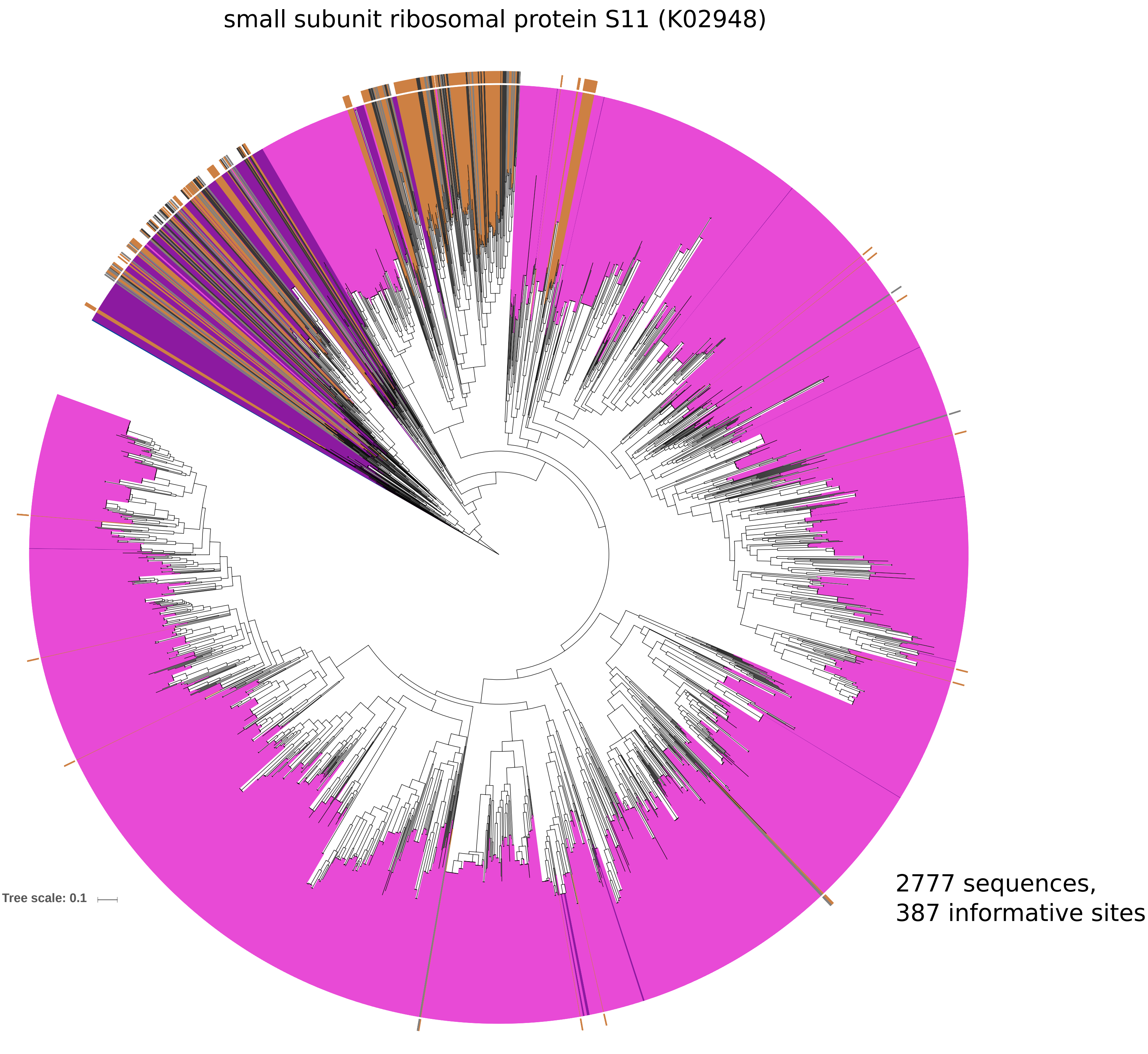
small subunit ribosomal protein S11 (K02948)
Tree scale: 0.1
2777 sequences,
387 informative sites

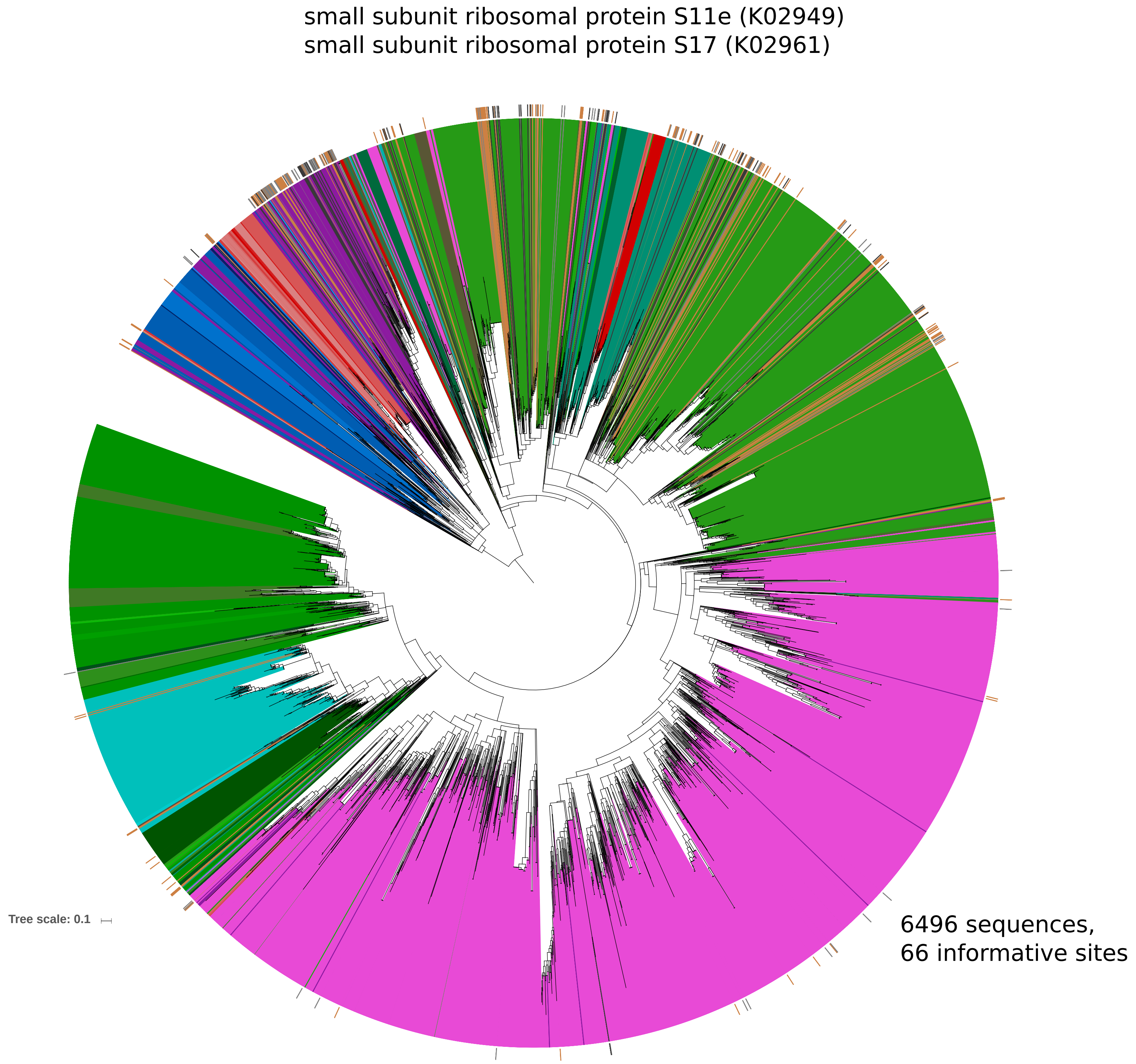
small subunit ribosomal protein S11e (K02949)
small subunit ribosomal protein S17 (K02961)
Tree scale: 0.1
6496 sequences,
66 informative sites

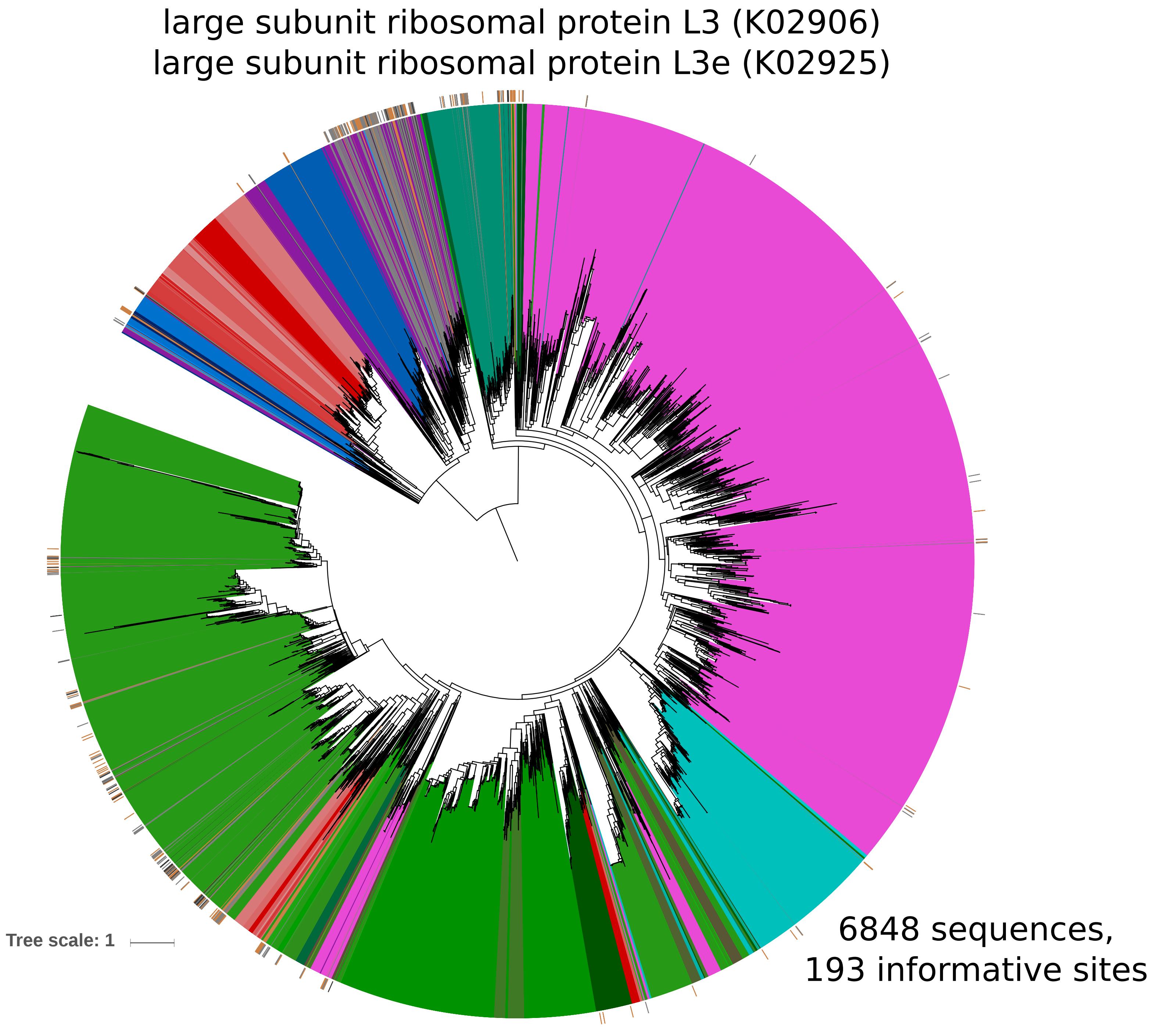
large subunit ribosomal protein L3 (K02906)
large subunit ribosomal protein L3e (K02925)
Tree scale: 1
6848 sequences,
193 informative sites
