# Supplementary material for: Carbon Fixation by Marine Ultrasmall Prokaryotes
Source: Genome Biol Evol. 2019 Mar 23;11(4):1166–77. doi: 10.1093/gbe/evz050 (PMC6475129; doi:10.1093/gbe/evz050)

Color chart

- all
- UO
- WUO
- CPR
- DPANN

- Acidobacteria
- Actinobacteria
- Aquificae
- Armatimonadetes
- Bacteroidetes
- Caldiserica
- Calditrichaeota
- Chlamydiae
- Chlorobi
- Chloroflexi
- Chrysiogenetes
- Coprothermobacterota

- Cyanobacteria
- Deferribacteres
- Dictyoglomi
- Elusimicrobia
- Fibrobacteres
- Firmicutes
- Fusobacteria
- Gemmatimonadetes
- Ignavibacteriae
- Kiritimatiellaeota
- Melainabacteria
- NC10
- Nitrospirae
- Planctomycetes
- Proteobacteria
- Spirochaetes
- Synergistetes
- Tenericutes
- Thermodesulfobacteria
- Thermotogae
- Thermus
- Verrucomicrobia

- Annelida
- Apicomplexa
- Arthropoda
- Ascomycota
- Bacillariophyta
- Basidiomycota
- Chlorophyta
- Chordata
- Cnidaria
- Eukaryota
- Microsporidia
- Mollusca
- Nematoda
- Placozoa
- Platyhelminthes
- Streptophyta

- Bathyarchaeota
- Crenarchaeota
- Euryarchaeota
- Korarchaeota
- Lokiarchaeota
- Thaumarchaeota

2-oxoglutarate/2-oxoacid ferredoxin oxidoreductase subunit alpha (K00174)

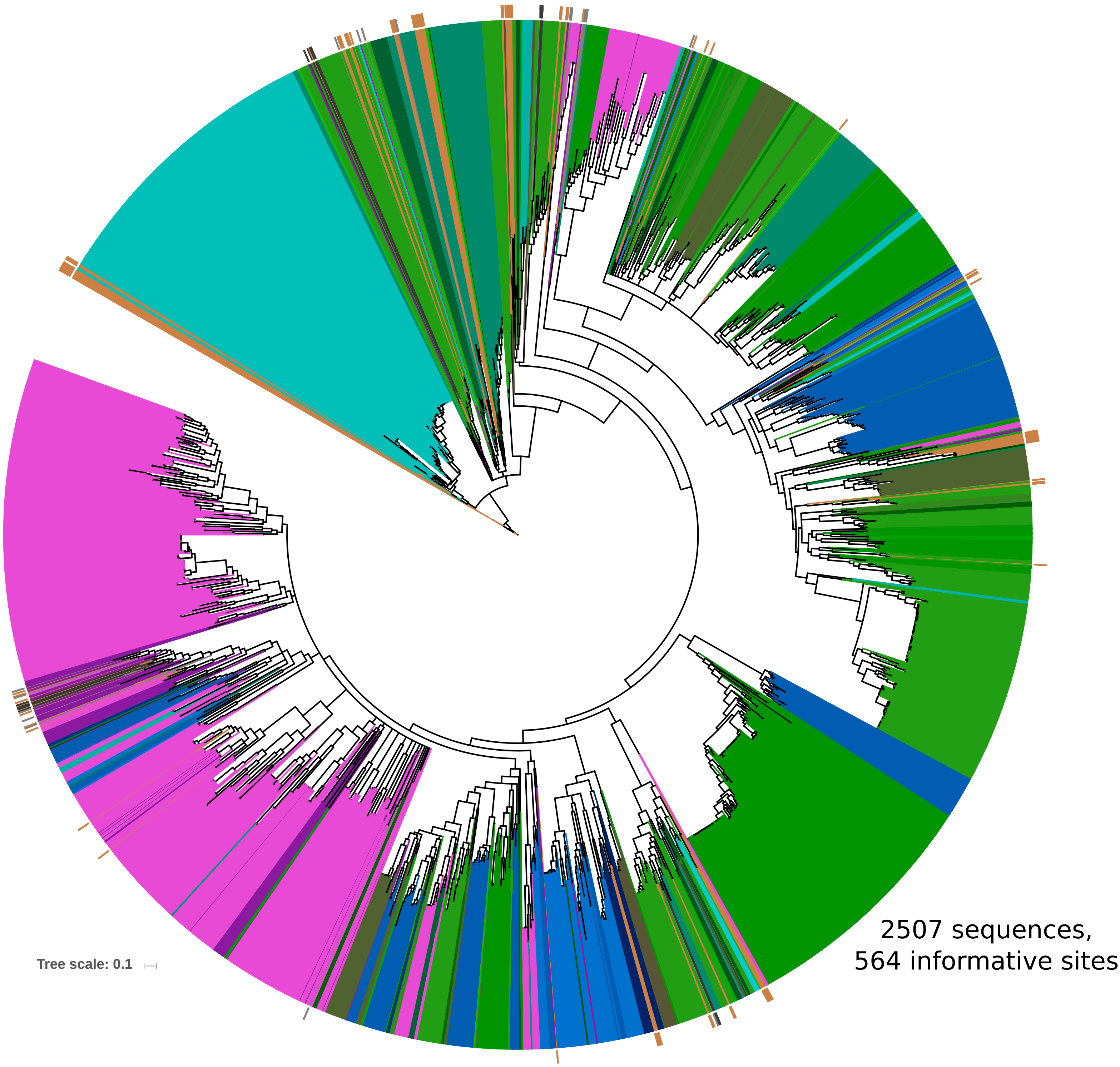

2-oxoglutarate/2-oxoacid ferredoxin oxidoreductase subunit beta (K00175)

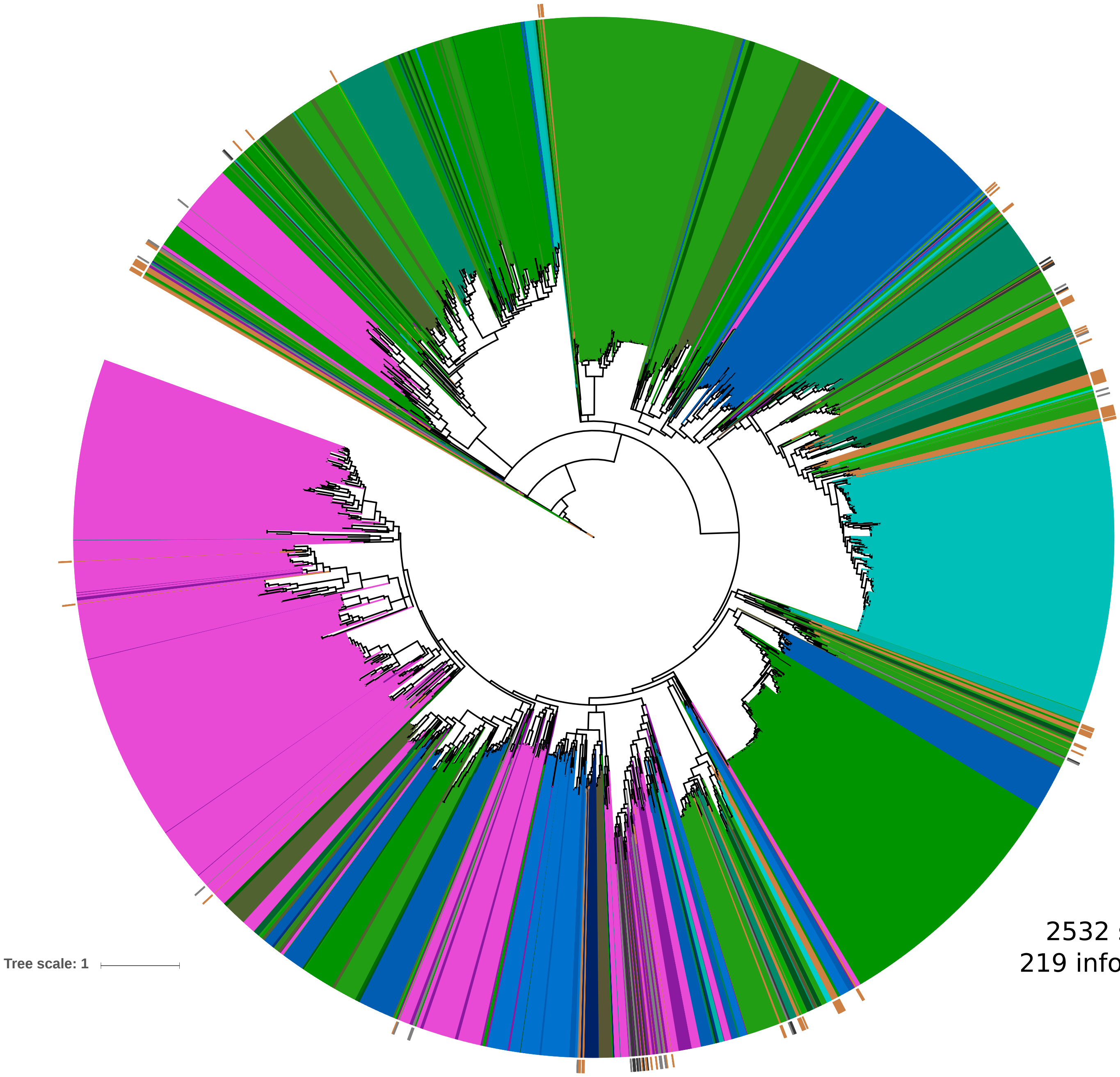

2532 sequences,
219 informative sites

acetyl-CoA decarbonylase/synthase complex subunit delta (K00194)

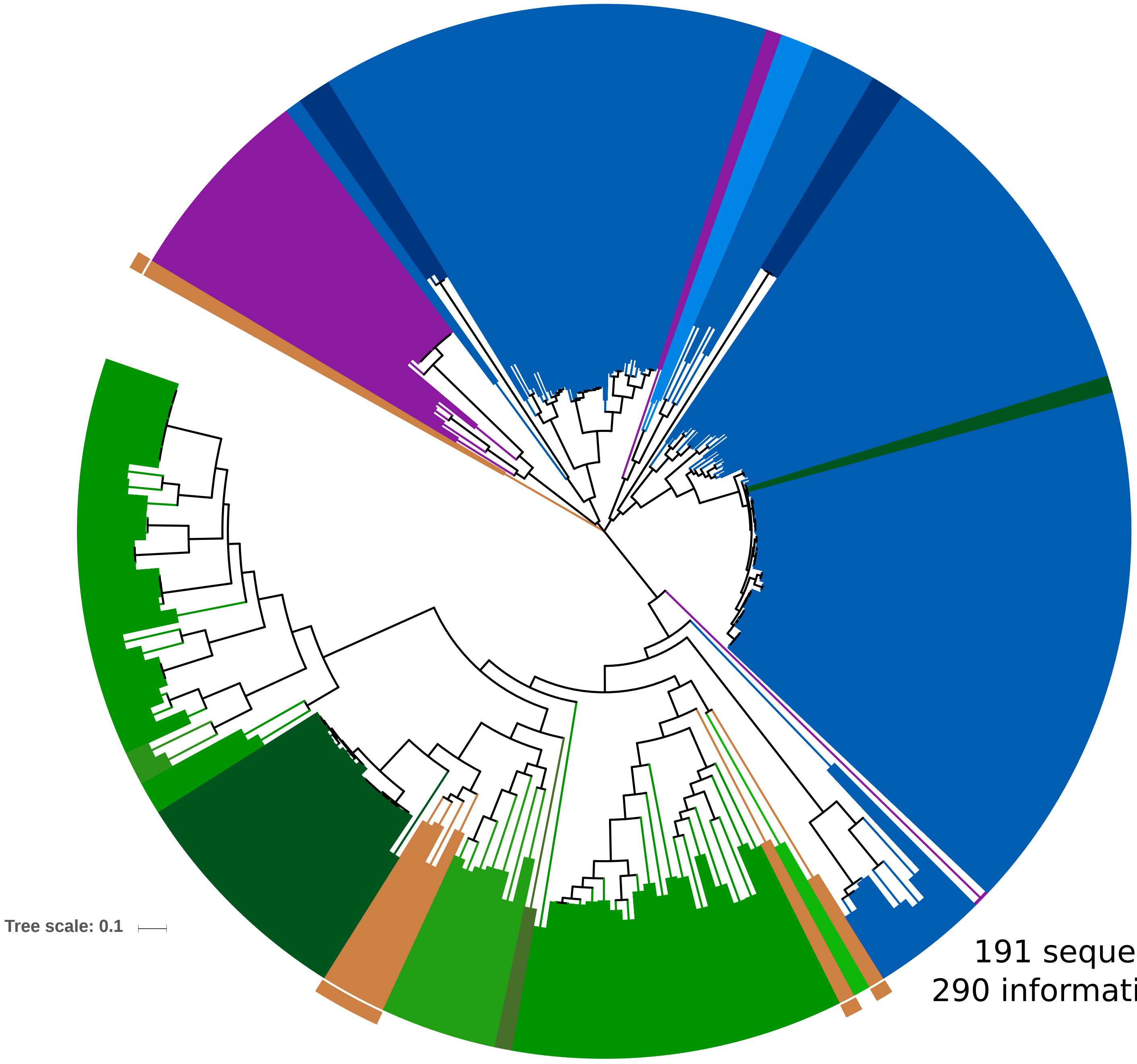

191 sequences,
290 informative sites

acetyl-CoA decarbonylase/synthase complex subunit gamma (K00197)

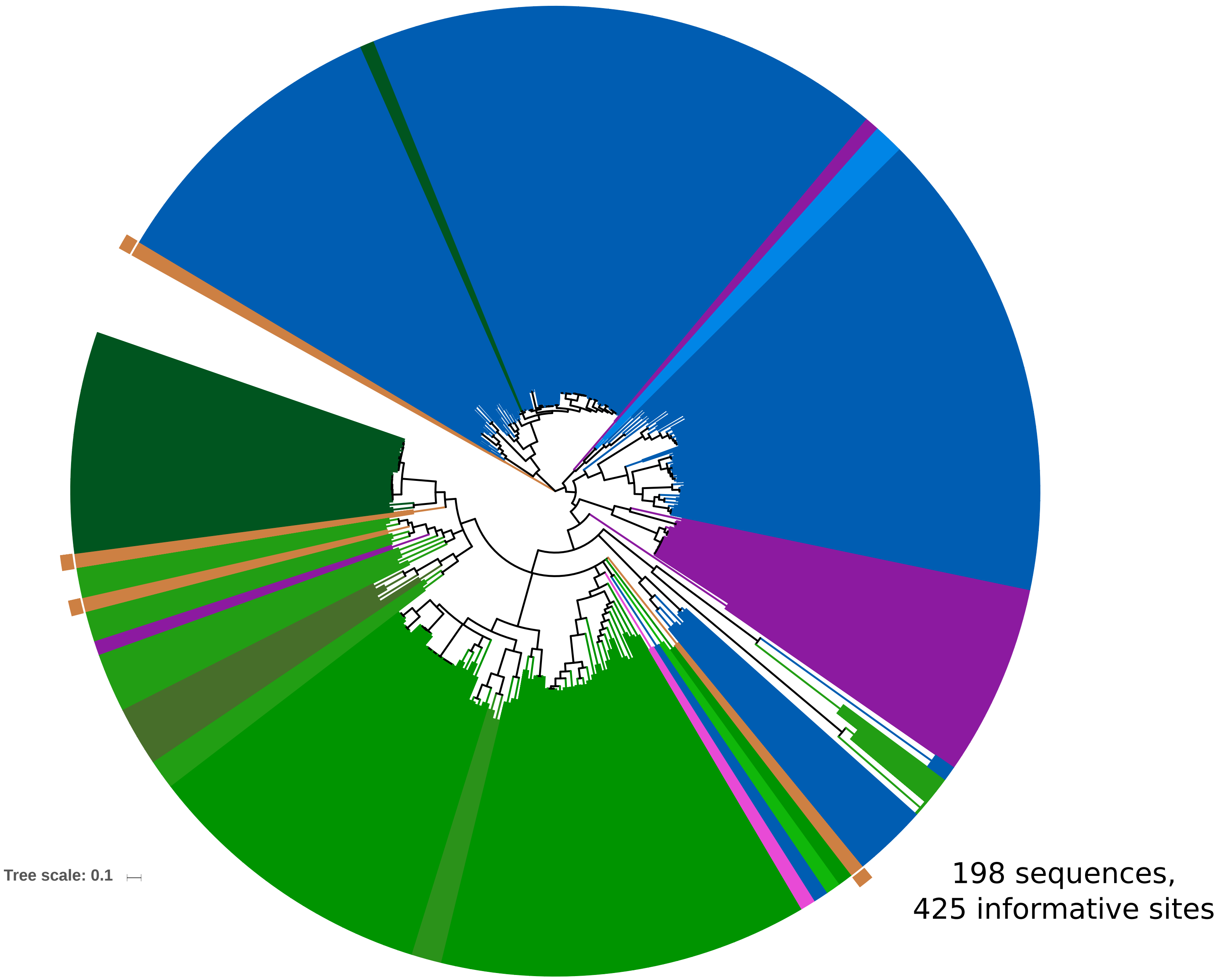

anaerobic carbon-monoxide dehydrogenase catalytic subunit (K00198)

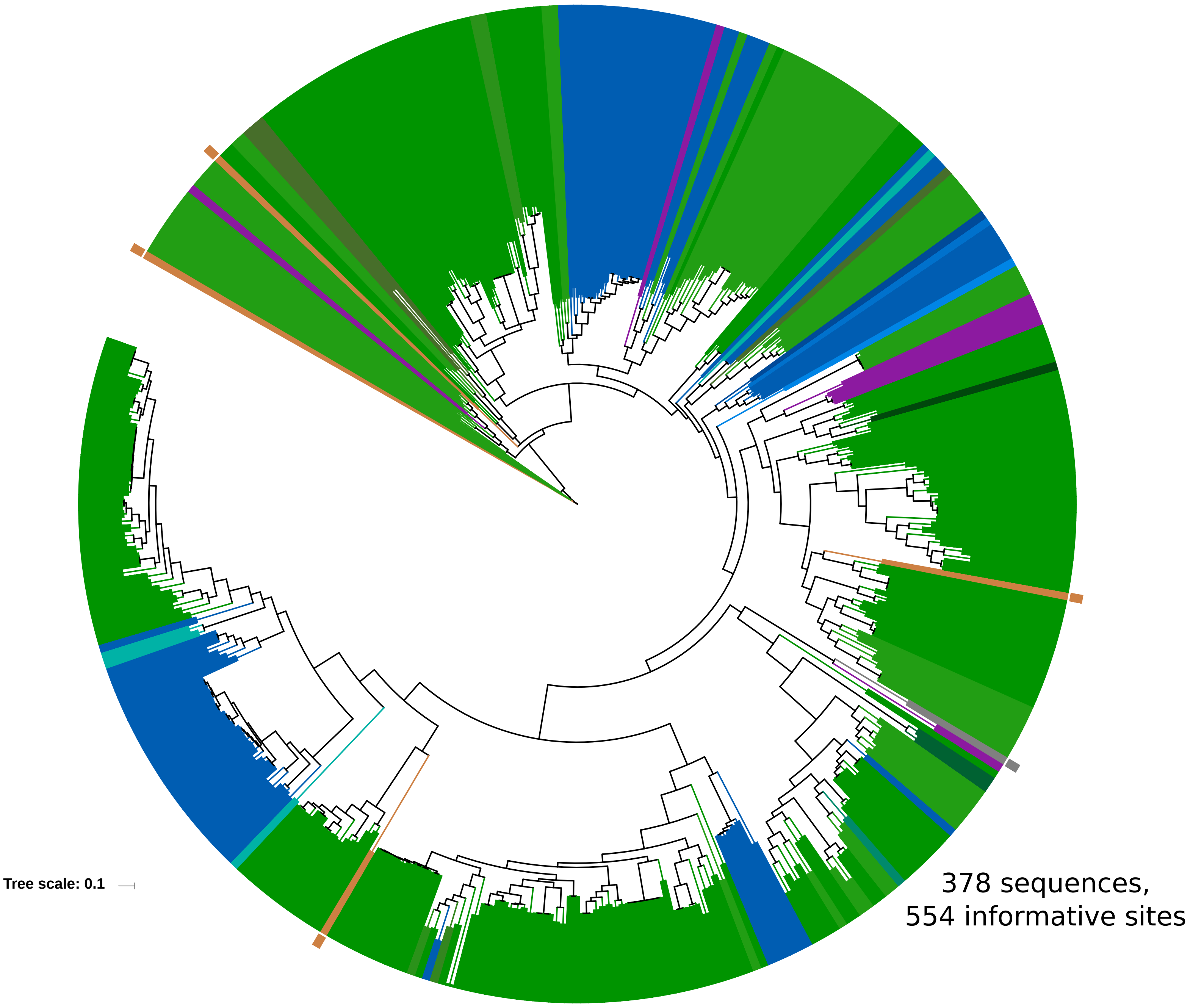

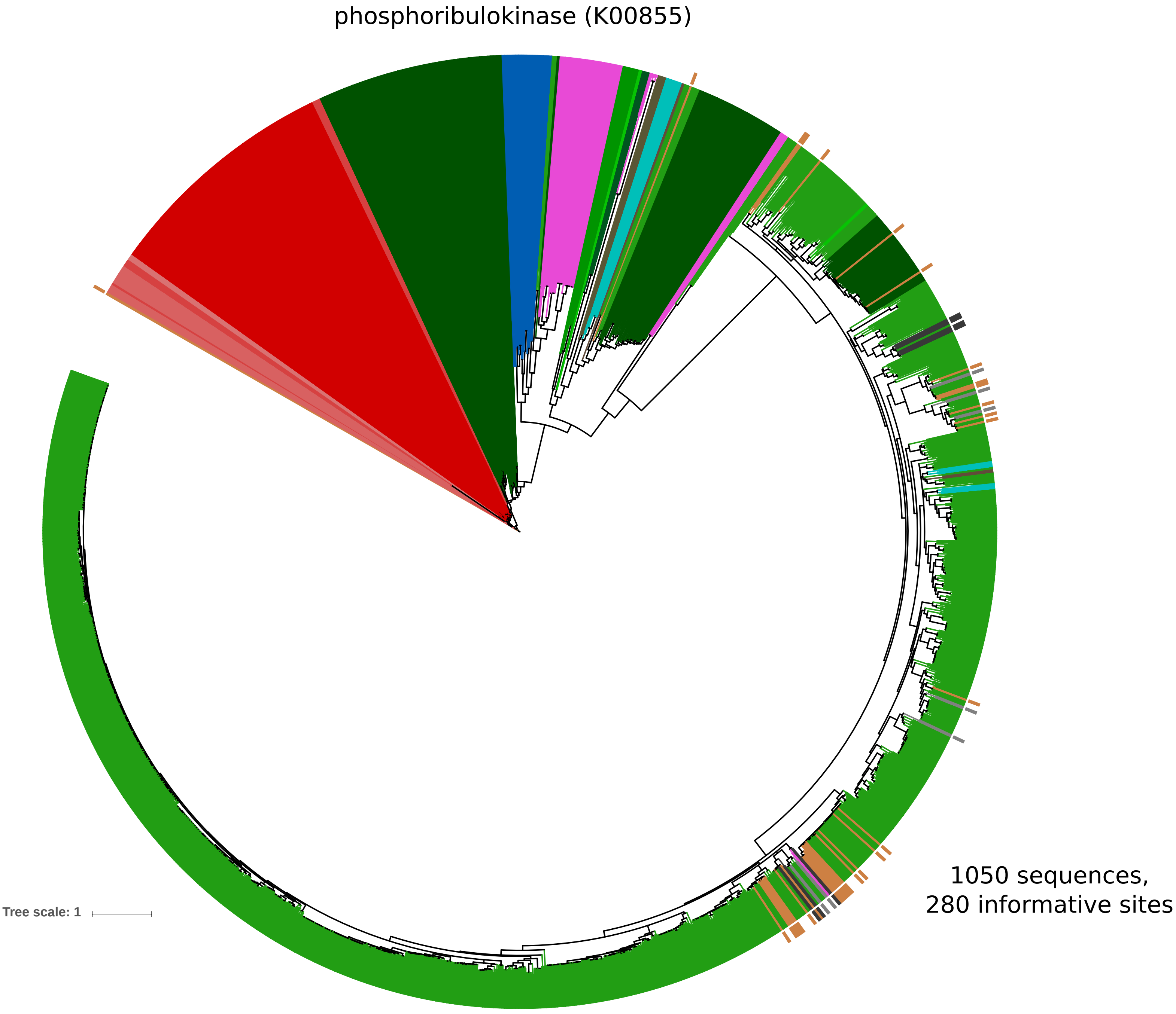
phosphoribulokinase (K00855)
1050 sequences,
280 informative sites
Tree scale: 1

methylenetetrahydrofolate dehydrogenase (NADP+) / methenyltetrahydrofolate cyclohydrolase (K01491)

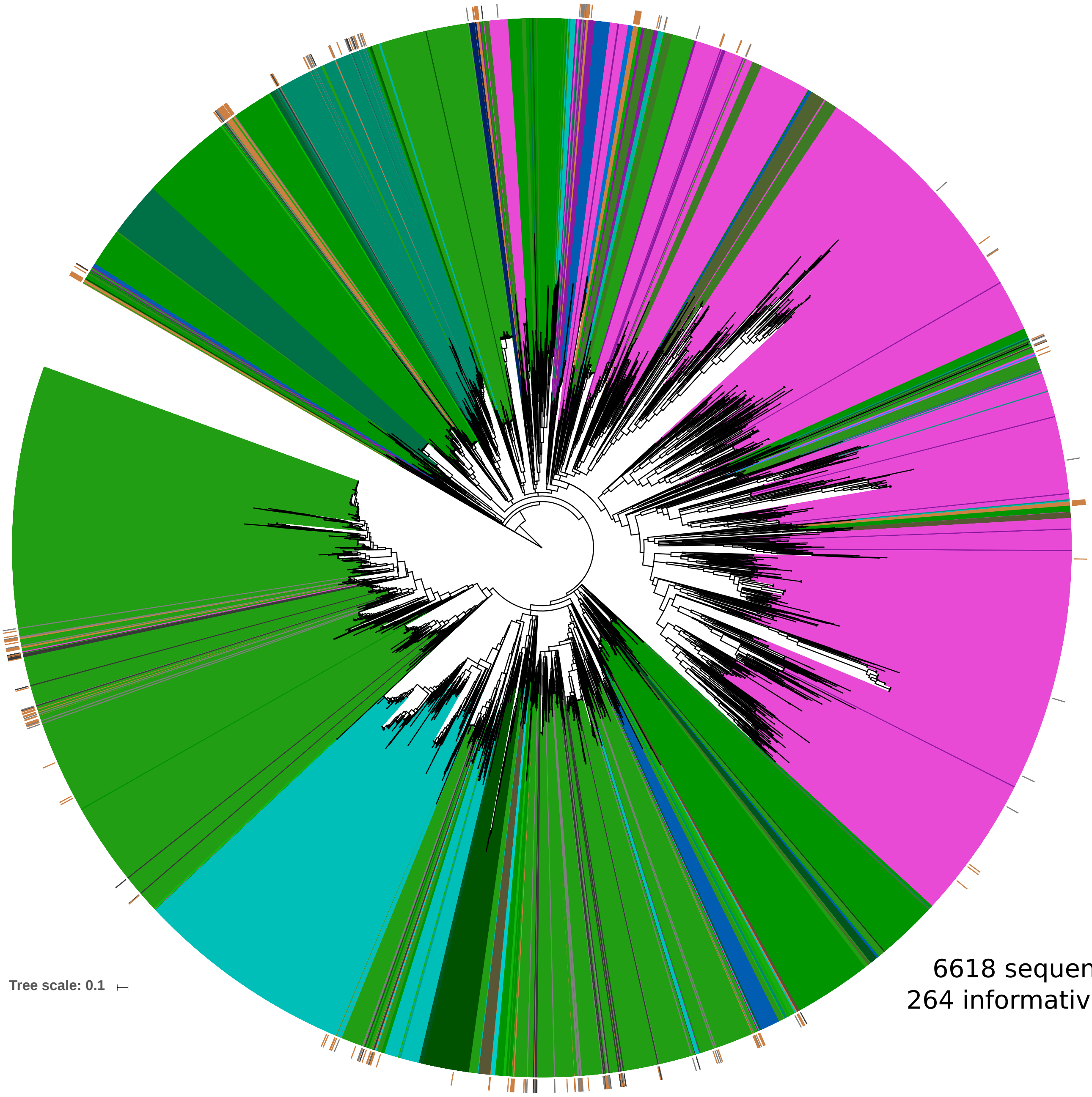

6618 sequences,
264 informative sites

acetyl-CoA/propionyl-CoA carboxylase (K15039)

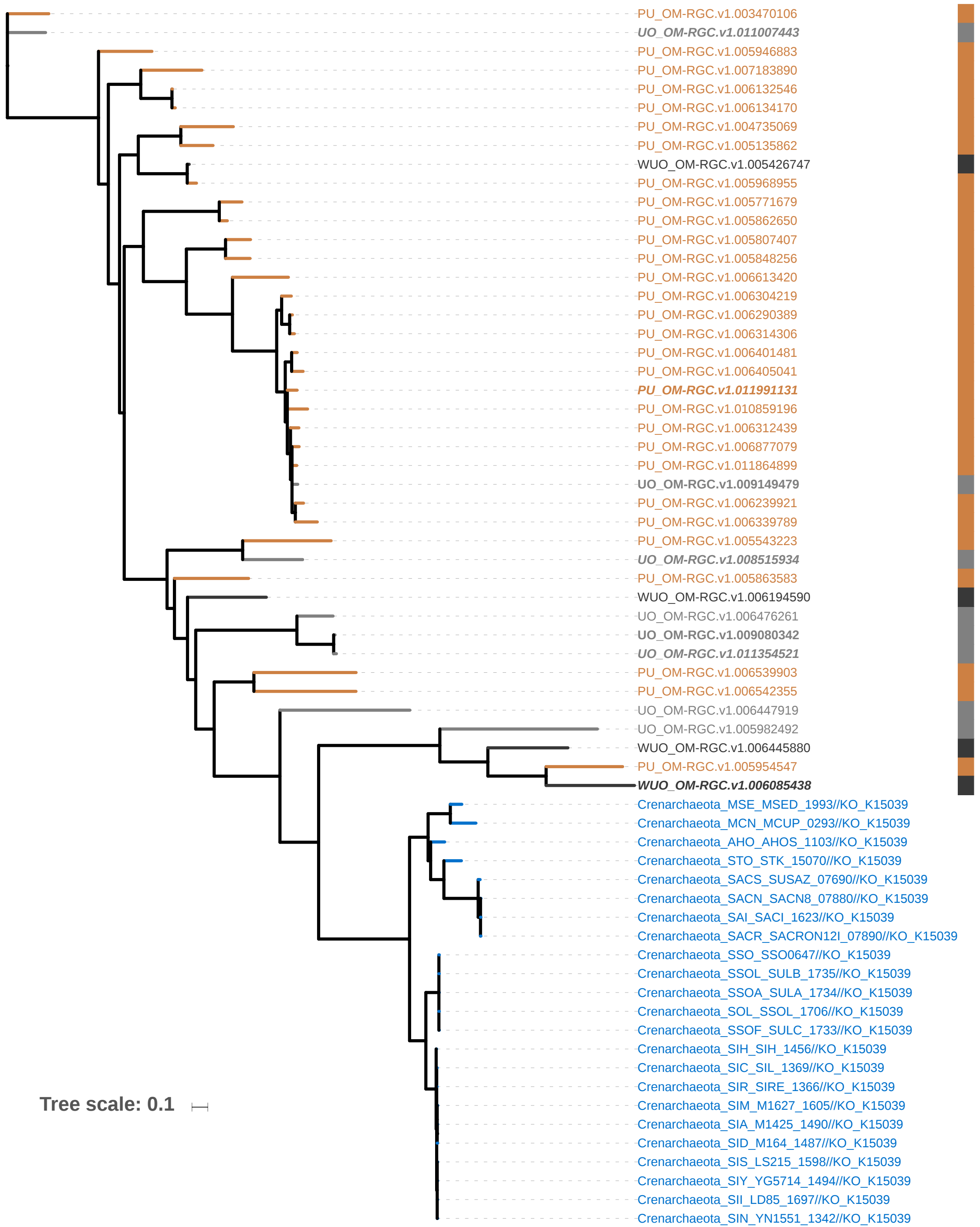

65 sequences,
279 informative sites

ribulose-bisphosphate carboxylase large chain (K01601)

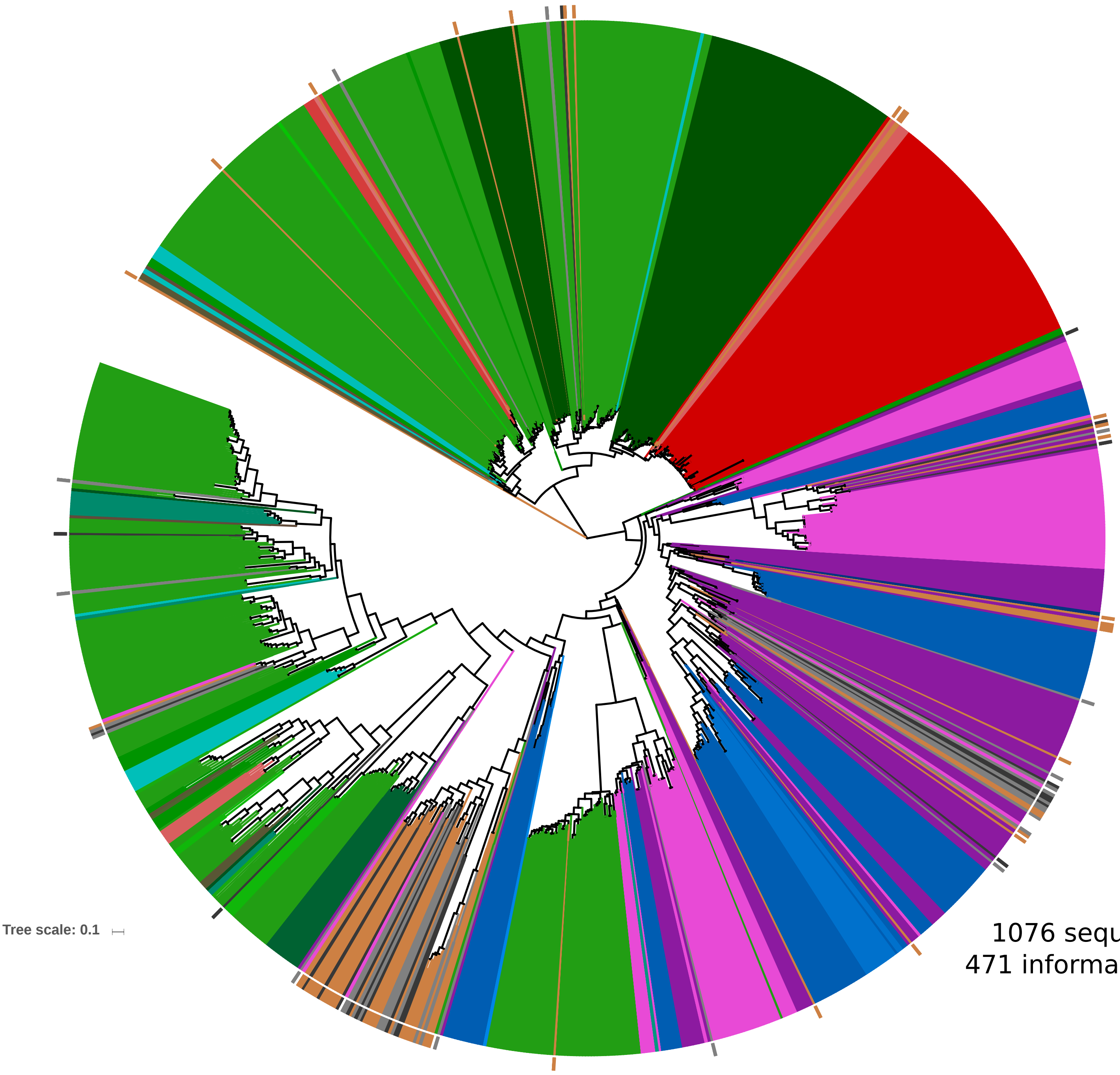

1076 sequences,
471 informative sites

ribulose-bisphosphate carboxylase small chain (K01602)

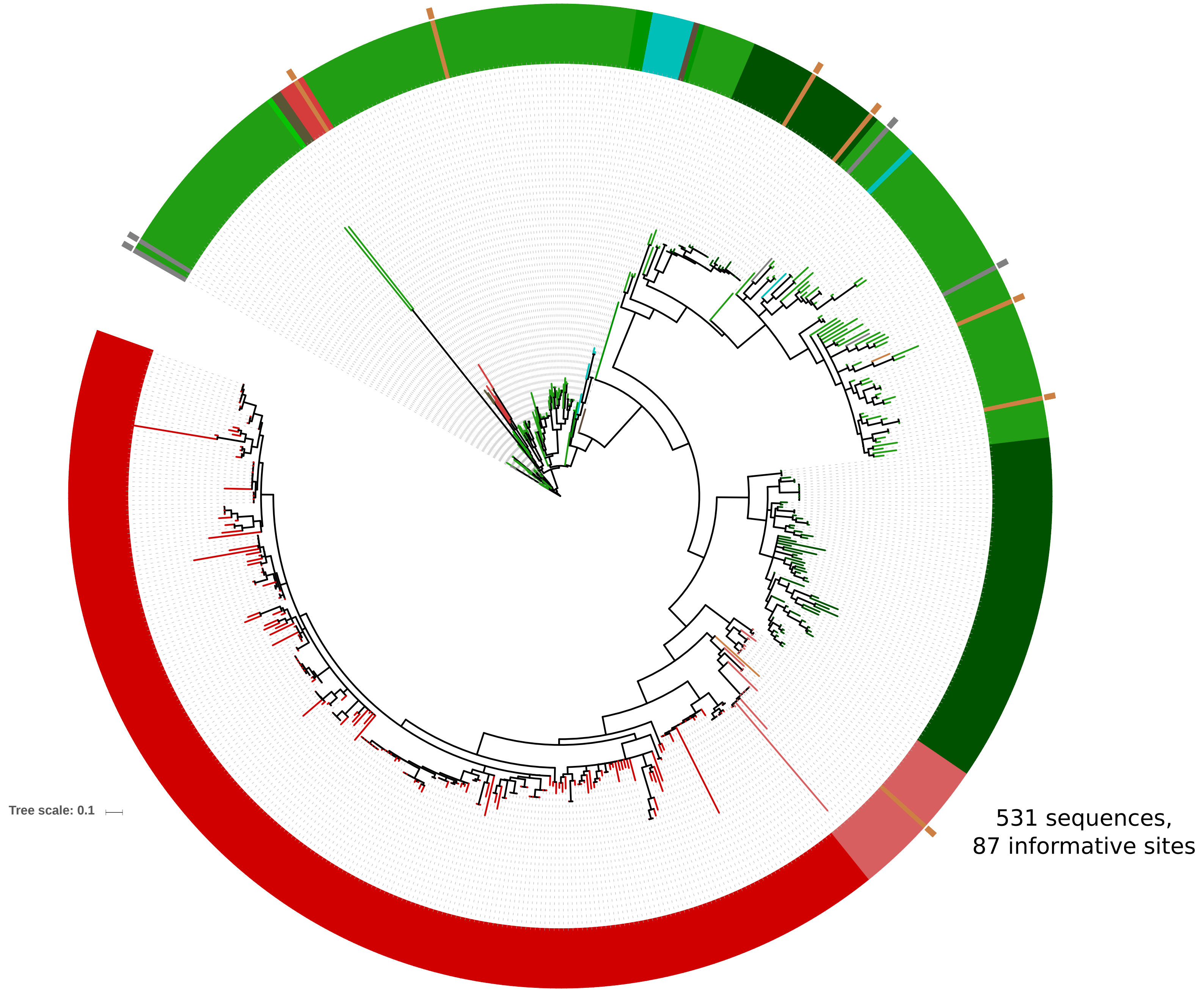

methylmalonyl-CoA mutase, N-terminal domain (K01848)

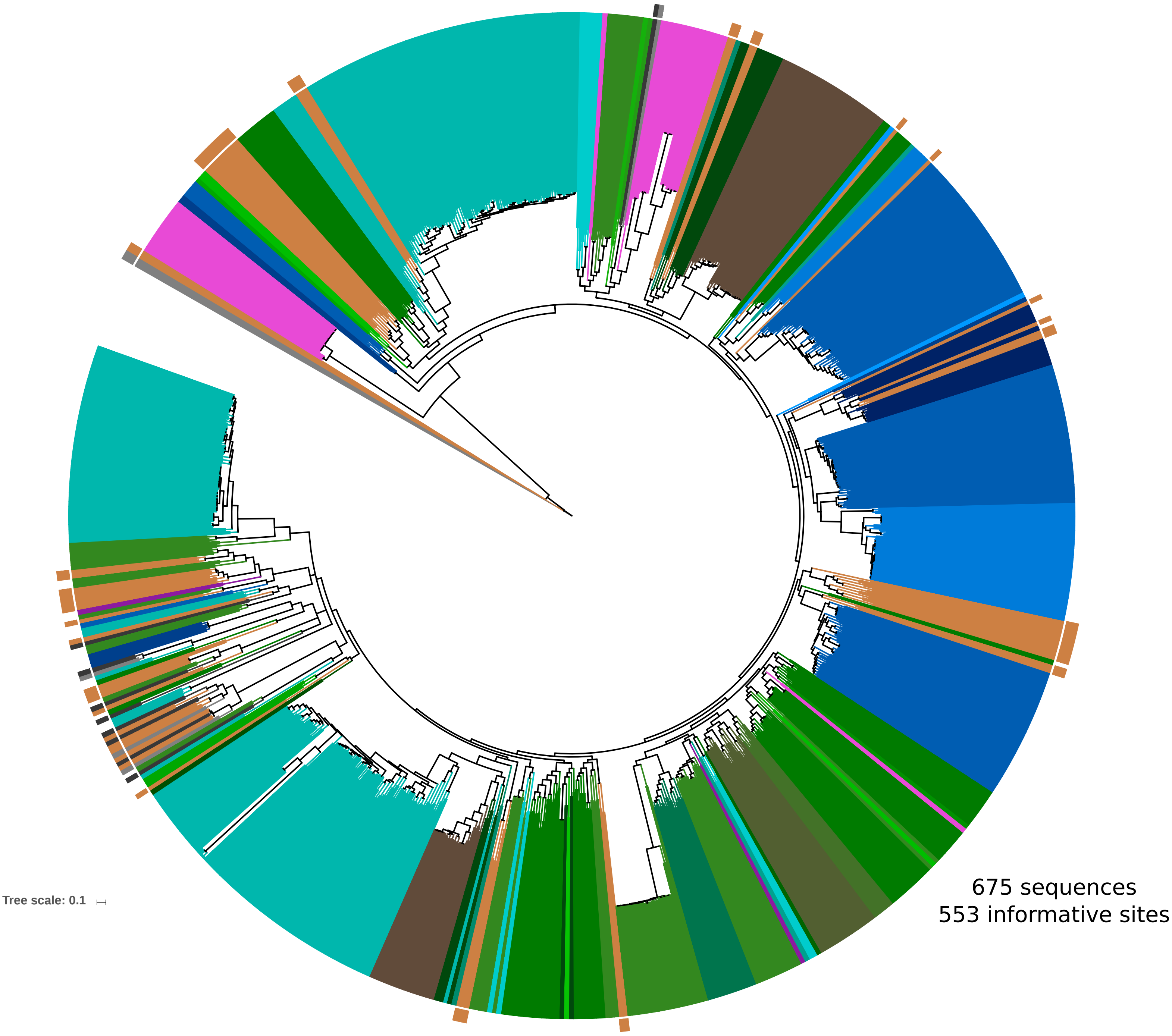

methylmalonyl-CoA mutase, C-terminal domain (K01849)

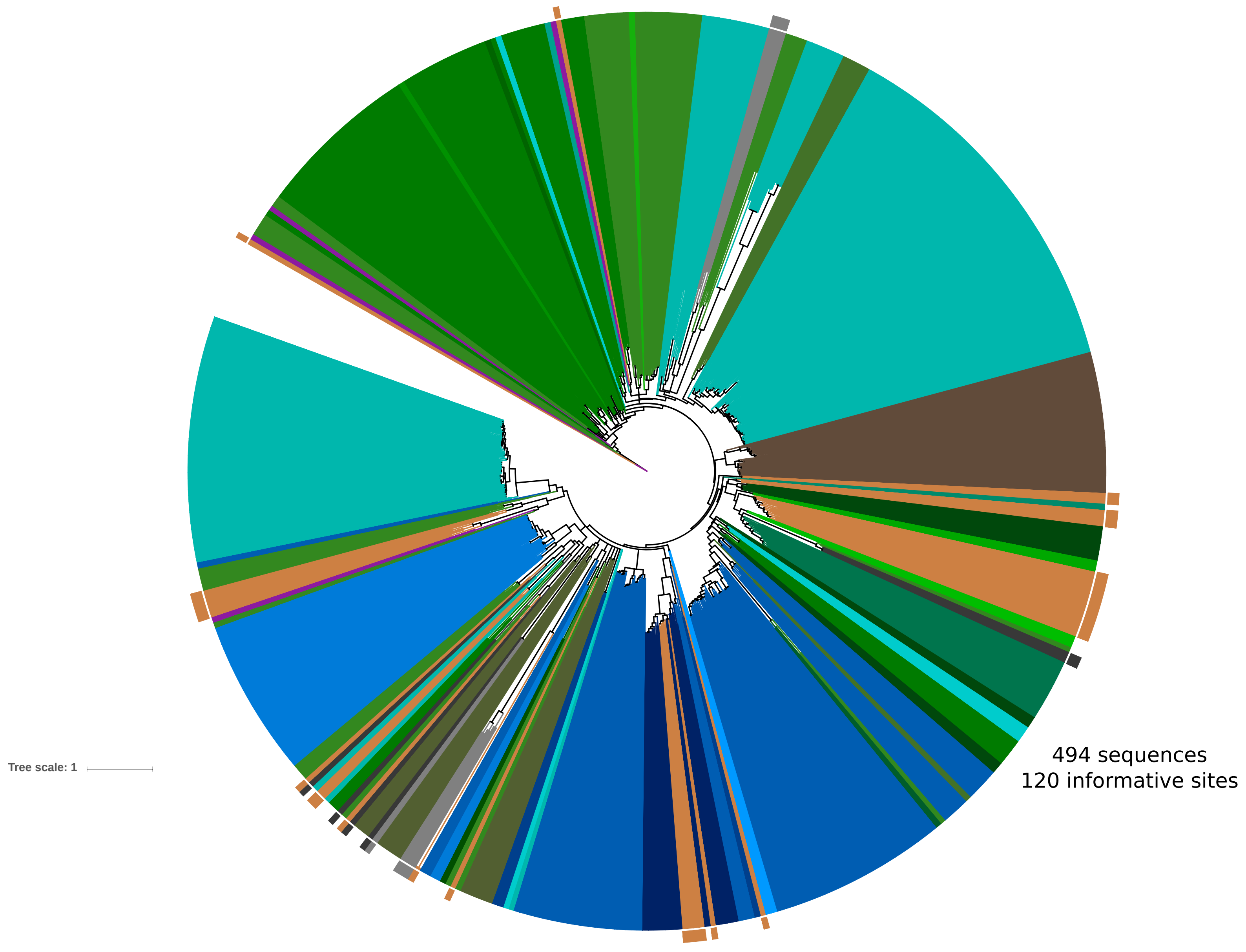

formate--tetrahydrofolate ligase (K01938)

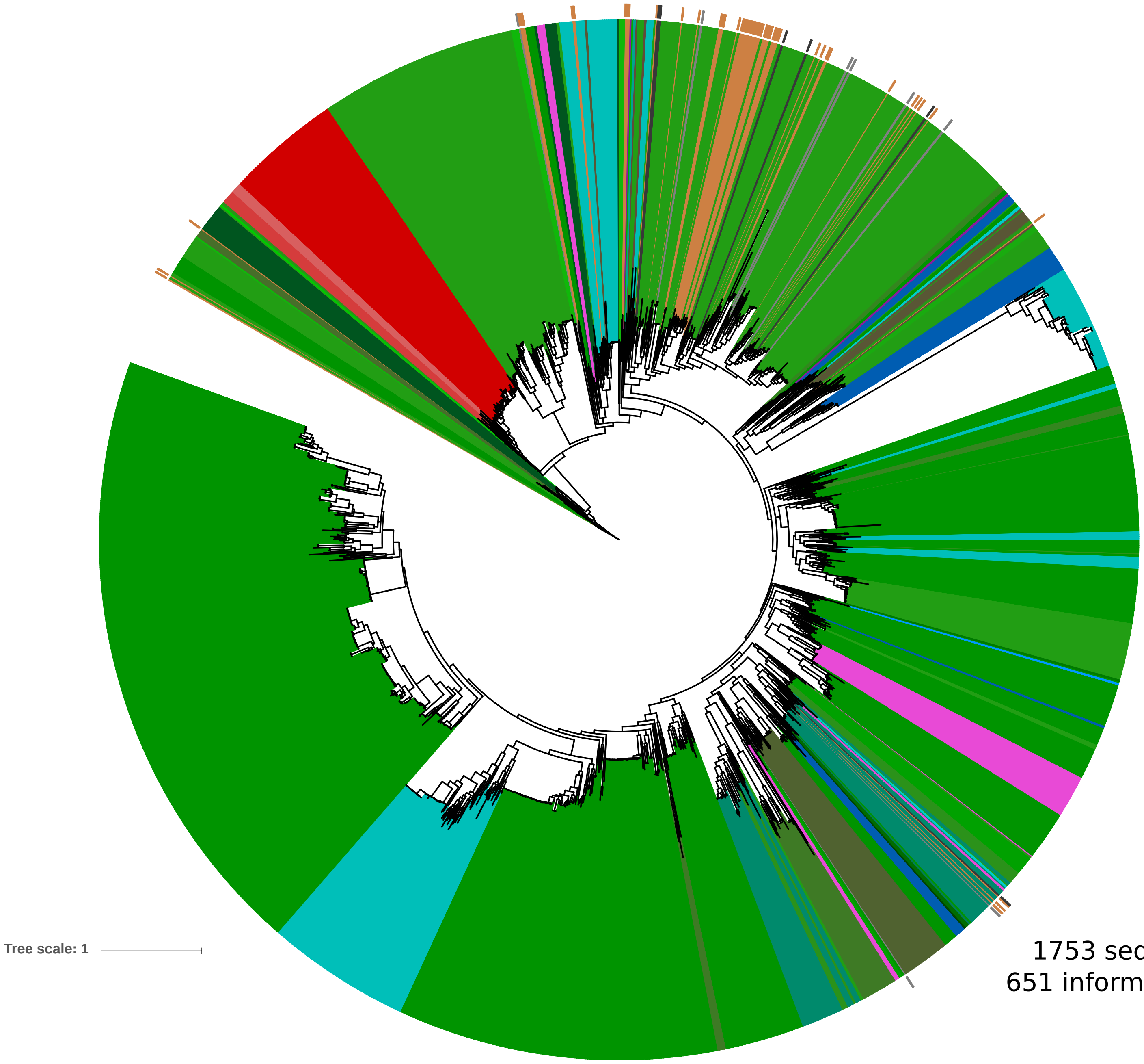

1753 sequences,
651 informative sites

malyl-CoA/(S)-citramalyl-CoA lyase (K08691)

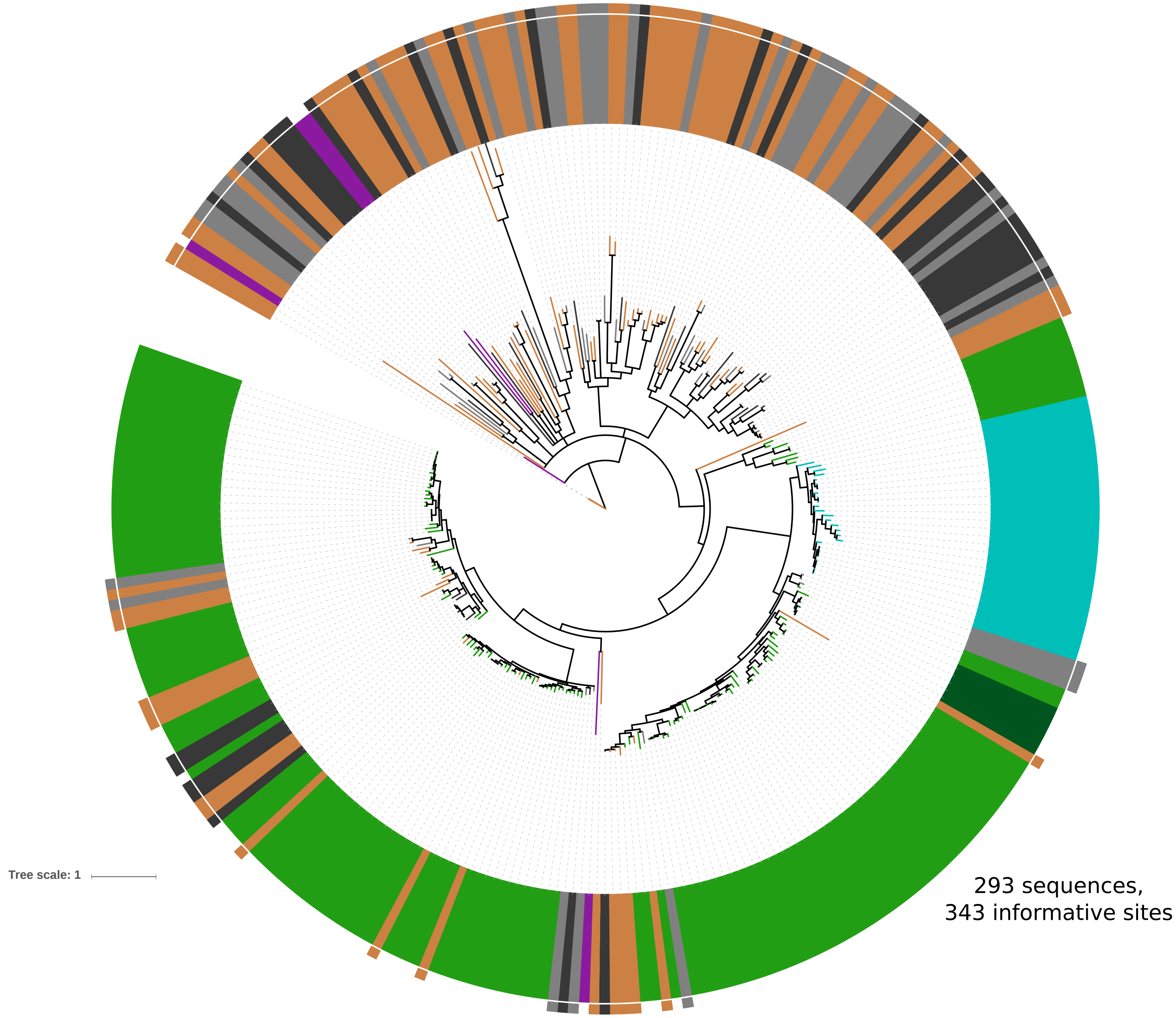

acetyl-CoA synthase (K14138)

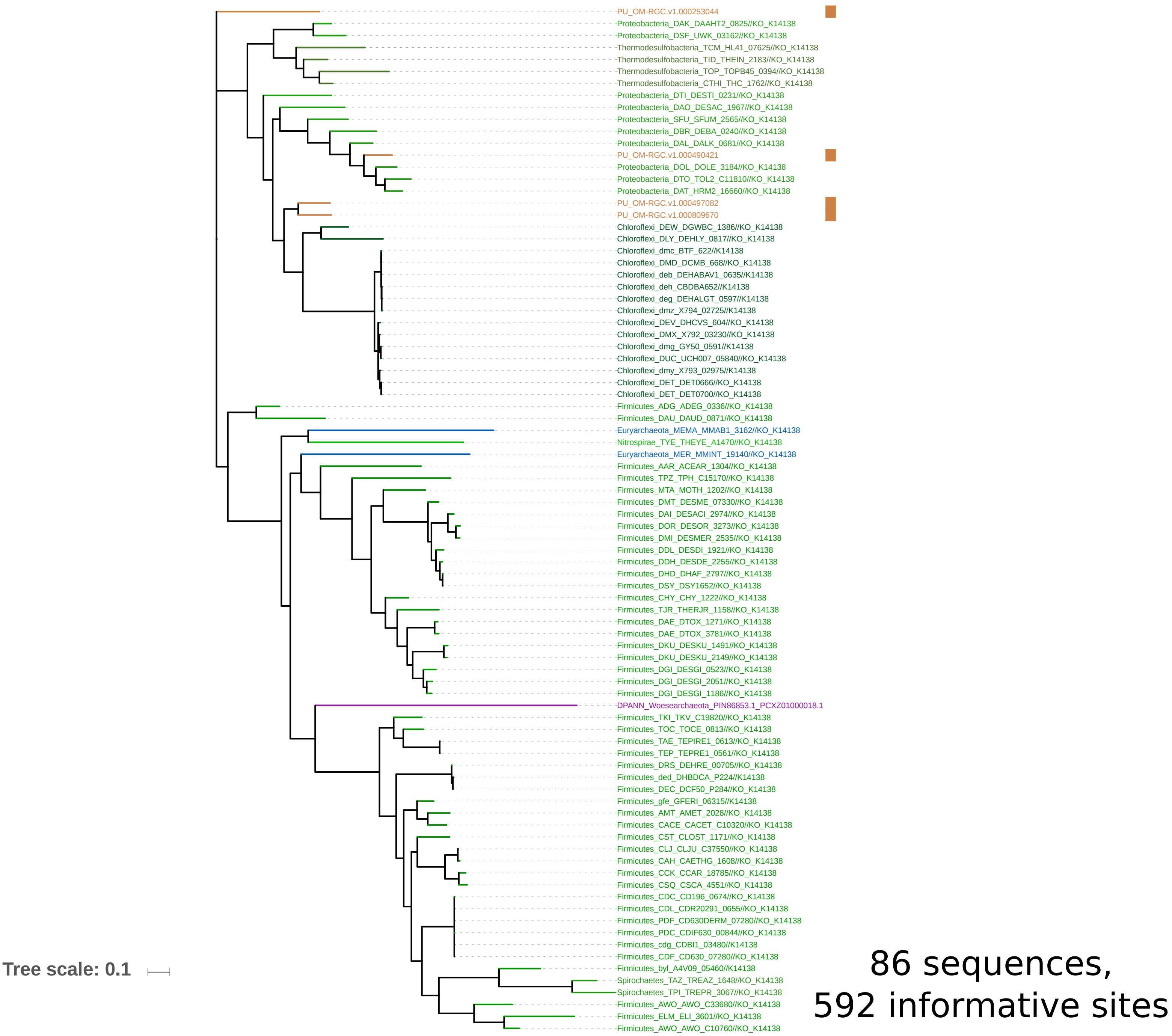

acrylyl-CoA reductase (NADPH) / 3-hydroxypropionyl-CoA dehydratase / 3-hydroxypropionyl-CoA synthetase (K14469)

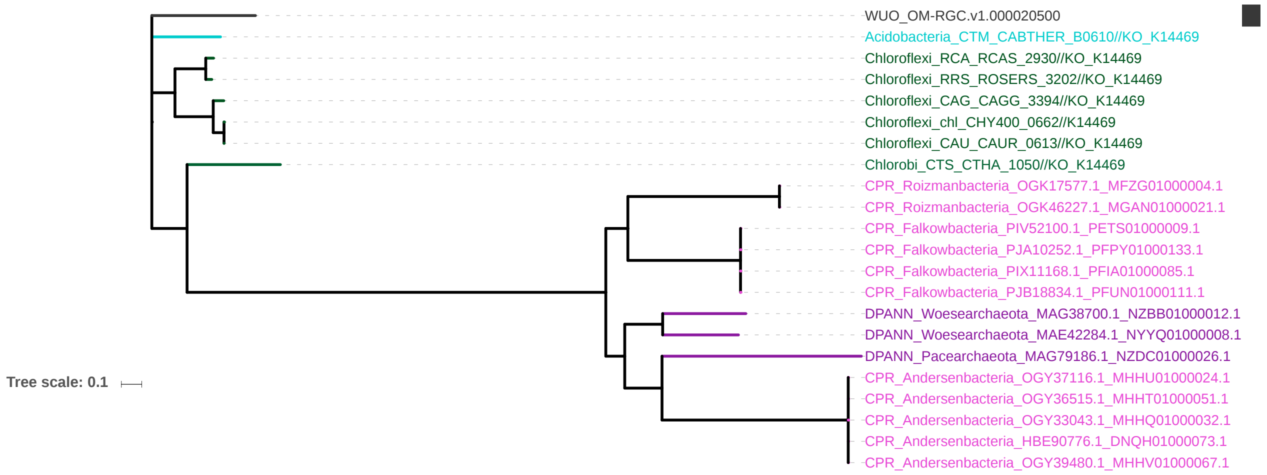

22 sequences,
924 informative sites

malonyl-CoA/succinyl-CoA reductase (NADPH) (K15017)

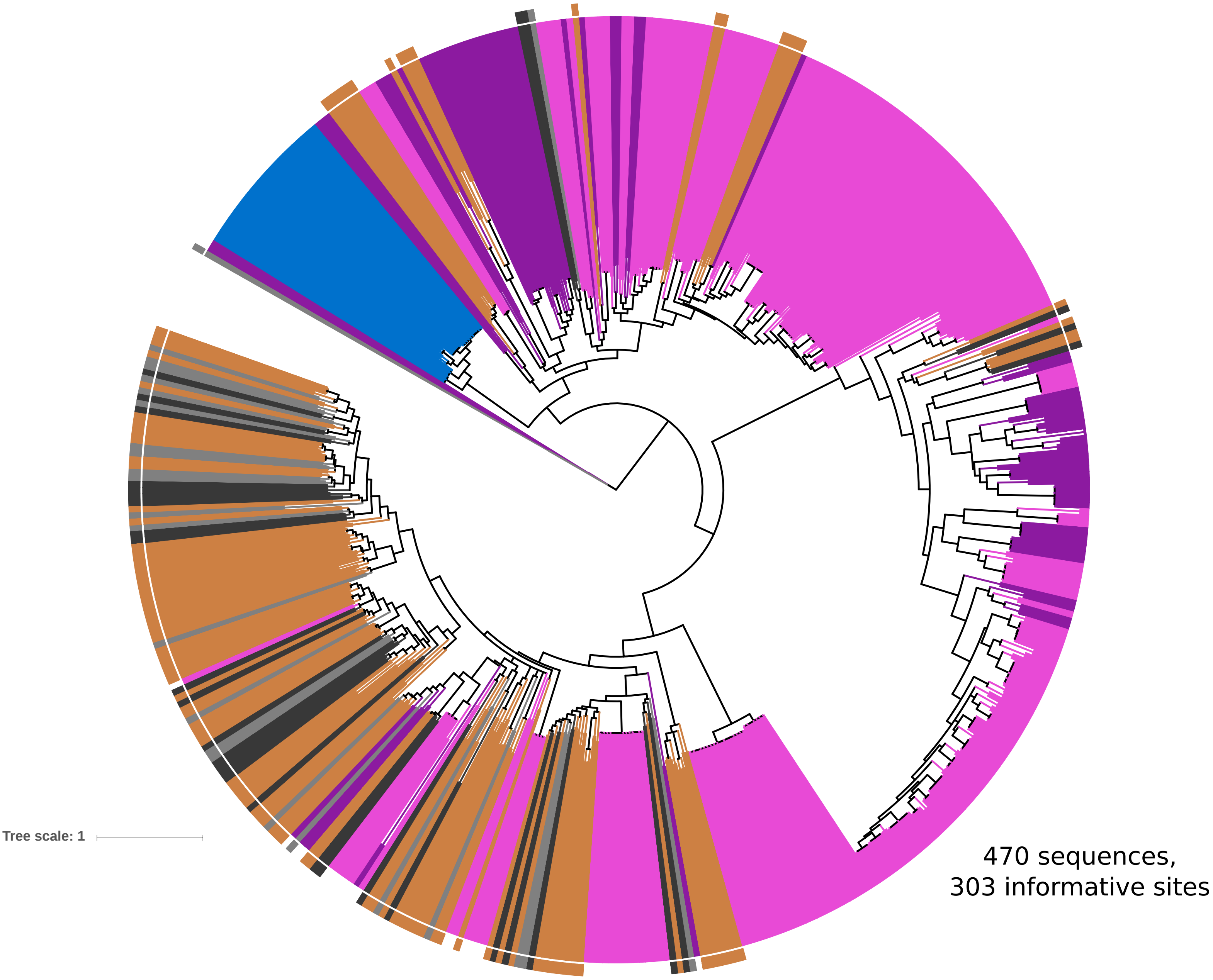

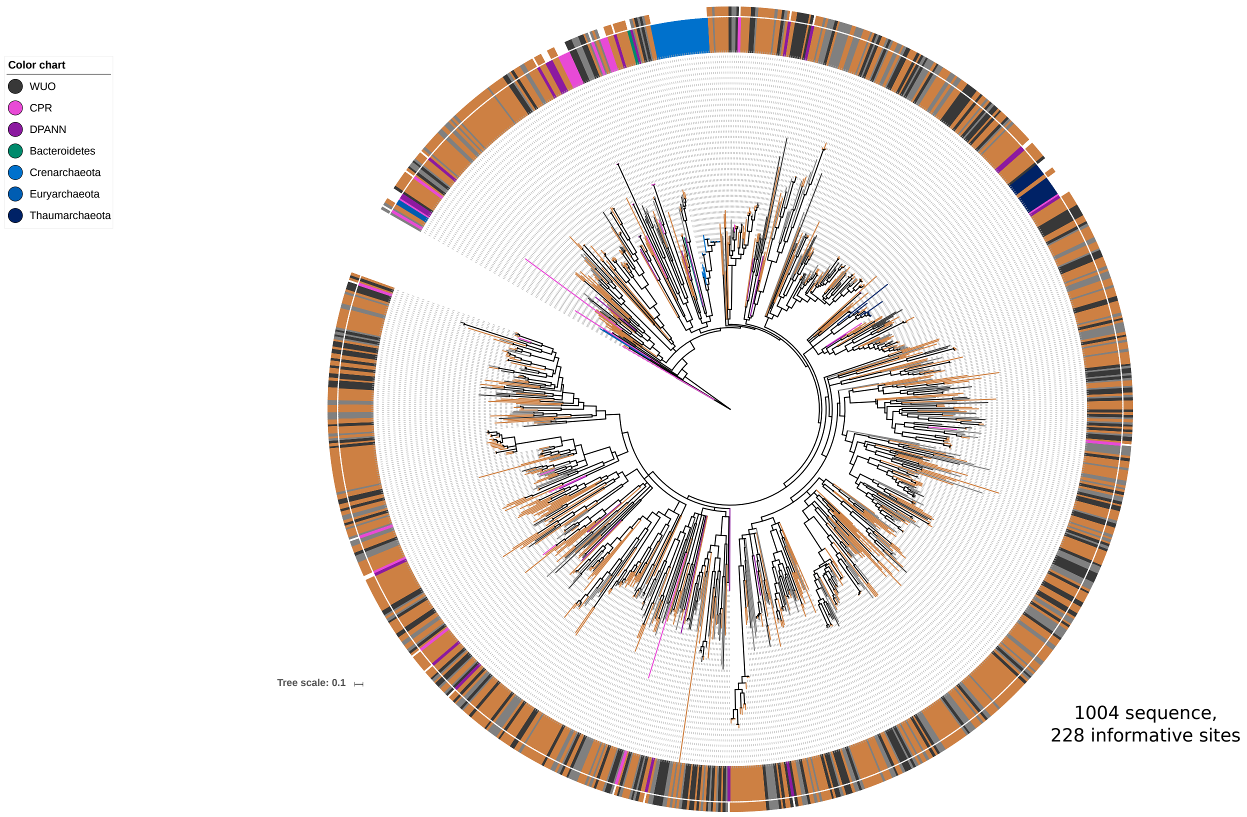

Color chart
WUO
CPR
DPANN
Bacteroidetes
Crenarchaeota
Euryarchaeota
Thaumarchaeota
Tree scale: 0.1
1004 sequence,
228 informative sites

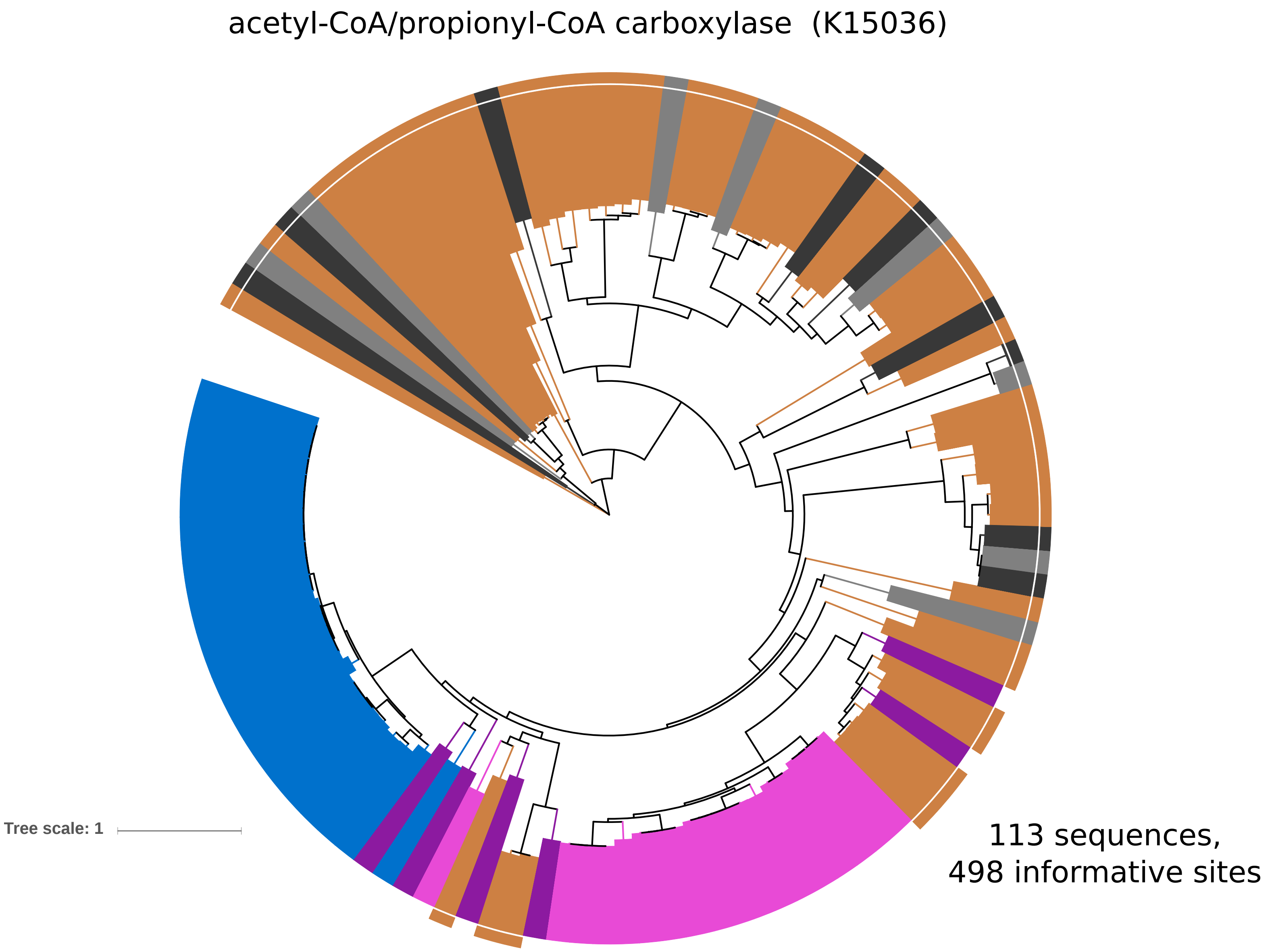
acetyl-CoA/propionyl-CoA carboxylase (K15036)
Tree scale: 1
113 sequences,
498 informative sites

ATP-citrate lyase alpha-subunit (K15230)

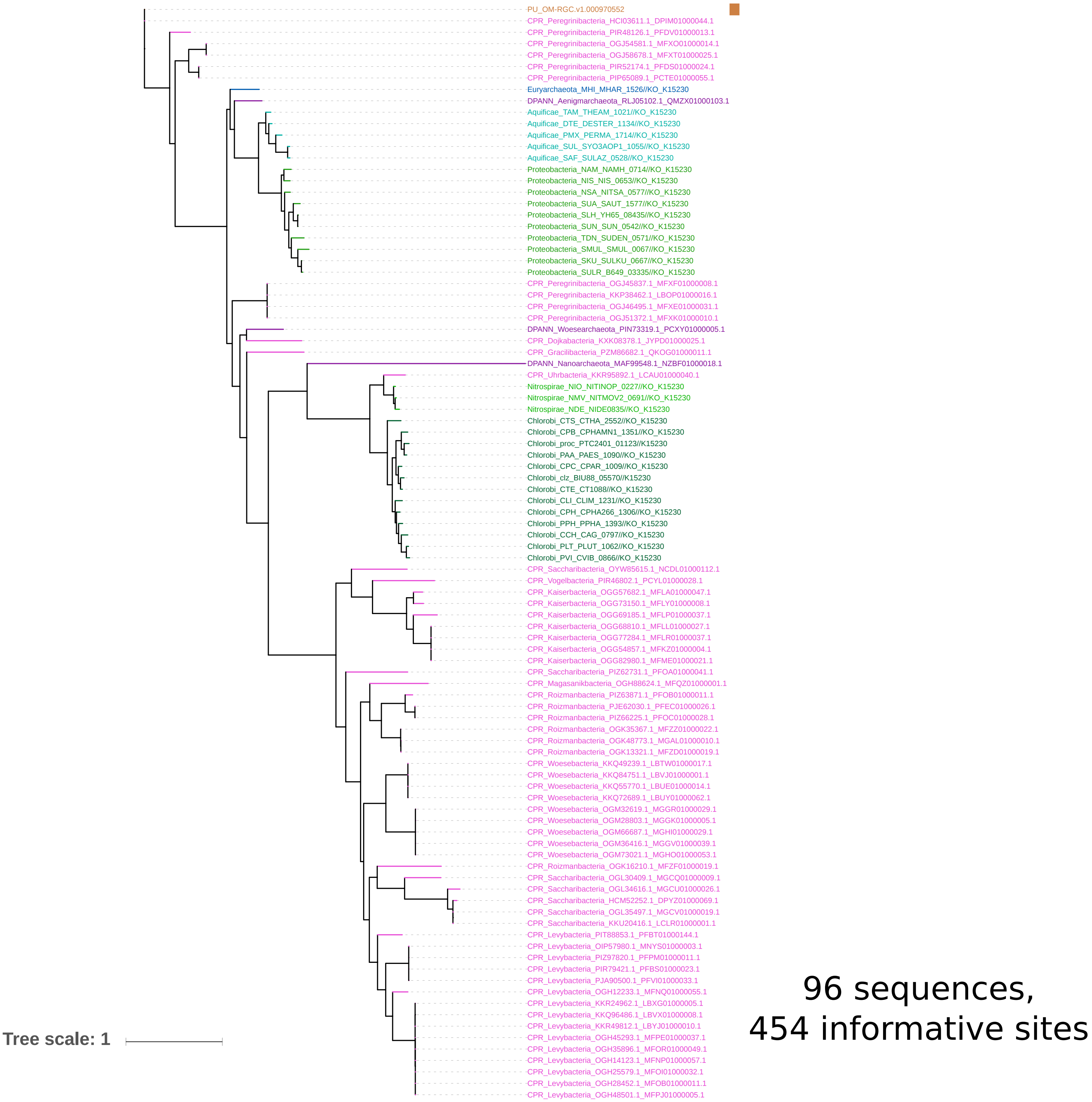

ATP-citrate lyase beta-subunit (K15231)

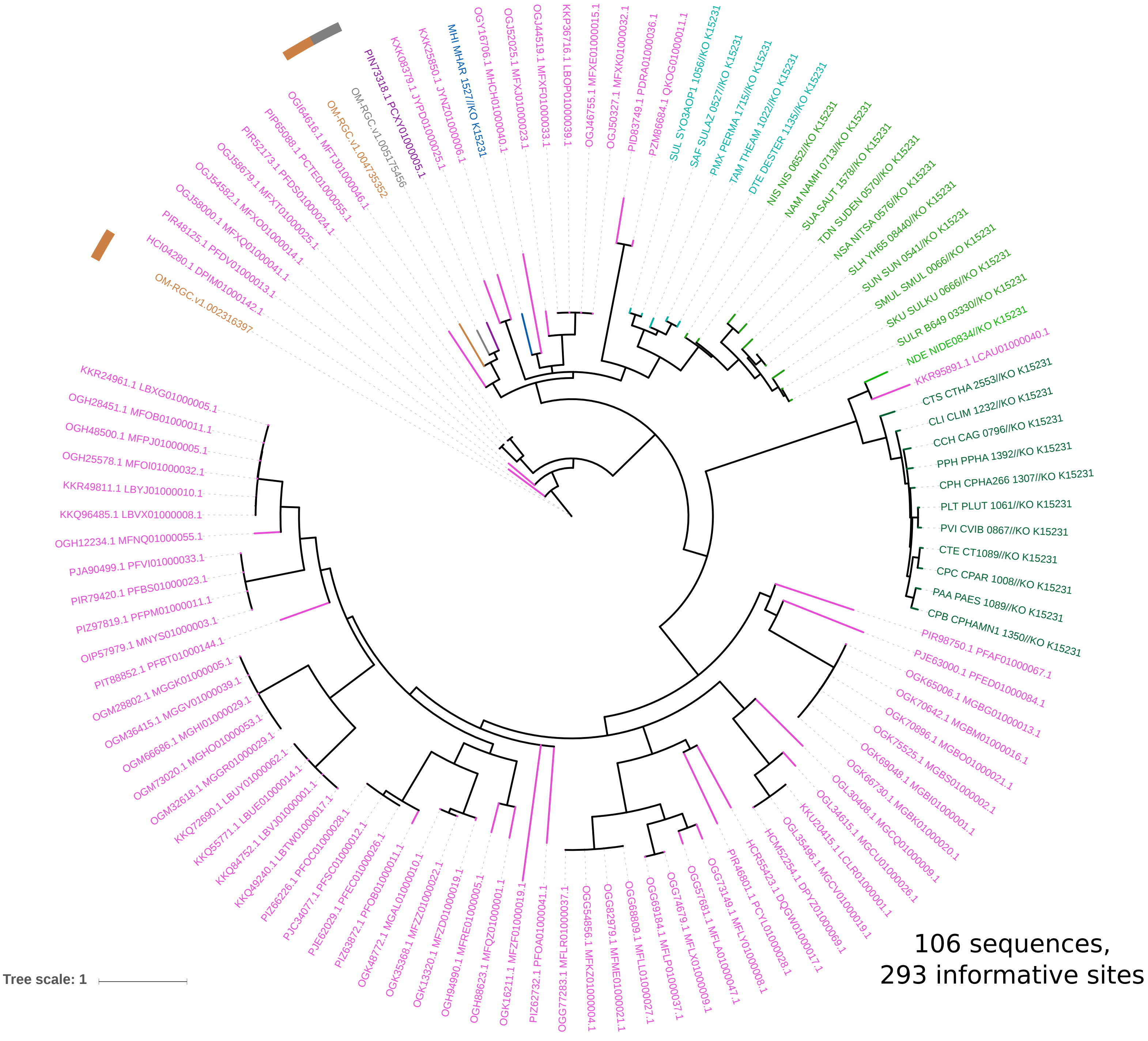

Supplement: Supplement_Material_evz050 [file supplement_material_evz050.zip › SI_FIg2.pdf]
