# Supplementary material for: Carbon Fixation by Marine Ultrasmall Prokaryotes
Source: Genome Biol Evol. 2019 Mar 23;11(4):1166–77. doi: 10.1093/gbe/evz050 (PMC6475129; doi:10.1093/gbe/evz050)
Supplement: Supplementary file 4 [file supplement_material_evz050.zip › SI_Legend.odt]

SI Legend:

SI table 1: p-values of Fischer exact tests from Figure 2

The p-values corresponding the Fischer exact tests from Figure 2 are given here.

SI table 2: description of the metabolic enzymes under study

For each autotrophic carbon fixation pathway, this table summarizes what enzymes take part in a given pathway, along with their KEGG identifier, common names, E.C. numbers, and specifies if an enzyme is a "key enzyme" (specific) of the pathway.

SI Table 3: Correlation of sequencing effort to pathways’ completeness 

For each dataset we computed the correlation between : number of reads, number of high quality reads,  number of genes, average number of  reads per gene and metabolic pathways and ribosomal complexes. The table shows the Spearman correlation values (Rho) and the associated p-value. Correlation was computed with Scipy.stats in python3.

SI Fig 1: Trees of ribosomal proteins

Sequences were aligned using MAFFT in auto mode and trimmed with trimAl. Trees were reconstructed using IQ-TREE with LG + G4 models and ultrafast bootstrap approximation (Minh et al. 2013). Trees were rooted between Archaea and Bacteria and branches with bootstrap values lower than 50% were collapsed. The number of informative sites and branch length scale bars (substitutions per site) are shown. Environmental sequences are highlighted by a colored bar in the outer ring. Sequences come from three sources: environmental sequences come from TARA Oceans datasets, CPR and DPANN sequences are from assemblies available in NCBI, others reference sequence are from KEGG. Sequences are colored by taxonomic annotation. Archaeal sequences found in 037, 038, 039 MES sampling sites are highlighted by a red arc.

Abbreviations: PU: Potentially Ultra-small, UO: Ultra-small Only, WUO: Widespread Ultra-small. Trees with more than 10,000 leaves are not shown. Three very large trees (K02881_K02932_K02933_K02940_K02981_K02988, K00297, K02939_K02963_K02990) were reconstructed using FastTree and therefore do not have bootstrap values.

Abbreviations: PU: Potentially Ultra-small, UO: Ultra-small Only, WUO: Widespread Ultra-small.

SI Fig 2: Trees of various carbon fixation enzymes 

Protocols and color codes are the same as those of SI Figure 1. Two very large trees (K00134, K00927) were reconstructed using IQ-TREE -fast option and therefore do not have bootstrap values.

Abbreviations: PU: Potentially Ultra-small, UO: Ultra-small Only, WUO: Widespread Ultra-small.
